# Integrated network pharmacology and transcriptomics to explore the mechanism of compound Dihuang granule (CDG) protects dopaminergic neurons by regulating the Nrf2/HMOX1 pathway in the 6-OHDA/MPP^+^-induced model of Parkinson’s disease

**DOI:** 10.1186/s13020-024-01040-7

**Published:** 2024-12-18

**Authors:** Xue Sun, Shuai Yang, Zhuqing He, Li Wang, Jiancheng He

**Affiliations:** 1 School of Traditional Chinese Medicine, Shanghai University of Traditional Chinese Medicine, Shanghai, 201203 China; 2 Shanghai Municipal Hospital of Traditional Chinese Medicine, Shanghai University of Traditional Chinese Medicine, Shanghai, 200071 China; 3 Shanghai Key Laboratory of Health Identification and Assessment, School of Traditional Chinese Medicine, Shanghai University of Traditional Chinese Medicine, Shanghai, 201203 China; 4 Department of Endocrinology and Metabolism, Putuo Hospital, Shanghai University of Traditional Chinese Medicine, Shanghai, 200062 China

**Keywords:** Compound Dihuang granules, Parkinson’s disease, Ferroptosis, Nrf2/HMOX1 pathway

## Abstract

**Background:**

Parkinson's disease (PD) is a degenerative neurological disease that worsens over time. Ferroptosis has been proven to contribute to PD pathogenesis. CDG exhibits neuroprotective effects. However, CDG's potential mechanism in PD therapy remains uncertain.

**Purpose:**

The purpose of this investigation is to ascertain the specific molecular mechanisms of CDG against neuronal ferroptosis and present an alternative option for PD management.

**Methods:**

Network pharmacology along with LC–MS were used to identify possible targets and candidate pathways. Then RNA-sequencing combined in the in vitro and in vivo experiments were utilized to validate these findings.

**Results:**

According to network pharmacology prediction, NFE2L2, HMOX1 and PTGS2 may be the key genes for ferroptosis in PD. In the in vivo experiments, CDG ultimately improved the neurobehavior of PD rats by alleviating the damage of dopamine neurons, decreasing the levels of MDA, ROS and Fe^2+^, increasing the GSH level, inhibiting ferroptosis by decreasing ACSL4, TF, and PTGS2 expression levels, and increasing the GPX4, FTH, Nrf2, and HMOX1 levels. RNA-seq analysis showed the differential genes in Model and CDG group were all enriched in Nrf2 and HMOX1, and the enrichment analysis of these differential genes showed they were closely related to the ferroptosis. Subsequently, in vitro experiments, the CDG, OE-Nrf2 and OE-HMOX1 group showed more active cell vitality, with decreasing levels of MDA, ROS, Fe^2+^, ACSL4, TF and PTGS2, and increasing level GSH, GPX4, FTH, Nrf2 and HMOX1.

**Conclusion:**

CDG has a neuroprotective involvement in alleviating ferroptosis by regulating the Nrf2/HMOX1 pathway. Moreover, this research offers pharmacological evidence supporting the applications of CDG for treating PD.

**Supplementary Information:**

The online version contains supplementary material available at 10.1186/s13020-024-01040-7.

## Introduction

Parkinson's disease (PD) is a gradually advancing, the second most prevalent neurological condition that impacts 2–3% of those aged 65 and above [1]. The 2017 Global Burden of Disease research reports that PD exhibited the fastest growth among all neurological disorders, with a clinically diverse spectrum of motor and non-motor symptoms [2]. The development of PD involves a complicated process, characterized by progressive degeneration of dopaminergic (DA) neurons in the SNpc and accumulation of Lewy bodies [3]. Currently, Levodopa remains the main treatment option for PD; however, within 5–10 years of levodopa treatment, 80% of PD patients will develop dyskinesia [4]. Thus, it has become urgent to find novel and effective therapeutic agents for PD.

In recent years, researchers focused on oxidative stress [5], mitochondrial dysfunction [6], inflammatory processes [7] and autonomic dysfunction [8] that might be involved in PD pathogenesis. Upon further investigation, it has been revealed that ferroptosis plays an essential role in the pathogenesis of PD, specifically in the demise of dopaminergic neurons. Iron overload and reactive oxygen species (ROS) derived from lipid peroxidation are the main characteristics of ferroptosis [9]. In addition, the abnormal deposition of iron in PD patients suggests a close association between ferroptosis and PD [10].

Nuclear transcription-related factor 2 (Nrf2) is the primary transcription factor that controls antioxidant responses [11]. A growing body of evidence has implicated Nrf2 in ferroptosis pathogenesis [12]. It is currently unclear how Nrf2 inhibits ferroptosis [13]. Heme oxygenase-1 (HMOX1) is a vital enzyme involved in heme metabolism, and it has significant implications for iron metabolism and the body's response to oxidative stress [14, 15]. Importantly, HMOX1, an important Nrf2 target gene, is regulated by Nrf2 [16]. Studies have revealed that the Nrf2/HMOX1 pathway activation showed neuroprotective implications, suggesting that it can be a meaningful therapeutic target [17, 18]. Therefore, the Nrf2/ HMOX1 signaling pathway could be a possible drug target for treating dopaminergic neuronal death and iron-induced neuronal toxicity.

Traditional Chinese Medicine (TCM) manifests distinct benefits and advantages in preventing and clinically treating PD [19–21]. Compound Dihuang granule (CDG) is an empirical prescription and effective prescription for PD, emerging with a clear impact in improving PD symptoms and reducing adverse action rates [22]. The prior research has shown that CDG inhibits cell apoptosis in the substantia nigra striatum pathway and alleviates PD symptoms [23].Several active ingredients of CDG have preventive implications by inhibiting ferroptosis in PD models [24, 25]. However, the complex nature of TCM prescriptions, which have several targets and multiple effects, makes it difficult to understand the specific processes underlying the implications of CDG on PD. Moreover, the underlying mechanisms of CDG in modulating ferroptosis remain unknown.

Network pharmacology is a promising approach for accelerating drug development and elucidating the mechanisms of multiple target components. To obtain a better understanding of the positive impacts of CDG on PD, we used multiple authoritative databases with high prediction accuracy and combined Liquid chromatography-mass spectrometry (LC–MS) to find the potential therapeutic compounds of CDG prescription and clarify the key targets and potential mechanism by which CDG acts on PD. They were also confirmed by RNA sequence analysis and investigations performed in the in vivo and in vitro. The potential mechanism behind CDG's neuroprotective effect in suppressing ferroptosis in PD is linked to its capacity to control the Nrf2/HMOX1 pathway.

## Materials and methods

### Animals and reagents

Sprague Dawley rats, aged 8 weeks and weighed 180 ± 20 g, were obtained from the animal experimental center at Shanghai University of TCM (license No. SYXK (Hu) 2020-0009). The SH-SY5Y cell line were obtained from the Shanghai Cell Bank, Chinese Academy of Sciences (Shanghai). The compounds 6-Hydroxydopamine hydrobromide (6-OHDA, MKBP0832V), Apomorphine (APO, SLBF6369V), and L-Ascorbic acid (063K1082) were obtained from Sigma Chemicals (St Louis, USA). Pentobarbital sodium (WS20130112) was procured from Shanghai Chinese and Western Pharmaceutical Co., Ltd. MPP^+^ (HY-W008719) from Med Chem Express (New Jersey, USA). The following antibodies were purchased from Proteintech (Wuhan Biotechnology Co., Ltd, China): Transferrin Polyclonal antibody (Cat No. 17435-1-AP), ACSL4/FACL4 Polyclonal antibody (Cat No. 22401-1-AP), COX2/PTGS2 Monoclonal antibody (Cat No. 66351-1-Ig), GPX4 Monoclonal antibody (Cat No. 67763-1-Ig), Ferritin light chain Polyclonal antibody (Cat No. 10727-1-AP), TH Polyclonal antibody (Cat No. 25859-1-AP), and Alpha Actin Polyclonal antibody (Cat No. 23660-1-AP). Cell Signaling Technology was the source of the Anti-mouse IgG (#5257) and Anti-rabbit IgG (#5151) antibodies. MDA detection kits, ROS detection kits, GSH detection kits, and Iron ion detection kits (Shanghai Weiao Biotechnology Co., Ltd, China).

### CDG preparation

CDG is made up of seven herbs: Rehmannia glutinosa (Gaertn.) DC. (Voucher numbers CDG01-130306), Paeonia lactiflora Pall (CDG02-DH2012071703), Uncis Uncaria rhynchophylla (Miq.) Miq. ex Havil (CDG03-HY2012102204), Hyriopsis cumingii (Lea) (CDG04-HY2012040501), Salvia miltiorrhiza Bge (CDG05-YT2012091506), Acorus tatarinowii Schott (CDG06-LY2012080321) and Buthus martensii Karsch (CDG07-YT2012092411). CDG, with the lot number 20220701, made by Shanghai Wanshicheng Pharmaceutical Co., Ltd. To summarize, finely chop the herbs and immerse them in distilled water for the duration of about 30 min. Then, the items were routinely subjected to two rounds of boiling, each lasting one hour. Begin by immersing them in a solution consisting of 10 volumes of distilled water, followed by a second round in a solution consisting of eight volumes of distilled and deionized water. Samples were gathered, refined and then condensed into a cream with a specific gravity of 1.3. The preparation undergoes a process of drying, sieving, and subsequent pulverization into the CDG. As described in the 2010 edition of China Pharmacopoeia, all of these were compliant with the standard.

### Identification of major chemical components of CDG by LC–MS

The quality of CDG was evaluated by LC–MS [41]. We used a Thermo Scientific Hypersil Gold C18 chromatographic column (2.1 mm × 100 mm × 1.9 m). A mobile phase contains water and 0.1% formic acid; B is acetonitrile and 0.1% formic acid, with an elution gradient of 0–5 min and 5–15% B; 5–8 min, 15–17% B; 8–16 min, 17–20% B; 16–18 min, 20–95% B; 18–19 min, 95% B; 19–19.1 min, 95–5% B; 19.1–20 min, 5% B; Column temperature 35 ℃, injection volume 5 μL. Mass spectrometry condition: electric spray ion source, scanning mode: negative ion scanning (ESI−, m/z 50–1000); heater temperature: 300 ℃; Sheath gas flow rate 45 psi (1 psi ≈ 6.895 kPa); Auxiliary airflow speed 5 L/min; Exhaust gas flow rate of 0.3 L/min; Electric spray voltage − 3.2 kV; The capillary temperature is 350 ℃.

### Prediction of putative CDG prescription targets

The chemical constituents of CDG are from HERB 2.0 database (http://47.92.70.12/) and the Batman 2.0 (http://bionet.ncpsb.org.cn/batman-tcm/). The compounds in above database are intersected with the compounds measured by LC–MS to get the candidate compounds of CDG. Typical SMILES structures corresponding to the active components were obtained from the organic small molecule biological activity database PubChem (https://PubChem.ncbi.nlm.nih.gov/). SMILES structure obtained from PubChem was imported into Swiss adme (http://www.swissadme.ch/) database, and the drug structure was predicted, so as to obtain the target of candidate components.

### PD-associated therapeutic targets

With "Parkinson's Disease" as the search term, PD-related targets were collected from the following six databases. GeneCards (Relevance score ≥ 2) (https://www.genecards.org/), Drugbank (https://go.drugbank.com/#), CTD (Inference Score ≥ 50) (https://ctdbase.org/), Disgenet (score_gda ≥ 0.01) (https://www.disgenet.org/), pharmgkb (https://www.pharmgkb.org/), OMIM (https://omim.org/), after deleting duplicates, use UniProt database for correction.

### Network pharmacology analysis

In order to explore the potential effect of CDG on PD, the candidate targets of CDG and the potential therapeutic targets of PD were intersected, and the common targets were obtained by Venn diagram. To evaluate the importance of these targets, STRING (https://cn.string-db.org/) was used to import these potential targets. We defined the species as "Homo sapiens", defined the interaction score as > 0.900, and removed the single targets without interaction. Eigenvector, Betweenness and Closeness are calculated by using CytoNCA plug-in of Cytoscape 3.10.2, and the median of the results is taken as the screening threshold, and the targets exceeding the threshold are regarded as key targets.

### Functional enrichment analysis

We introduced the candidate components of CDG into Metascape database (https://metascape.org//) for pathway enrichment. Select gene ontology, GO), Kyoto encyclopedia of genes and genomes, KEGG), Hallmark Gene set, Reactome Gene set, WikiPath, Oncogenic Signatures, Transcription Factor Targets, DisGeNET database are analyzed, and the obtained paths are visualized by R language package ggplot2.

### Animal grouping and model establishment

The male Sprague Dawley rats employed in this investigation were raised in a research facility located at Shanghai University of TCM. The animals were housed individually in cages containing 6–8 individuals. They had unrestricted access to food and water. The humidity was preserved at 60–65%, the temperature was kept at 23 ± 2 °C, and they were maintained under a 12-h dark/light cycle.

The rats were deprived of water for 12 h before surgery, and no abnormal neurobehavioral tests were confirmed before surgery. Concerning previous studies, two coordinates of the right substantia nigra were determined (5.2 mm behind the fontanel, 1.0 mm right of the median line, 9.0 mm subdural; 5.2 mm posterior fontanel, 2.5 mm right median line, 8.5 mm subdural). Rats anesthetized with 3% pentobarbital sodium (50 mg/kg) were fixed in a brain stereoscope, shaved and disinfected at the cranial top, incised along the median line, peeled periosteum, exposed the cranial top, drilled holes in the skull, and inject 6-OHDA (dissolved in saline containing 0.02% ascorbic acid, 2 μg/μL) into the hole with a micro sampler (injection speed 1.0 mm/min), 3 μL per hole, the injection speed was 1 μL/min, the needle stayed in place for a 5-min interval before being retracted at 1.0 mm/min. The cranial hole was filled with medical gelatin sponge, the incision was sutured, and 200,000 units of gentamicin were injected intramuscularly for 7 days to prevent intracranial infection, and the rats were put into a feeding cage after waking up. The Sham group received no treatment except fixed rats. Ten days after surgery, the rats were induced to rotate to the healthy side by intraperitoneal injection of APO (0.5 mg/kg), and the number of rotations from the beginning of rotation to the end of 30 min was recorded, and patients with > 7 rotations/min were considered as successful animal models of PD [26].

Firstly, we randomly assigned 44 successful model rats to four groups: Model, CDG, Erastin + CDG, and Fer-1. Additionally, 11 normal rats were included in the Sham group. The final group was divided as follows: (1) Sham group: received gavage of normal saline (1 mL/100 g) and intraperitoneal injection of a 10% DMSO solution. (2) Model group: received gavage of normal saline (1 mL/100 g) and intraperitoneal injection of a 10% DMSO solution. (3) CDG group: received intragastric administration of CDG (7 g/kg/d) and intraperitoneal injection of a 10% DMSO solution. (4) Erastin + CDG group: received intragastric administration of Erastin (20 mg/kg/d) and CDG (7 g/kg/d) and intraperitoneal injection of a 10% DMSO solution. (5) Fer-1 group: received intraperitoneal injection of Fer-1 (2.5 μmol/kg/d) and intragastric administration of normal saline (1 mL/100 g). All rats were treated continuously for 4 weeks.

Secondly, for evaluating the mechanism of CDG, 66 rats with successful Model were randomly divided into Model group, CDG group, ML385 + CDG group, Znpp + CDG group, Oltipraz group, Hemin group, and 11 normal rats were included as Sham group. Normal saline (1 mL/100 g) was given by gavage to the rats in Sham group and Model group; and rats in CDG group were given CDG (7 g/kg/d) by gavage, rats in ML385 + CDG group were oral administrated of ML385 (20 mg/kg/d) and CDG (7 g/kg/d); Similarly, Znpp (10 mg/kg/d) and CDG (7 g/kg/d) were intragastrically given to the rats in Znpp + CDG group; Oltipraz (15 mg/kg/d) was given by gavage to the rats in Oltipraz group; Hemin (50 mg/kg/d) was given by gavage to the rats in Hemin group; and 10% DMSO solution was injected intraperitoneally to all rats and treated continuously for 4 weeks.

### Behavioral test

#### Rotation test

The changes in healthy side rotation behavior induced by APO (0.5 mg/kg, sc.c.) were measured at 0, 14, and 28 days after treatment, respectively, and the number of rotation laps from the beginning of rotation to the end of 30 min was recorded.

#### Suspension test

On the 28th day of the intervention, suspension experiments were performed on the rats of each group at the same time, 24 h after gavage [27]. Hang the rat's front PAWS on a metal wire placed horizontally 30 cm above the ground, record the time from the beginning of hanging to the landing, and score the score as follows: the duration of approximately 0–5 s is 0 point, 6–10 s is 1 point, 11–15 s is 2 points, 16–20 s is 3 points, 21–25 s is 4 points, 26–30 s is 5 points, and more than 30 s is 6 points. A total of 3 detection times are taken as an average, and the interval of each detection is about 2 min.

### Pole climbing experiment

A foam ball measuring 2 cm in diameter is securely attached to the apex of a wooden pole that is 50 cm long and has a diameter of 1 cm. To prevent slippage, encase the hardwood pole in two layers of gauze. Hold the model rat by its tail, place its head on top of the pole (ensuring that the rat's hind legs are on the ball), and allow it to climb down naturally. The rat climbs completely from standing on the top of the pole to the platform at the bottom of the pole with two front legs. On the 28th day of the intervention, the rats were tested 24 h after intragastric administration, and the climbing time and climbing score were recorded, respectively. Scoring criteria: use all four limbs; a successful climb from the pole is 0 point; Step-by-step spiral downward crawling with sliding hind legs behavior is 0.5 point; After intermittent pause several times after climbing down, but can hold the metal pole is 1 point: sliding after falling is 1.5 points; Can not grab the pole, drop directly to 2.0 points. The exercise score and the overall exercise duration from the start of the exercise to crawling to the bottom of the rod were recorded, and the average value was measured 3 times, each time with an interval of 5 min. If the rats stopped or reversed crawling in the middle of the course, they were not recorded and re-measured [28].

### Cell culture

SH-SY5Y neuroblastoma cells were obtained from the Shanghai Institute of Cell Biology. 43% Ham's F-12 Nutrient Mixture (ICELL, Shanghai, China), 43% culture medium, 10% fetal bovine serum (Gibco, Grand Island, NY, USA), 1% non-essential amino acids, 1% Sodium Pyruvate, 1% GlutaMAX-1, and 1% penicillin–streptomycin (BIOSHARP, Anhui, China). Humidified incubator conditions comprised 5% CO_2_ and 37 °C.

### Nrf2/HMOX1 knockdown in SH-SY5Y cells

When SH-SY5Y cells grew to 80%–90%, the cells were digested and counted, and SH-SY5Y cells were inoculated into 12-well plates with 2 × 10^5^ cells/well. 24 h later, the viral stock was extracted from the – 80 ℃ freezer and thawed in the ice bath. The stock virus stock was diluted with 800 μL complete medium according to MOI = 10, the original medium of the treatment group was absorbed, and the 500 μL medium containing the diluent of chronic disease venom was added to the cells of the treatment group. After 24 h, the culture medium was changed, and following 16 h of infection, the culture medium containing lentivirus was completely changed into a 1 mL complete culture medium. After 48 h, the suitable eukaryotic resistance screening cells (total lethal concentration of Blasticidin 20 μg/mL, maintenance concentration of 10 μg/mL) were selected, and the cells were rendered stable after two cycles of comprehensive drug screening, with each cycle lasting for three days, as established by an antibiotic pre-test. After stabilization, the basal maintenance cells were completely cultured with F12K + 10% FBS + 1% P.S + 10 μg/mL Blasticidin (Sigma 15205 St Louis, USA). After the cells were overgrown, the digestive cells were expanded and cultured, and part of the cells were extracted for western blot identification.

### Overexpression of Nrf2/HMOX1 in SH-SY5Y cells

H_NRF2/H_HMOX1 SH-SY5Y cells were inoculated with 2 × 10^5^ cells/well in a 12-well plate. 24 h later, the viral stock was extracted from the − 80 ℃ refrigerator and thawed in the ice bath. The stock virus stock was diluted with 800 μL complete medium according to MOI = 10, the original medium of the treatment group was absorbed, and the 500 μL medium containing the diluent of chronic disease venom was added to the cells of the treatment group. After 24 h, the culture medium underwent a change, and after 16 h of infection, the culture medium containing lentivirus was completely changed into a 1 mL complete culture medium. After 48 h, we selected eukaryotic cells that were suitable for resistance screening. The fatal value of Blasticidin was 20 μg/mL, and the concentration was 10 μg/mL. 0.5 μg/mL puromycin was added, and the maintenance dose was 0.25 μg/mL. We performed two cycles of comprehensive drug screening, with each cycle lasting for 2 days, as specified, in order to stabilize the cells. The cells were fully sustained after stabilization using a culture medium consisting of F12K enriched with 10% FBS, 1% PS, 10 μg/mL Blasticidin, and 0.25 μg/mL Puromycin.

### Cell treatment

Cultured SH-SY5Y cells were subjected to MPP^+^ at various doses for 24 h, then, the CCK-8 assay was conducted to establish the optimal dosage concentration for the damage model. Subsequently, in the coculture of MPP^+^, different concentrations of CDG were infused with the culture medium for 24 h, and CCK-8 was determined. Finally, the cells are grouped as follows: (1) Sham group (PBS), (2) Model group: 2 mM/mL MPP^+^, (3) CDG group: MPP^+^ (2 mM/mL), CDG (200 μg/mL); (4) Fer-1 group: 2 mM/mL MPP^+^  + 10 μm/L Fer-1, (5) DFO group: 2 mM/mL MPP ^+^  + 50 μm/L DFO. All cells were incubated at 37 ℃ with 5% CO_2_ for 24 h.

Grouped the cells as follows: (1) Sham group (PBS), (2) Model group (2 mM/mL MPP^+^), (3) CDG group: MPP^+^ (2 mM/mL), CDG (200 μg/mL); (4)sh-Nrf2 + CDG group: MPP^+^ (2 mM/mL), CDG (200 μg/mL); (5) sh-HMOX1 + CDG group: MPP^+^ (2 mM/mL), CDG (200 μg/mL);(6) OE-Nrf2 group (2 mM/mL MPP^+^), (7) OE-HMOX1 group (2 mM/mL MPP^+^). All cells were incubated at 37 ℃ with 5% CO_2_ for 24 h.

### Transmission electron microscopy (TEM)

The pathological changes of mitochondria in the rat striatum were observed under an electron microscope. After the rats were anesthetized, fresh rat striatal tissue (1 mm^3^) was taken; the striatal tissue was washed with phosphate buffer solution (PBS) and then preserved overnight at 4 °C using a 2.5% glutaraldehyde solution. The specimens were rinsed with PBS for a duration of 30 min, then fixed with a 1% osmium tetroxide solution for a period of 2 h, and then subjected to dehydration using a gradient of ethanol, impregnated with epoxy resin, embedded, and then ultrathin sections (60 nm) stained with lead citrate and uranium, and finally observed using TEM, images were collected and analyzed.

The pathological changes of mitochondria in SH-SY5Y cells were observed under an electron microscope. After the treatment of each group of cells, the cells with the size of rice grains were collected by digestion and centrifugation with pancreatic enzyme, fixed at 4 ℃ overnight with propylene glycol, rinsed with PBS, and then the samples were embedded after gradient dehydration with acetone and immersion with acetone and resin. The ultrathin sections were prepared, stained by lead-uranium, observed under an electron microscope, and photographed.

### Immunohistochemistry (IHC)

The level of TH of rats was quantified by immunohistochemistry. The brains of rats were injected into the heart under anesthesia, fixed with 4% paraformaldehyde solution, and the mesencephalon nigra and striatum tissue with a thickness of about 2 mm in coronal position was taken. The tissue was dehydrated, transparent, impregnated with wax, and then embedded in paraffin wax. A continuous coronal section with a thickness of about 5 μm was made by a paraffin micro-slicing machine. After antigen repair, sealing, antibody incubation, color rendering, sealing, and microscopic examination, in each section, the dense area of TH-positive cells was selected at low magnification, and then 3 non-adjacent fields were randomly selected and photographed at 400 times magnification. The average optical density was analyzed and averaged using Image-Proplus 6.0 software.

### RNA-sequencing analysis

The extraction of total RNA from the rat striatum was performed employing Trizol reagent (thermofisher, 15596018) following the manufacturer's recommendations. The amount and purity of the total RNA were ascertained by employing the Bioanalyzer 2100 and the RNA 6000 Nano LabChip Kit from Agilent (CA, USA, 5067-1511). Subsequently, sequencing libraries were prepared to employ high-quality RNA samples with RIN values of more than 7.0. Ultimately, we conducted paired-end sequencing (PE150) with 2 × 150 bp read length using the Novaseq 6000 platform (Illumina) (LC-Bio Technology CO, Ltd, Hangzhou, China). Analyze the data via Unicawa Bio's complimentary online omicstudio (www.omicstudio.cn) or R program. A fold change (FC) value ≥ 2 and an adjusted p value of ≤ 0.01 were the criteria for identifying differentially expressed genes (DEGs).

### Immunofluorescence (IF)

The expression colocalization of Nrf2, HMOX1, and TH in substantia nigra was detected by immunofluorescence. In the in vivo experiment, the brain slices that were pre-cut for immunohistochemistry were rinsed with PBS three times. Subsequently, each section was treated with 1% BSA for blocking for a duration of 1 h. Following that, primary antibodies targeting Nrf2 (1:200) or HMOX1 (1:100) and TH (1:500) were introduced and left to incubate at a temperature of 4 ℃ overnight in addition to 1 h at room temperature for the second antibody; after washing, a long-acting anti-fluorescence quencher containing DAPI was added, and the slide was observed under the Olympus BA51 microscope. SH-SY5Y cells were inoculated on 24-well plate slides in vitro. After culture for 48 h, drug intervention for 24 h, PBS washing for 3 times, methanol fixation for 15 min, 1% BSA sealing for 30 min. Primary antibodies Nrf2 (1:1000), HMOX1 (1:1000), and TH (1:3000) were incubated at 4 ℃ overnight. The second antibody (1:1000) was incubated at a temperature of 37 ℃ for 60 min on the next day, washed with PBS 3 times (3 min each time), and then followed by staining with DAPI for 10 min at 37 °C. Cells were detected by IF.

### Biochemical index detection

In the in vivo experiment, the supernatant extracted from rat striate homogenate was stored at − 80 ℃. ROS (ER9407M), Fe^2+^ (ER9292M), MDA (ER9405M), and GSH(ER9290M) in rat striate tissue samples were quantified using a commercial kit based on the manufacturer's recommendations (Shanghai Weiao BioEngineering, China). In the in vitro experiment, the cells were gathered into a centrifuge tube. After being spun in a centrifuge, the supernatant was removed. Then, 1 mL of PBS was added, and the cells were disrupted using ultrasonic waves (ice bath, power 200 W, ultrasonic 3 s, interval 10 s, repeated 10 times). Centrifuge 12,000 r for 10 min at 4 ℃, take the supernatant, and put it on the ice to be measured. The contents of Fe^2+^ (BYSH-1212W), MDA (BYSH-0109W), and GSH(BYSH-0206F) were determined according to the instructions of the manufacturer (Nanjing Bo Yan Biotechnology Co., Ltd., China) for testing cell samples with commercial kits.

### Live/dead cell staining

Cell viability was measured using live/dead staining. The reagents in tubes A and B were mixed according to the reagent instructions, mixed into the cells to be tested in a single culture at 1:9, placed at 37 ℃ for 30 min, and then photographed under a fluorescence microscope. Living and dead cells fluoresce green and red, respectively.

### ROS detection

Intracellular ROS were detected using DCFH-DA fluorescent probes (Dojindo, R252, Shanghai, China). The cells were first inoculated in 96-well plates and cultured in incubators for 48 h. After incubation in the incubator for 24 h, the supernatant was removed, the cells were washed with HBSS twice, and then DCFH-DA fluorescent working liquid was added into the hole and cultured in the incubator for 30 min. Remove the working solution, wash the cells with HBSS twice, then add HBSS, and observe and take photos with a fluorescence microscope.

### Western blot analysis

Rat striatum tissue or SH-SY5Y cells were cleaved by adding 1 × RIPA cleavage buffer containing a mixture of protease inhibitors. Preparation of denatured protein samples, and based on the manufacturer's recommendations (Beyotime, P0001, Shanghai, China) determination of protein concentration. The sample (20 μg/lane) was separated via SDS-PAGE gel electrophoresis and then transferred onto a polyvinylidene fluoride (PVDF) membrane, and which was sealed at ambient temperature for 1 h, employing a Tris-buffered brine solution with 0.1% Tween^®^20 detergent (TBST) buffer with 5% skim milk. Subsequently, the specimens were subjected to overnight incubation at 4 ℃ with antibodies targeting TH, GPX4, ACSL4, TF, PTGS2, Nrf2, HMOX1, FTH, and GAPDH. Afterward, the membrane and the secondary antibody were placed in a controlled environment at room temperature for a duration of 1 h. Finally, 1 × TBST was used to wash the membrane, and an enhanced chemiluminescence (ECL) detection reagent was used to show the protein bands. Data were analyzed using Gel-Proanalyzer4.0 software (GelMediaSystem, China).

### Statistical analysis

SPSS statistical software was used for statistical analysis. The measured values are expressed as Mean ± SD A one-way analysis of variance (ANOVA) was employed to compare the groups, and a paired comparison was performed using the least significant difference (LSD) and Dunnett test. P < 0.05 manifests a significant difference.

## Results

### Target prediction of potential compounds of CDG against PD

**Fig. 1:**
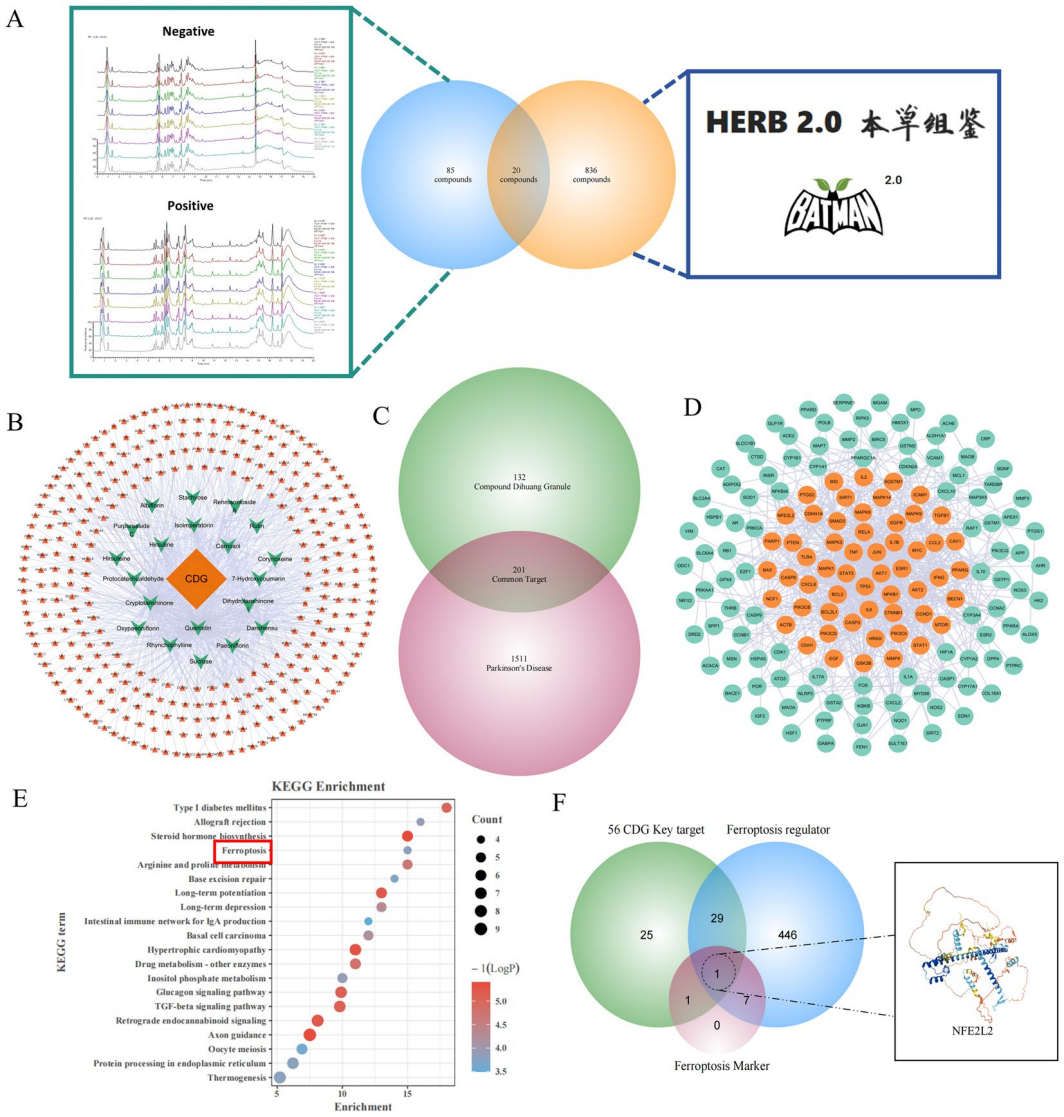
Identification of CDG compounds by LC–MS and database. **A** 105 main compounds from CDG were identified by LC–MS; 856 compounds contained in CDG were collected from databases; 20 compounds were finally screened as the main compounds of CDG. **B** Network diagram of 20 compounds and potential targets. Green represents 20 important compounds of CDG, and the orange circle on the outer layer represents the potential targets of the compounds. **C** Venn diagram of 201 common targets of CDG and PD. **D** Acquirement of the 56 key targets from the 201 common targets via CytoNCA. **E** 201 common targets for KEGG enrichment analysis. **F** NFE2L2 was selected as the core gene through intersection with key targets of CDG, genes involved in ferroptosis regulation, and ferroptosis markers

A total of 856 chemical compounds in CDG were gathered from the TCM database (S2). Following the intersection with LC–MS outcomes (S1), 20 compounds were identified, comprising Rhynchophylline, Paeoniflorin, Rutin, Danshensu, Rehmannioside A, Carnosol, Purpureaside C, Hirsuteine, Isoimperatorin, 7-Hydroxycoumarin, Hirsutine, Stachyose, Quercetin, Albiflorin, Cryptotanshinone, Protocatechualdehyde, Oxypaeoniflorin, Sucrose, Corynoxeine, and Dihydrotanshinone I (Fig. 1A). Then, 333 targets were predicted and filtered for these 20 compounds (Fig. 1B, S3). Furthermore, 1,712 potential therapeutic targets for PD were retrieved from GeneCards, DrugBank, CTD, DisGeNET, PharmGKB, and OMIM databases. Ultimately, 201 shared targets were selected as candidate targets for CDG in PD treatment (Fig. 1C, S4).

To assess the significance of the 201 candidate targets, they were submitted to the STRING 12.0 database for analysis. The resulting data was subsequently used to construct a Protein–Protein Interaction (PPI) network in Cytoscape 3.10.2, yielding a network comprised of 158 nodes interconnected by 740 edges, with an average node degree of 9.367. Targets exhibiting topological parameters (Eigenvector, Betweenness, Closeness) surpassing the median threshold were designated as key targets, leading to the identification of 56 such targets (Fig. 1D, Table S5). Notably, three of these key targets—NFE2L2, PTGS2, and HMOX1—are recognized as pivotal genes associated with ferroptosis.

To elucidate the potential roles of shared targets in CDG and PD, KEGG pathway enrichment was conducted via the Metascape platform (https://metascape.org). Pathways found to be enriched included Type I diabetes, Allograft rejection, Steroid hormone biosynthesis, Ferroptosis, Arginine/proline metabolism, Base excision repair, Long-term potentiation/depression, and Intestinal IgA production (Fig. 1E). Of note, ferroptosis was a recurrent focus, which attracted our attention. Given the extensive role of ferroptosis in PD pathogenesis has been widely proposed, our focus centered on the intersection of three crucial sets: the 56 key targets, genes involved in regulating ferroptosis, and ferroptosis markers, the latter two were derived from the FerrDB database (http://www.zhounan.org/ferrdb/). We finally got a core gene NFE2L2 (Nrf2) for CDG treatment of PD ferroptosis (Fig. 1F, S6, S7).

### CDG attenuates motor deficits and the degeneration of DA neurons in 6-OHDA-induced PD rats

**Fig. 2:**
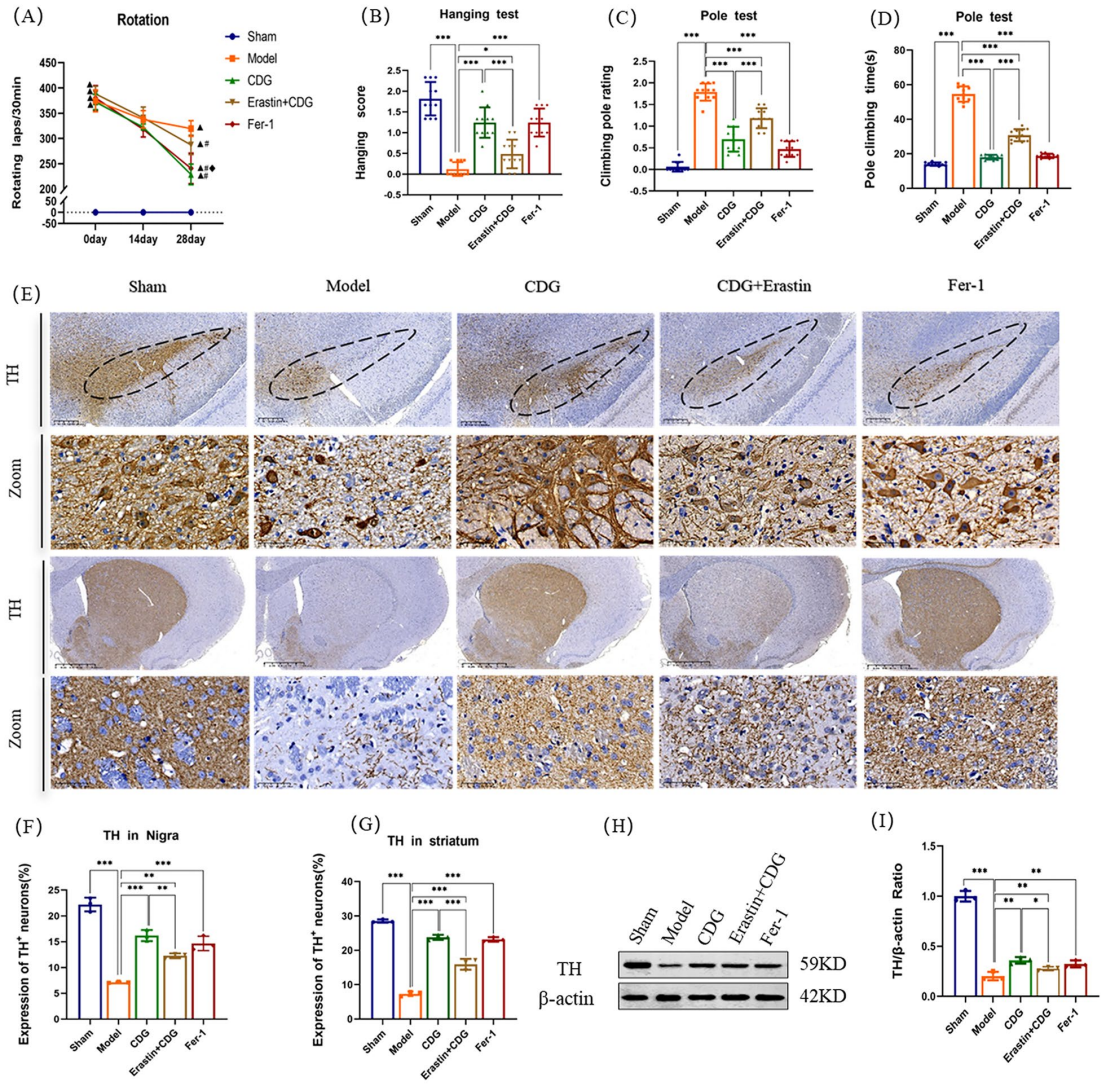
CDG reduces motor impairment and neuronal damage in PD rats induced by 6-OHDA. **A** Assessment of rotational behavior, indicating significant differences: ▲ vs. Sham group, P < 0.01; # vs. Model group, p < 0.01; ◆ vs. CDG group, P < 0.01. **B** Score from the hanging test. **C, D** Aggregate duration and performance score in the climbing pole test, with n = 11 rats per group. **E** TH-positive neuron immunostaining in the SNpc across all groups. **F, G** Quantitative immunohistochemical analysis of TH-positive cells, data shown as mean ± SD (**H, I**) Western blot quantification of TH protein levels in the striatum, analyzed by one- or two-way ANOVA, n = 3. Significance denoted by *p < 0.05; **p < 0.01; ***p < 0.001

To investigate whether CDG treatment can rescue DA neurons from 6-OHDA-induced PD rats, neurobehavioral tests and TH expression in the striatum and substantia nigra were performed. We measured the rotational behavior of APO-induced rats at 0, 14, and 28 days after administration with CDG (Fig. 2A). The results indicated that the rats exhibited significant side-biased rotational movements in Model group, and the rats in the Sham group had no rotation after apomorphine injections. The CDG, the Erastin + CDG and the Fer-1 group rats possessed a significant hindrance at 28 days in the number of rotation circles, comparing to the Model group. In addition, the Erastin + CDG group significantly mitigated the circles of rotation, when compared to the CDG group.

To further demonstrate the protective effects of CDG on restoring motor functions in PD rats. Hang tests and pole tests were conducted to estimate the motor function of PD rats. Results for the hang test are shown in Fig. 2B. The scores of turn around were significantly raised in the rats of Model group when compared to the rats of the Sham group. After the continuous intervention of CDG for 4 weeks, the hang scores were significantly reduced in both CDG and Fer-1 groups of rats compared with the Model group. Similarly, significant changes were observed between the Erastin + CDG and CDG groups in the hang scores. Next, the pole test results **(**Fig. 2C, D) showed that the climbing pole rating and pole climbing times of the rats in Model group were significantly ascended to the Sham group. CDG and Fer-1 groups of rats showed a significant decrease in the climbing pole rating and pole climbing times, indicating visible protective effects of CDG compared to that of the Model group. The Erastin + CDG group had similar effects on the pole test, and there was an obvious upward trend compared with the CDG group.

Tyrosine hydroxylase (TH) is the enzyme that controls the speed at which dopamine is produced, and it is an important indicator for dopaminergic neurons [29]. We detected TH-positive cells in the SNpc via immunohistochemical staining. TH staining revealed large amounts of brown or tan TH-positive cells with clear reticular structure and full shape in the SNpc of the Sham group. 6-OHDA is a neurotoxicant and is often used to create models of PD, both in vivo and in vitro [30]. We observed that the expressions of dopaminergic neurons in SNpc of the rats in Model group was prominently decreased, cell fragmentation and the cell outline was vague. CDG and Fer-1 treatment could partially rescue this polarization (Fig. 2E). Similarly, CDG and Fer-1 significantly increased the striatal TH fiber density to the rats in Model group. Based on a Western Blot analysis, the expression of TH in the CDG group enhanced significantly to the Model group. The result is shown in Fig. 2H, I. Similar results of the increase of TH expression were observed in the Fer-1 and Erastin + CDG groups. Western blot analysis further confirmed these immunohistochemical staining results.

### CDG inhibits 6-OHDA-induced ferroptosis in DA neurons

**Fig. 3:**
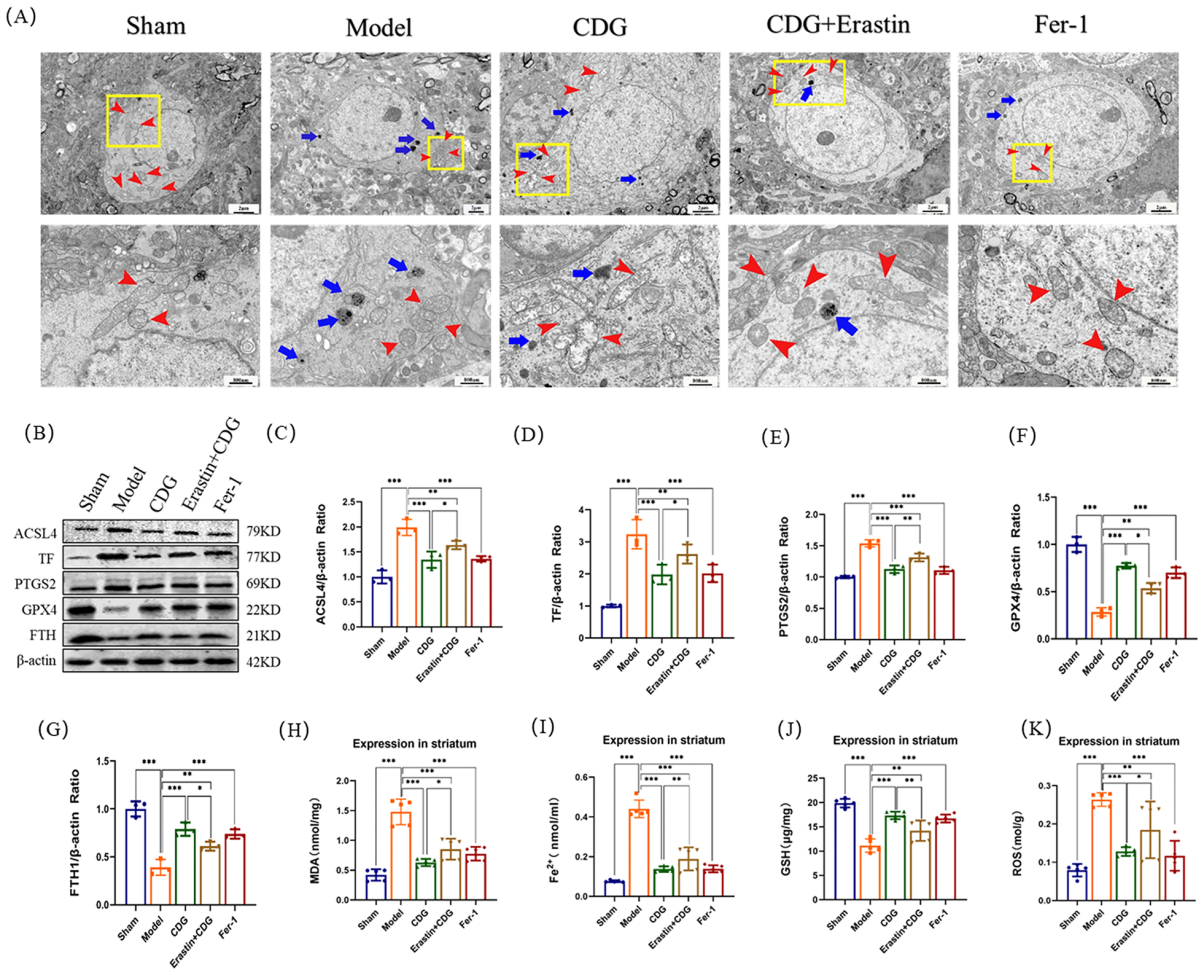
CDG Mitigates Ferroptosis in Dopaminergic Neurons Following 6-OHDA Exposure. **A** Electron microscopy images of the rat striatum, highlighting mitochondria (red arrow) and chromatin (blue arrow). **B–G** Western blot analysis of ferroptosis markers including ACSL4, TF, PTGS2, GPX4, and FTH in the striatum; n = 3. **H–K** Serum antioxidant and oxidative stress markers including MDA, Fe^2+^, GSH, ROS, and SOD; n = 5. Data analysis employed one-way ANOVA and Turkey's multiple comparisons

Known as ferroptosis, it is a process of cell death that is iron-dependent, one of the typical features of which is significant ultrastructural changes in mitochondria [31]. TEM observed the mitochondrial morphology of each group. The outcomes manifested that 6-OHDA induced mitochondria of DA neurons atrophy, membrane density increased and ridge decreased or disappeared. However, the treatment of the CDG and Fer-1 partially improved the abnormal mitochondrial morphology of DA neurons. Mitochondrial morphology improved in the CDG + Erastin group, but not as much as in the CDG group (Fig. 3A).

To further demonstrate the CDG function in 6-OHDA-caused ferroptosis in DA neurons, ACSL4, PTGS2, GPX4, TF and FTH proteins were detected by WB (Fig. 3B–K).The expression levels of ACSL4, TF, and PTGS2 protein in the rats of Model group were significantly enhanced, and the levels of GPX4 and FTH were weakened, when contrasted with the Sham group, while the treatment effects were reversed in CDG group, Fer-1 group, and CDG + Erastin group. We also found that CDG can reduce the levels of MDA, ROS, and Fe^2+^ of PD rats caused by 6-OHDA and increase the expression level of GSH (Fig. 3H–K). In conclusion, we demonstrated that CDG can significantly elevate the morphology of ferroptosis mitochondria and the expression of related proteins.

### Transcriptomic characteristics of striatal gene expression in PD rats treated with CDG were analyzed by RNA sequencing

**Fig. 4:**
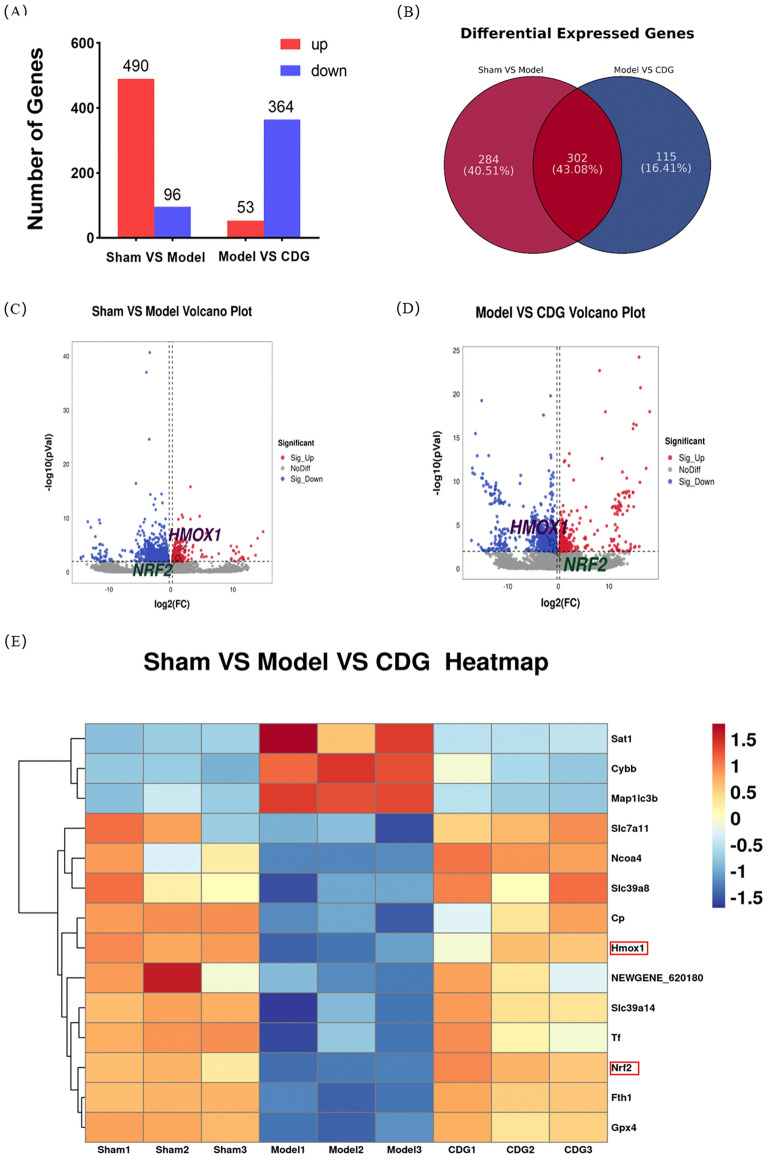
Transcriptomic Insights into PD: Differential Gene Expression in Rat Striatum. **A** Count of genes upregulated and downregulated across groups. **B** Venn diagram showing overlap of differentially expressed genes among groups. **C, D** Volcano plots of differential gene expression. **E** Heatmaps of differential expression across Sham, Model, and CDG groups, with red indicating higher expression and blue lower expression (log|FC|> 1.2, P < 0.01)

To elucidate the underlying mechanisms of CDG, we performed RNA-Seq analysis on rat striatal tissue 28 days post-CDG administration. We found that the Sham group had 586 differential genes, with 490 were upregulated and 96 genes were down-regulated compared with the Model group (Fig. 4A). Contrasted with the CDG group, the Model group had 417 heterogenes, with 53 genes were upregulated and 364 genes were down-regulated (log|FC|> 1.2, p ≤ 0.01). There were 302 common differential genes in the two comparisons, and Nrf2 and HMOX1 were enriched in the differential genes in both comparisons (Fig. 4B–E). Specific differential genes are listed as S8, S9, and S10.

**Fig. 5:**
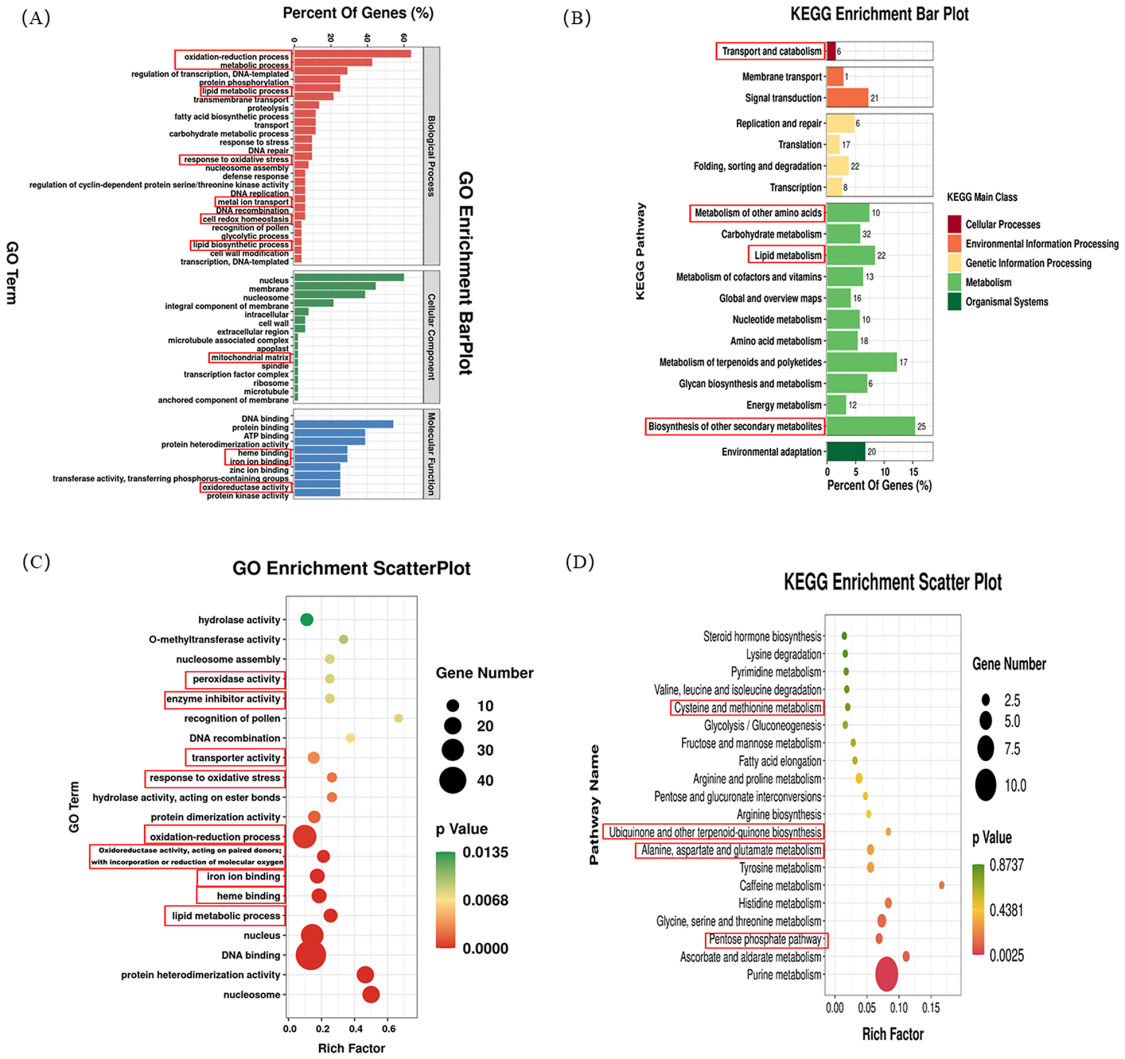
Comparative transcriptomic analysis: functional annotations and pathway enrichment. **A, B** Gene Ontology (GO) and Kyoto Encyclopedia of Genes and Genomes (KEGG) functional annotations. **C, D** GO and KEGG pathway enrichment analyses

Functional annotation analysis refers to the annotation of the gene ontology (GO) and the KEGG of the target gene to look for the function of differential expression. We further analyzed 302 common differential genes for the above two comparisons and surprisingly found that among the results of GO annotation analysis of common differential genes, ferroptosis was related to oxidation–reduction, metabolic, lipid metabolic processes, response to oxidative stress, cell redox homeostasis, lipid biosynthetic process, mitochondrial matrix, heme binding, iron ion binding, and oxidoreductase activity (Fig. 5A). Ferroptosis was associated with transport and catabolism, metabolism of another amino acid, lipid metabolism, and biosynthesis of other secondary metabolites (Fig. 5B). Functional enrichment analysis involves analyzing the enrichment of selected differential gene sets. The hypergeometric distribution algorithm is used to identify significantly enriched functions of the genes in these gene sets, as well as the major metabolic pathways they are involved in. This includes conducting GO and KEGG enrichment analysis. The results of GO functional enrichment include response to oxidative stress, peroxidase activity, oxidation–reduction process, enzyme inhibitor activity, oxidoreductase activity, reduction of molecular oxygen iron, iron ion binding, heme binding, lipid metabolic process (Fig. 5C). The functional enrichment results of KEGG were cysteine and methionine metabolism, ubiquinone and another terpenoid-quinone biosynthesis, metabolism of alanine, aspartate, and glutamate, and pentose phosphate pathway (Fig. 5D). In conclusion, these results support the participation of 6-OHDA-induced PD rats in ferroptosis process, and the preventive implications of CDG is connected with the suppression of ferroptosis in vivo. The neuroprotective impact of CDG is linked to the suppression of the Nrf2/HMOX1 pathway associated with ferroptosis.

### CDG alleviates 6-OHDA-induced ferroptosis in PD rats by regulating Nrf2/HMOX1 pathway

To manifest the contribution of the Nrf2/HMOX1 pathway in PD-linked ferroptosis, we employed Oltipraz (Nrf2 agonist), ML385 (Nrf2 inhibitor), Hemin (HMOX1 agonist) and ZNPP (HMOX1 inhibitor) as positive controls, respectively, which were used as positive control.

**Fig. 6:**
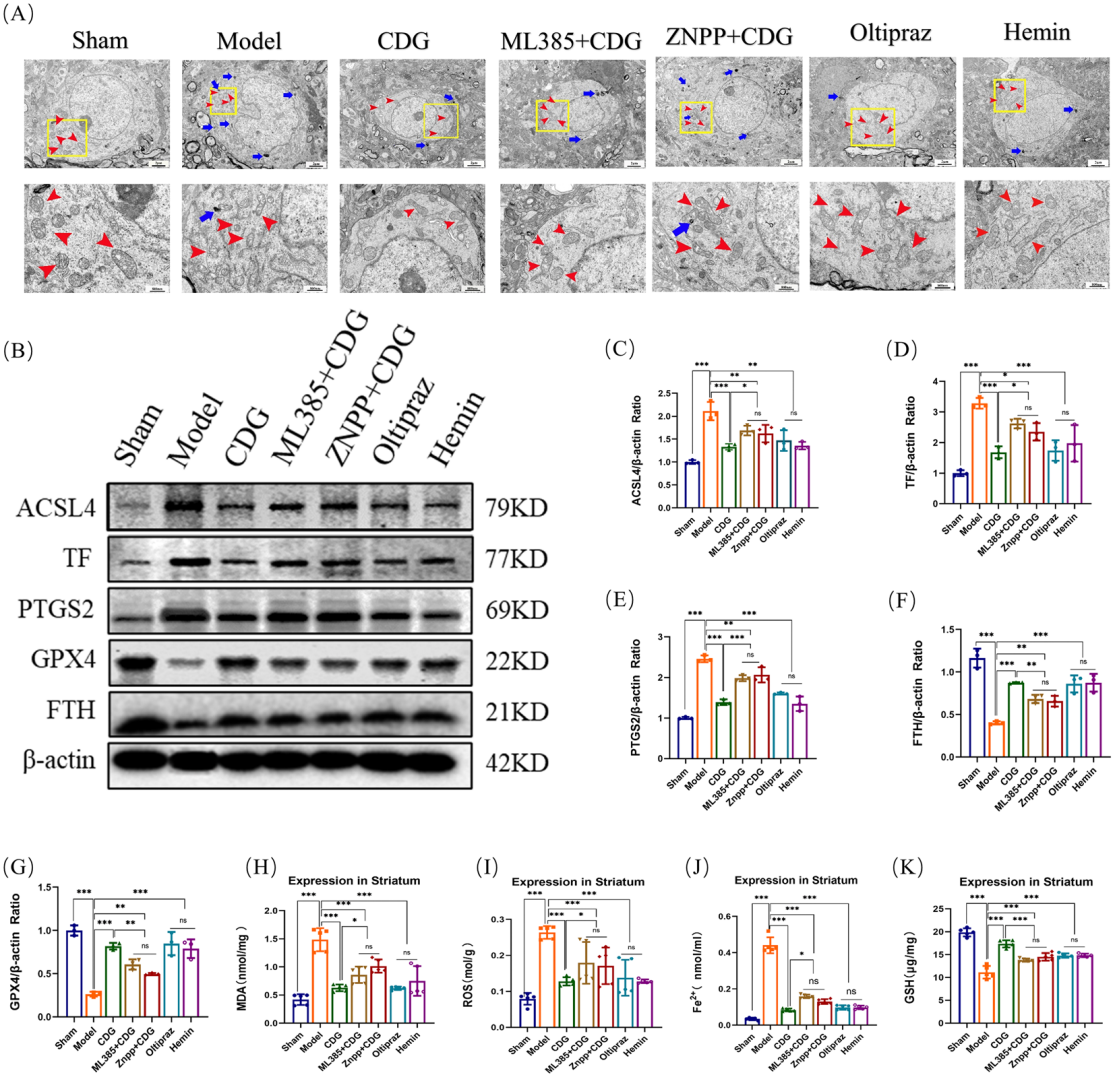
Effectiveness of Nrf2/HOMX1 modulators and CDG against ferroptosis in rat models.** A** Electron microscopy highlighting cellular organelles in the striatum of rats treated with inhibitors and agonists. **B–G** Expression analysis of ferroptosis-related proteins via Western blot; n = 3. **H–K** Serum biochemical markers of oxidative stress and antioxidant defence; n = 5. Statistical analyses include one-way ANOVA and post hoc tests

First, we used TEM to observe whether Nrf2/HMOX1 pathway activators and inhibitors affected mitochondrial damage of DA neurons of PD rats (Fig. 6A). After the administration of Oltipraz, Hemin, and CDG groups, the damage degree of mitochondria was significantly less than the Model group, and the mitochondrial ridge was clearer and folds increased. The degree of mitochondrial damage in ML385 + CDG and ZNPP + CDG groups was lighter than the PD Model group and heavier than the CDG group rats. The mitochondrial membrane can be observed to be damaged, and the mitochondrial crest folds are less. Then, WB assay manifested that the expression of ACSL4, TF and PTGS2 proteins in ML385 + CDG group, ZNPP + CDG group, Oltipraz group, Hemin group and CDG group significantly decreased compared with PD group, while the GPX4 and FTH proteins levels were significantly elevated. Although the protein expression results of the ML385 + CDG group and ZNPP + CDG group were consistent with those of the CDG group, the results of CDG group were little better than the ML385 + CDG group and ZNPP + CDG group (Fig. 6B–G). Then, we also found that MDA, ROS, and Fe^2+^ in CDG group, ML385 + CDG group, ZNPP + CDG group, Oltipraz group, and Hemin group rats were significantly reduced versus the Model group, and the GSH expression level was notably elevated. Contrasted with the ML385 + CDG group and ZNPP + CDG group, the therapeutic effect of the CDG group was more significant (Fig. 6H–K).

**Fig. 7:**
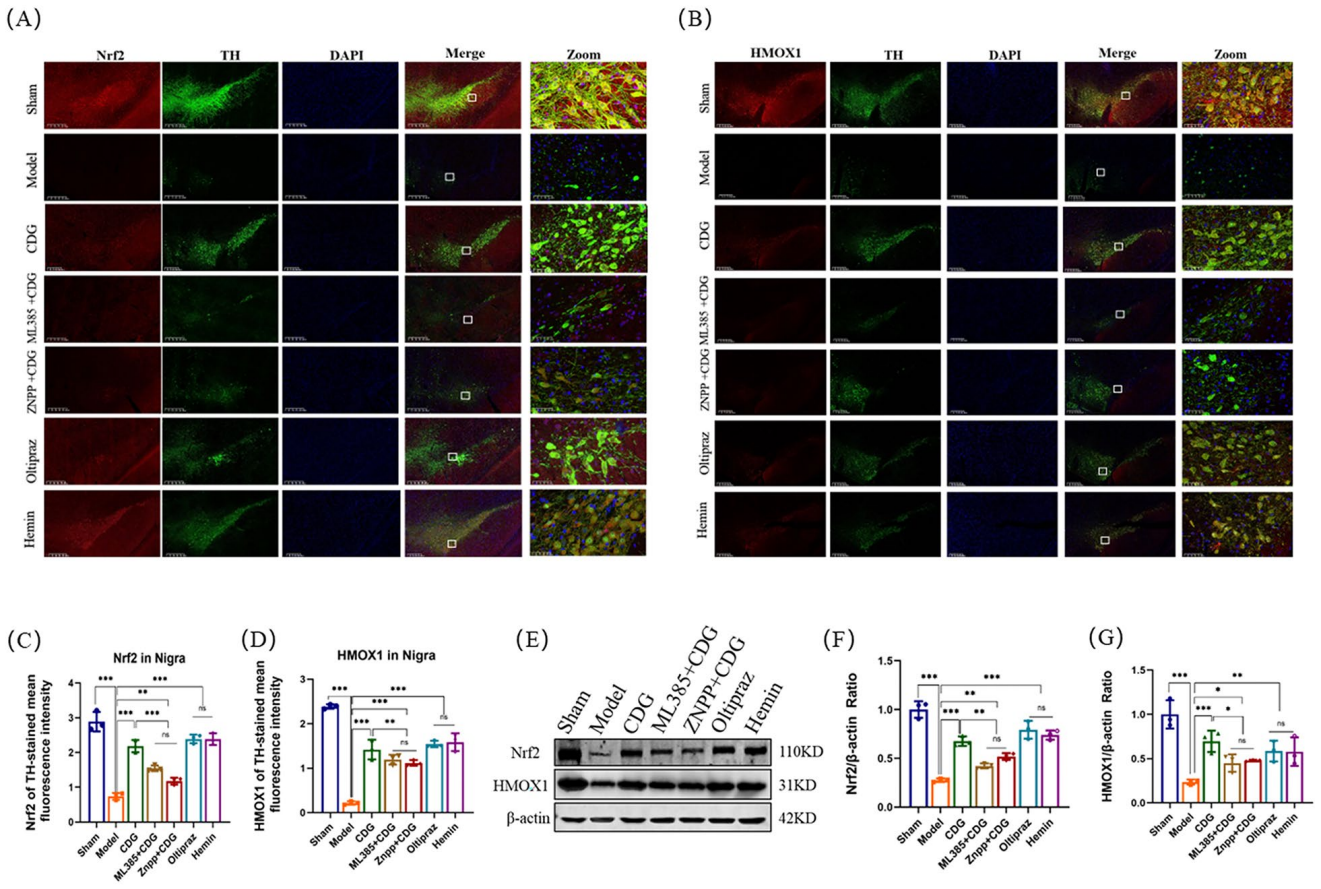
Activation of Nrf2/HOMX1 Pathway by CDG Reduces Ferroptosis. **A**–**D** Levels of Nrf2 and HOMX-1 proteins in the striatum assessed by Western blot. **E**–**G** Quantification of Nrf2 and HOMX-1 expression in the striatum; n = 3. Statistical significance determined by one-way ANOVA and multiple comparison tests

Thereafter, Nrf2 and HMOX1 expression was assessed by immunofluorescence combined staining with TH and WB assay (Fig. 7A–G). The immunofluorescence detection showed that the Nrf2 and HMOX1 expression levels in CDG group, ML385 + CDG group, ZNPP + CDG group, Oltipraz group, and Hemin group rats exhibited a considerable increase contrasted with Model group rats. The therapeutic efficacy of the CDG group was superior to that of the ML385 + CDG and the ZNPP + CDG groups. The Western blot analysis showed similar results. The above data prove that CDG inhibits ferroptosis, which is related to the Nrf2/HMOX1 pathway.

### CDG inhibits ferroptosis of SH-SY5Y cells induced by MPP^+^

**Fig. 8:**
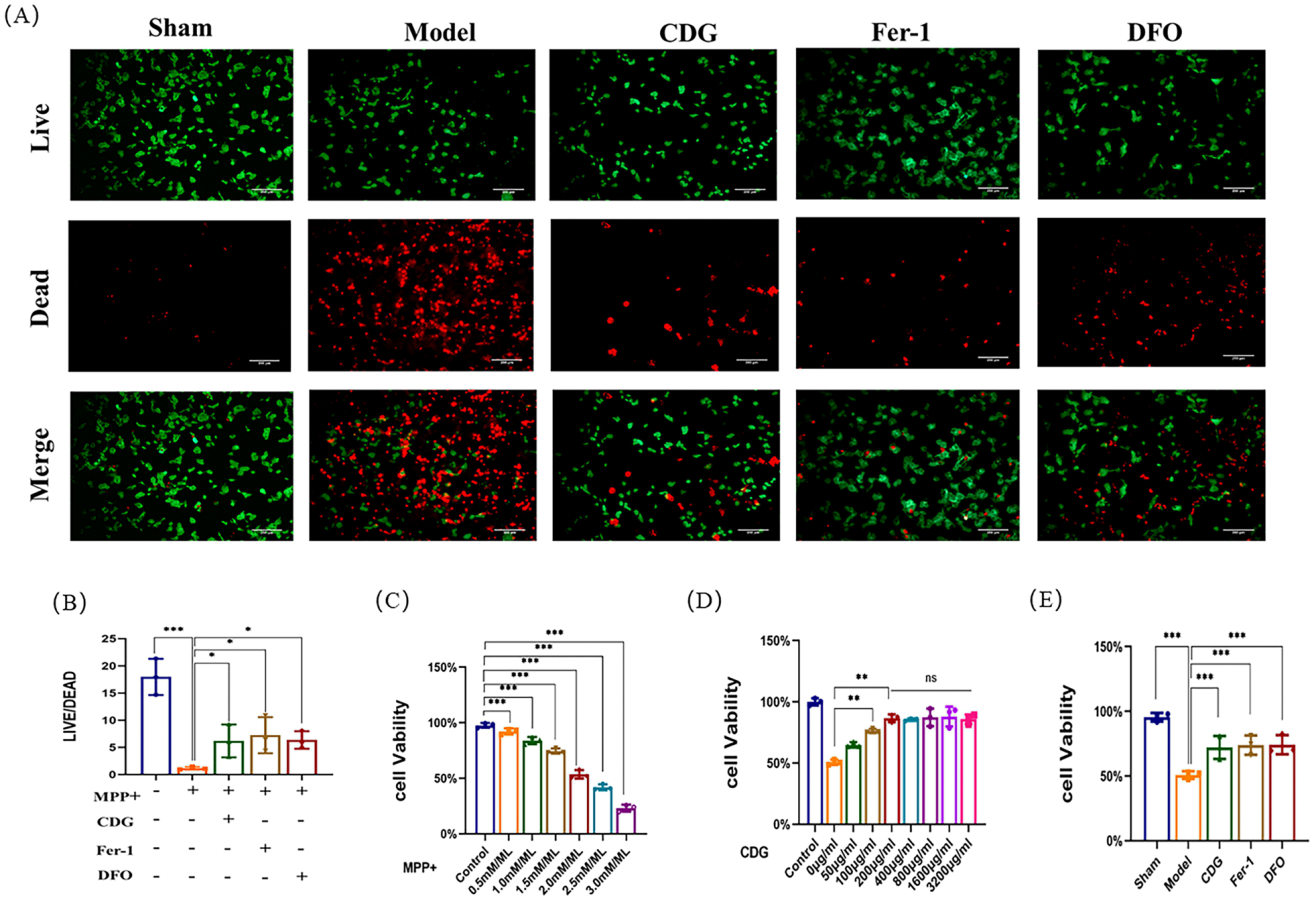
CDG Enhances Cellular Resilience to MPP.^+^ Induced Toxicity in SH-SY5Y Cells. **A**, **B **Live/dead staining images. **C, D** Cell viability assays post MPP^+^ and various treatments quantified using the CCK8 assay; n = 6. **E** Comparative cell viability results. Analyses of variance are performed using one-way ANOVA, Tukey's test.

In order to further confirm that CDG can play a role in inhibiting ferroptosis, we conducted in vitro experiments. First, the PD model of SH-SY5Y cells was constructed. Different concentrations of MPP^+^ (0.5, 1.0, 1.5, 2.0, 2.5, and 3.0 mM) were administered to SH-SY5Y cells. It was found that SH-SY5Y cells were damaged dose-dependent at all concentrations of MPP^+^ except 0.5 mM (Fig. 8C). Based on the maximal suppressive concentration measurement, a concentration of 2 mM was chosen for future experimentation. Subsequently, CCK-8 experiment results showed that the 200 μg/mL CDG might mitigate the detrimental effects of MPP^+^ on SH-SY5Y cells. So we chose 200 μg/mL of CDG for subsequent experiments (Fig. 8D). We performed live/dead tests and CCK-8, respectively, to assess cell viability. Green and red represent living and dead cells, respectively, and the images showed that the Model group had more dead cells than the Sham group. Compared with the Model group, the cell viability of the CDG group, Fer-1 group, and DFO group was correspondingly increased (Fig. 8A, B, E). The CCK-8 experiment yielded similar results.

**Fig. 9:**
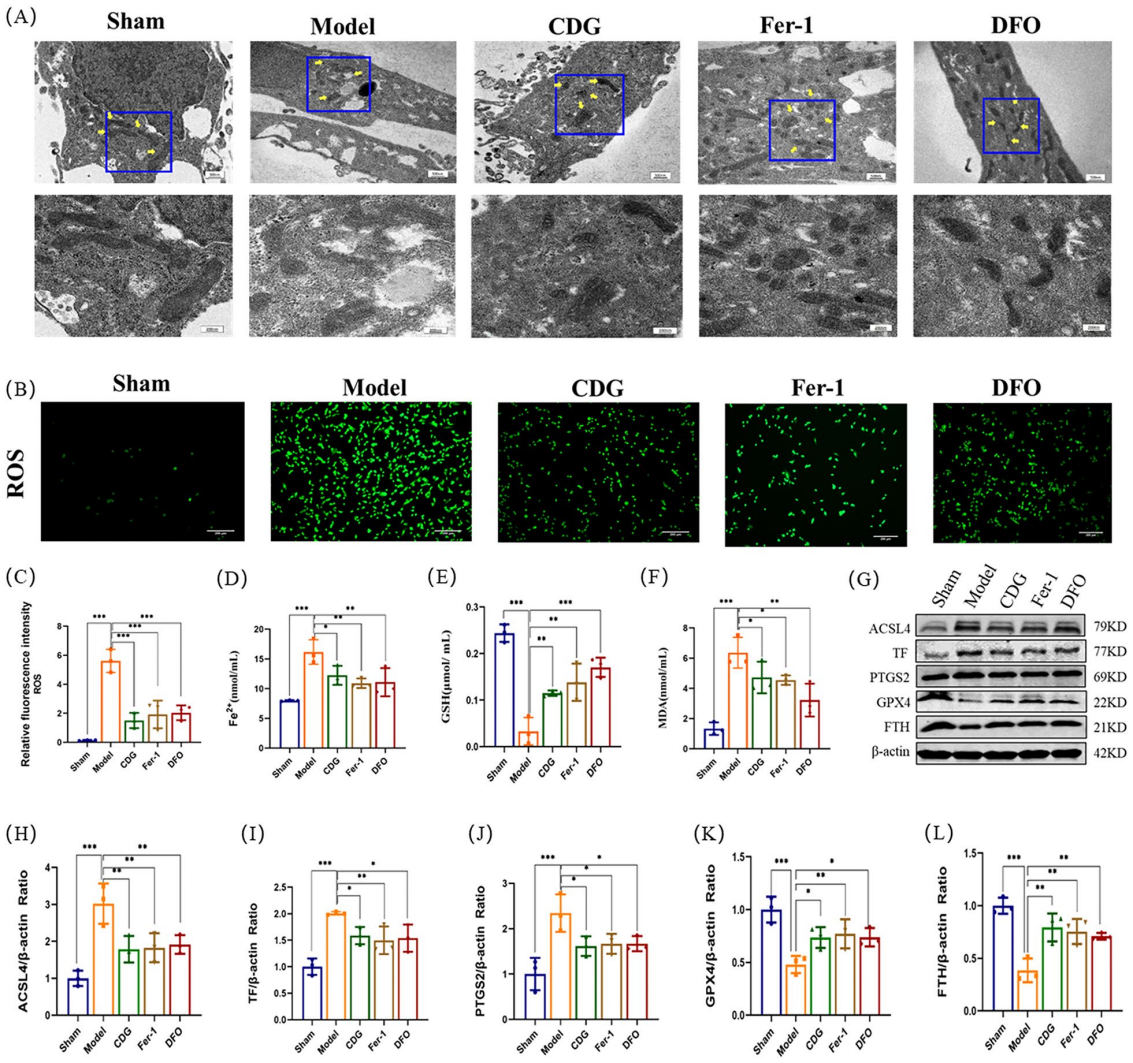
Role of CDG and iron chelators in mitigating ferroptosis in SH-SY5Y cell lines.** A** Electron microscopy images showing cellular ultrastructure. **B–L** Analysis of intracellular markers of ferroptosis and antioxidant defenced, including protein expression studies using Western blot; n = 3. Statistical analysis using one-way ANOVA and multiple comparisons

MPP^+^, as a neurotoxin, can cause cells to produce and accumulate a large amount of ROS. Firstly, TEM observed the morphology of mitochondria in the Sham group. The mitochondria were round or oval, and the mitochondrial membrane and ridge were clear and complete. Mitochondrial atrophy, mitochondrial membrane damage, and mitochondrial ridge reduction were observed in the Model group. Following the intervention of the CDG group, Fer-1 group, and DFO group, the mitochondrial shape was slightly wrinkled and roughly elliptical, the mitochondrial membrane was relatively complete, and the mitochondrial ridge structure was clear (Fig. 9A). ROS and MDA levels serve as essential indications for cellular oxidative damage, whereas Fe^2+^ and GSH levels serve as vital indications for cell ferroptosis. Under the intervention of MPP^+^, ROS, MDA, and Fe^2+^ levels of SH-SY5Y cells in the Model group were significantly increased, while GSH activity was significantly decreased. After the intervention of the CDG group, Fer-1 group, and DFO group, the expressions of ROS, MDA and Fe^2+^ were reduced and the level of GSH was increased significantly (Fig. 9B–F). Subsequently, Western Blotting was employed to confirm the ferroptosis-related molecules. The outcomes that contrasted with the Sham group were that the Model group showed a significant elevation in ACSL4, TF, and PTGS2 protein expression levels and a decline in GPX4 and FTH protein expression levels. However, the intervention of the CDG group, Fer-1 group, and DFO group reversed these impacts in the Model group (Fig. 9G–L).

### CDG prevented SH-SY5Y cells ferroptosis though activating the Nrf2/HMOX1 pathway

**Fig. 10:**
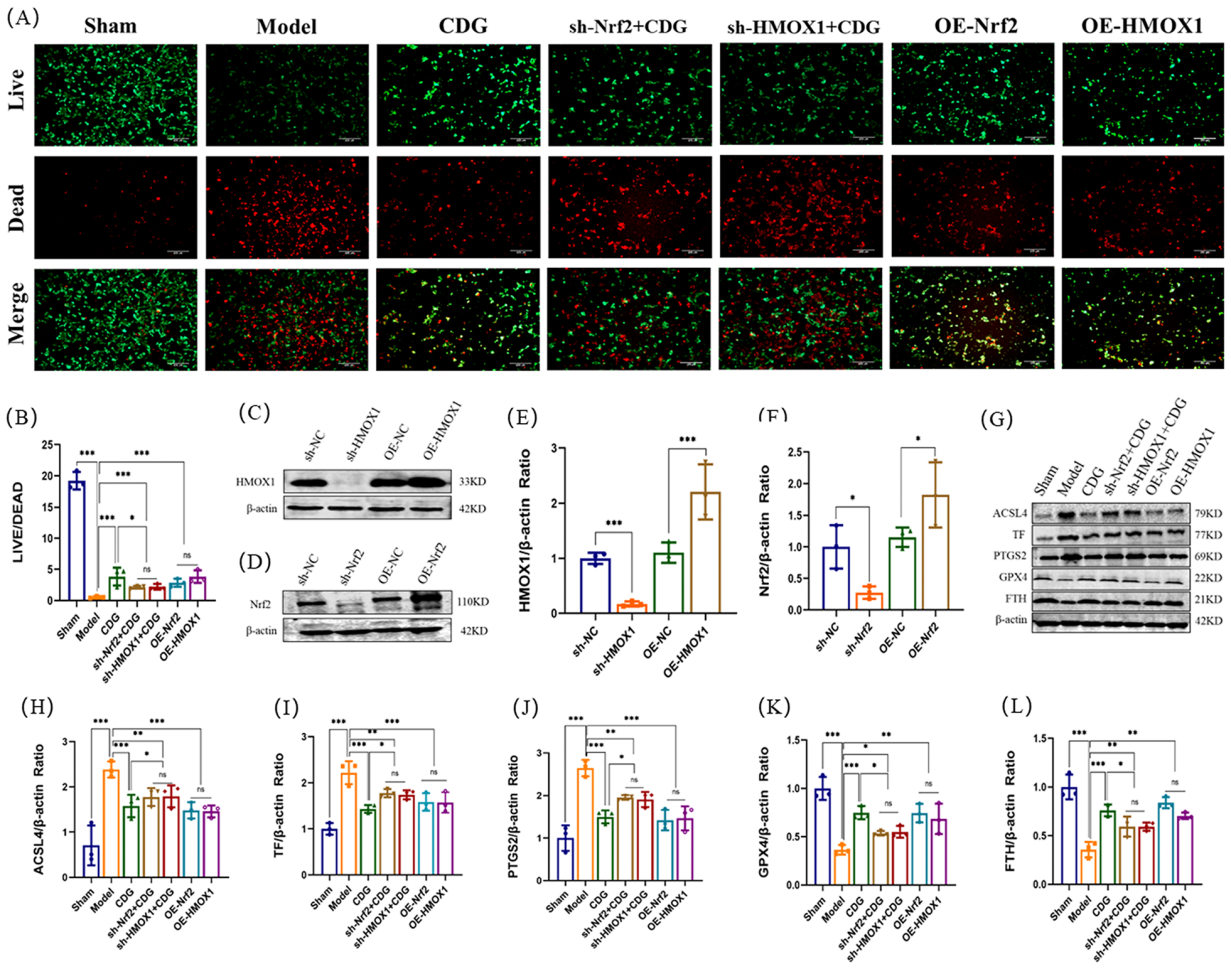
Inhibition of ferroptosis by Nrf2/HOMX1 modulation in SH-SY5Y cell lines. **A**, **B** Visualization of cell viability through live/dead staining. C-F Quantification of protein levels via Western blot post-transfection. **G–L** Detailed protein expression profiles related to ferroptosis analysed using Western blot; n = 3. Statistical tests include one-way ANOVA and post hoc analysis

**Fig. 11:**
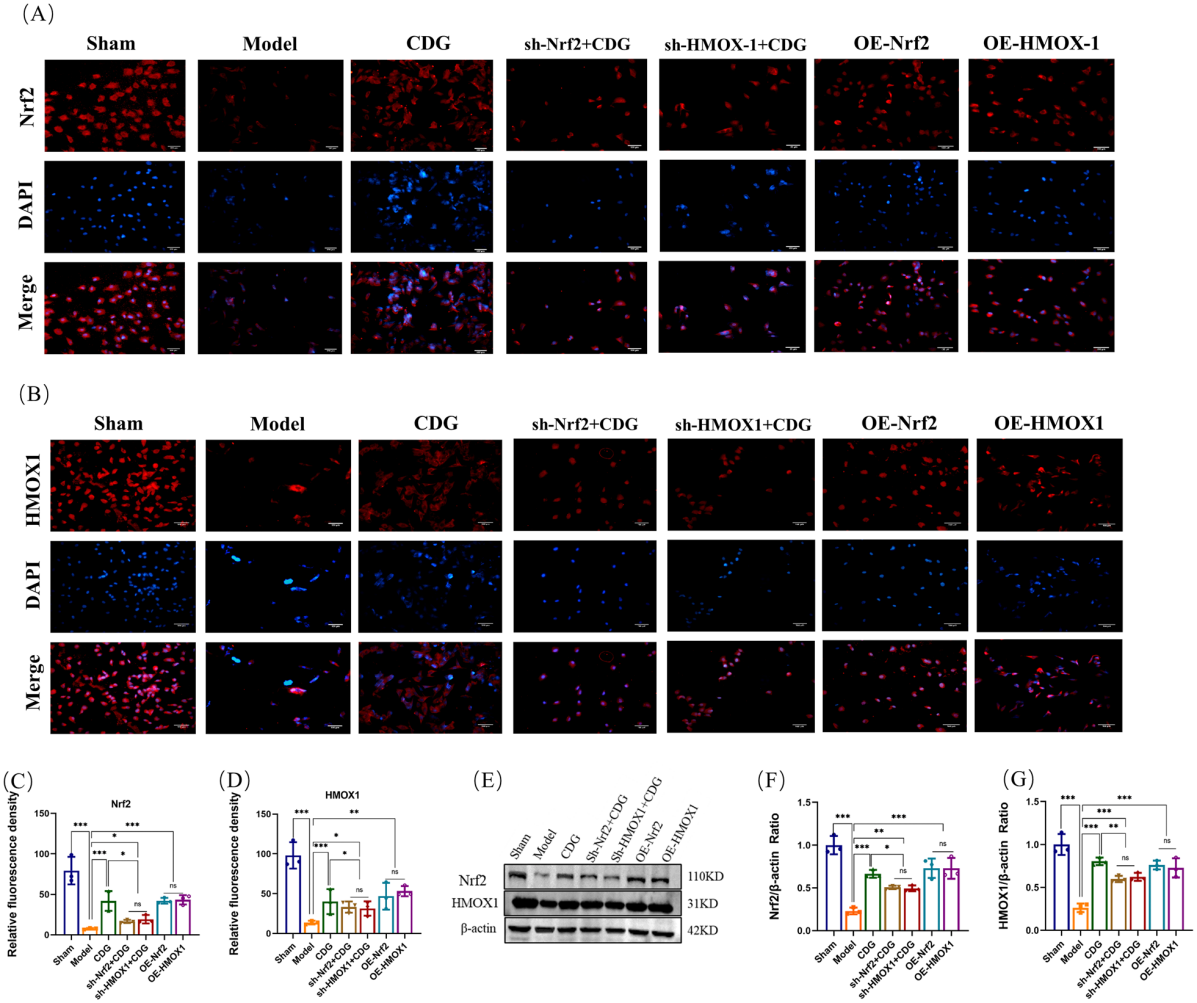
Nrf2/HOMX1 pathway activation by CDG prevents ferroptosis in SH-SY5Y cell lines.** A–D** Immunofluorescence analysis of Nrf2 and HOMX-1 in treated SH-SY5Y cells. **E–G** Western blot analysis quantifying Nrf2 and HOMX-1; n = 3. Statistical significance evaluated using one-way ANOVA and Turkey's test

To fully demonstrate the activation effect of CDG on the Nrf2/HMOX1 pathway, lentiviral induction was performed on SH-SY5Y cells, and sh-Nrf2, sh-HMOX1, OE-Nrf2, and OE-HMOX1 cell lines were constructed, respectively. After the construction was completed, Western blotting was first used for verification (Fig. 10C–F). We assessed cell viability using live/dead tests, with green and red representing live and dead cells, respectively. The imaging findings indicated a significant rise in the number of deceased cells in the Model group compared to the Sham group. The dead cells of the CDG group, OE-Nrf2 group, OE-HMOX1 group, sh-Nrf2 + CDG group, and the sh-HMOX1 + CDG group were less than the Model group, while the dead cells of the sh-Nrf2 + CDG group and the sh-HMOX1 + CDG group were more than those of CDG group. Finally, the ratio of live cells to dead cells was consistent with the trend of the images (Fig. 10A, B). Subsequently, Western blotting results showed that ACSL4, TF, and PTGS2 proteins were significantly enhanced in PD rats, while GPX4 and FTH proteins levels were weakened. CDG Group, OE-Nrf2 group, OE-HMOX1 group, sh-Nrf2 + CDG group, and sh-HMOX1 + CDG group reversed the effect of the Model group. ACSL4, TF, and PTGS2 proteins expression of the sh-Nrf2 + CDG group and the sh-HMOX1 + CDG group were significantly improved, when compared with the CDG group, while the expression levels of GPX4 and FTH proteins were significantly lowered (Fig. 10G–L). Finally, immunofluorescence detection results showed that Nrf2 and HMOX1 in the CDG group, OE-Nrf2 group, OE-HMOX1 group, sh-Nrf2 + CDG group, and sh-HMOX1 + CDG group were improved significantly to the Model group. The expression levels of Nrf2 and HMOX1 in the sh-Nrf2 + CDG group and the sh-HMOX1 + CDG group were significantly lower than those in the CDG group (Fig. 11A–D). Western blot results showed the same trend as above (Fig. 11E–G). These results confirmed that the suppressive impact of CDG on MPP^+^ induced ferroptosis of SH-SY5Y cells was through activating the Nrf2/HMOX1 pathway.

## Discussion

PD is a common neurodegenerative disorder distinguished via a complicated pathophysiology, which poses significant challenges for therapeutic intervention. Due to its multi-component nature, ability to target several factors, and overall regulatory effects on disease therapy, TCM has significant promise in both preventing and treating PD [32]. CDG is a common therapeutic agent in traditional Chinese medicine clinics for PD. Much effort has been made in clarifying the therapeutic mechanism of action of CDG and quality control in our previous work [33, 34]. However, the molecular mechanisms underlying remain less well-defined. The current work investigated the processes and mechanism that cause the effects of CDG on PD through network pharmacology prediction, RNA-Seq data and conducting in the in vitro and in vivo studies.

Unilateral 6-OHDA-lesioned rats are extensively used as an experimental model for PD [35, 36]. APO-induced rotation behavior is a pivotal quantitative assessment method for PD models [37]. Previous studies have suggested TH is the enzyme that controls the rate of dopamine production and is often employed as an indicator for dopaminergic neurons [38]. Moreover, in this investigation, we measured the rotational behavior of APO-induced rats; meanwhile, the hang test and pole test were employed to evaluate the motor function of PD rats. We also detected TH-positive cells in the SNpc via IHC and the levels of the TH protein by Western blotting. The experimental results confirmed the Model group rats exhibited significant side biased rotational movements and manifested a PD motor impairment. After 4 weeks of treatment, we demonstrated that CDG could attenuate DA neuronal injury and alleviate behavioral deficiencies induced by 6-OHDA.

Network pharmacology is a powerful methodology to underly the mechanisms of the action of TCM. In the present study, network pharmacology along with LC–MS, 56 key targets were screened out from the 201 targets using PPI network analysis, including NFE2L2, PTGS2 and HMOX1, which are recognized markers of ferroptosis. KEGG results showed higher enrichment included ferroptosis. Meanwhile, NFE2L2 is the only one that the intersection of the 56 key targets, ferroptosis regulatory genes and ferroptosis marker in accordance with the FerrDb database, which suggested that ferroptosis may contributes to the pathogenesis of PD, and Nrf2 was regarded as candidate targets of CDG for treating PD.

Neuropathological and imaging investigations have shown that PD patients and animal models of PD have specific iron deposits in the SN [39]. ROS accumulation, GSH depletion, and lipid peroxidation were confirmed to take part in pathogenesis of PD. ROS-mediated lipid peroxidation elevated MDA levels. Glutathione peroxidase 4 (GPX4) plays an essential involvement in ferroptosis [40]. and ACSL4 has been regarded as a biomarker of ferroptosis [41]. Transferrin (TF) has been shown to facilitate oxidative stress and induce ferroptosis through enhanced iron uptake and suppressed iron efflux [42]. Since FTH is an important part of ferritin, decreasing FTH expression can increase the free iron level to promote ferroptosis [43]. Simultaneously, Ptgs2 was induced in cells undergoing ferroptosis [44]. In our experiment, we found that CDG decreased the level of Fe^2+^, reduced the lipid ROS and MDA levels, and elevated the GSH levels. TEM helped to estimate the morphological features of 6-OHDA-induced cell death in rat SNpc. Results suggested that after CDG interventions, mitochondrial morphology improved, and the mitochondrial cristates tended to be discernible. In parallel, we ascertained the protein expression of ACSL4, TF, PTGS2, GPX4, and FTH involved in the ferroptosis. The WB assay revealed that CDG declined the ACSL4, TF, and PTGS2 levels, while increasing the expressions of GPX4 and FTH. Collectively, we demonstrated that CDG might exert a neuroprotective effect by inhibiting 6-OHDA-induced ferroptosis of the neuronal cells.

With the advancement of RNA sequencing technology, a foundation has been laid for further elucidating the mechanism of action of TCM. In this work, we exerted RNA-seq analysis and the outcomes revealed that Nrf2 and HMOX1 were down-regulated observed in the CDG group but significantly increased in the rats of Model group. GO analysis of common differential genes, ferroptosis was related to oxidation reduction process, metabolic process, lipid metabolic process, response to oxidative stress, cell redox homeostasis, lipid biosynthetic process, mitochondrial matrix, heme binding, iron ion binding, and oxidoreductase activity. The functional enrichment results of KEGG were cysteine and methionine metabolism, ubiquinone and another terpenoid-quinone biosynthesis, metabolism of alanine, aspartate, and glutamate, and pentose phosphate pathway. Combined with the above results, the involvement of the ferroptosis process in 6-OHDA-induced PD rats and the possible mechanism underlying CDG protection may inhibit ferroptosis by activating Nrf2/ HMOX1 signaling.

Transcription factor Nrf2 serves as a crucial regulator of cellular redox homeostasis [45]. Research has provided evidence indicating that Nrf2 largely controls ferroptosis by directly or indirectly influencing the production and function of GPX4 [46, 47]. HMOX1, a downstream target gene of Nrf2, is a critical mediator of ferroptosis and plays a vital role in scavenging ROS and attenuating oxidative stress [48].

The Nrf2/HMOX1 pathway is an important regulatory pathway involved in ferroptosis [49, 50]. A recent paper confirmed that the Nrf2/HMOX1 pathway mediated ferroptosis in PD [51]. Accordingly, our results are in line with previous research. To further confirm the role of Nrf2/HMOX1-mediated ferroptosis in PD, Nrf2 inhibitor ML385, Nrf2 agonist Oltipraz, and HMOX1 inhibitor ZNPP, HMOX1 agonist Hemin were administrated. We found that Oltipraz and Hemin significantly suppressed the damage degree of mitochondria, decreased the expression of ACSL4, TF, and PTGS2 proteins, and increased the GPX4 and FTH proteins expression. Likewise, the Fe^2+^, lipid ROS and MDA were reduced, and GSH levels were increased after Oltipraz and Hemin were administrated. Additionally, the utilization of immunofluorescence facilitated the analysis of Nrf2 and HMOX1 levels in rat SNpc. Additionally, four weeks of treatment with Oltipraz and Hemin significantly elevated the Nrf2 and HMOX1 expression in TH-stained cells, and the protein of Nrf2 and HMOX1 were both increased in our results. Interestingly, similar results were shown in the ML385 + CDG and ZNPP + CDG group, indicating that CDG treatment reversed the effect of ML385 and ZNPP. In the in vitro experiments, the OE-Nrf2 and OE-HMOX1 treated group increased cell viability decreased the expression of ACSL4, TF, and PTGS2 proteins, and promoted the GPX4 and FTH protein expression; the results were further supported with the immunofluorescence experiments. The impact is largely reversed by sh-Nrf2 + CDG and sh-HMOX1 + CDG. In this study, we demonstrate that CDG alleviates PD motor disorders and alleviates DA neuron damage. And it revealed that the mechanism by which CDG inhibited PD ferroptosis was related to the activation of the Nrf2/HMOX1 pathway.

## Conclusions

**Fig. 12:**
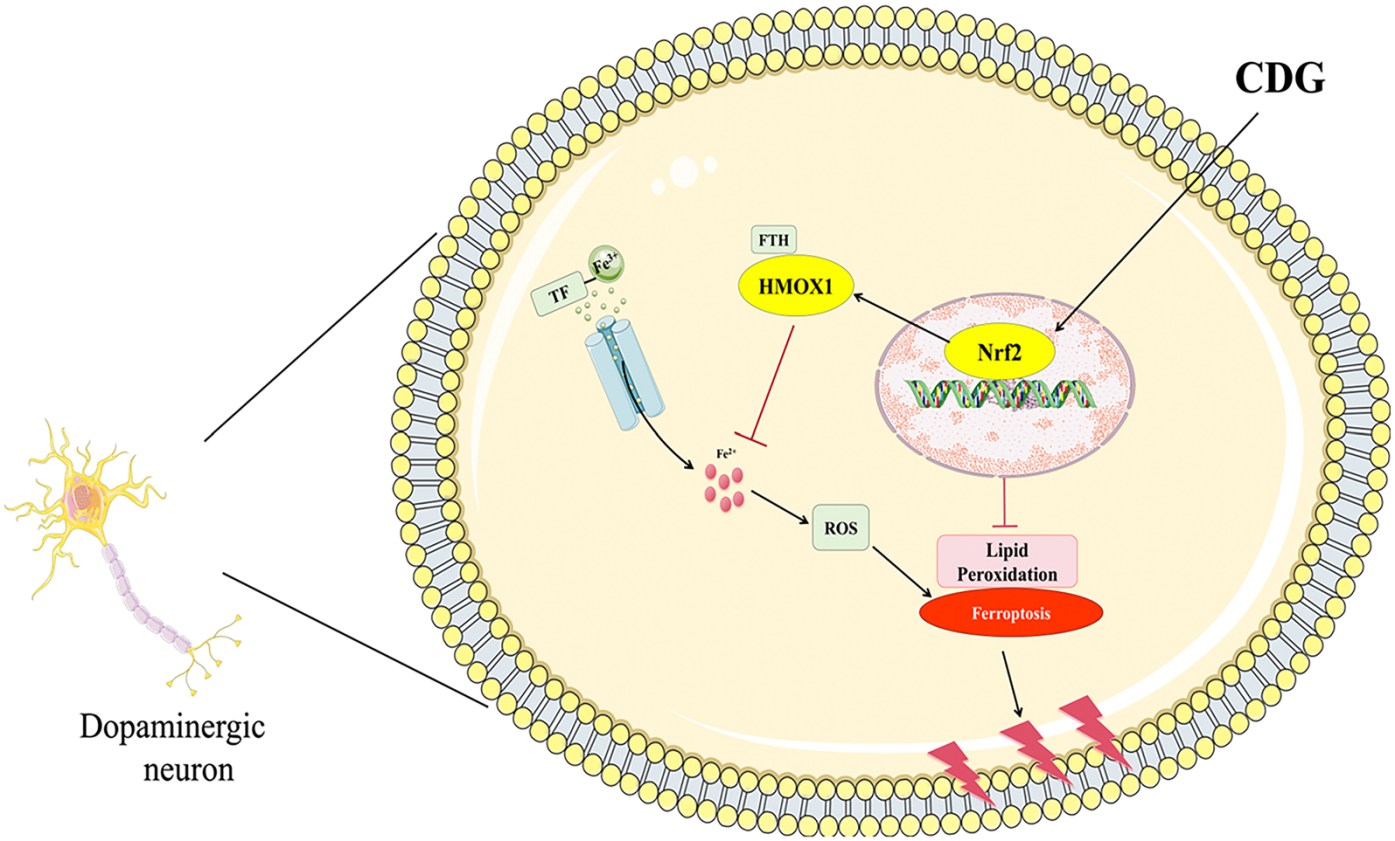
The mechanism of the Parkinson’s disease effects of CDG on dopaminergic neuronal

Our study indicated that CDG alleviates 6-OHDA-induced DA neuronal ferroptosis, as evidenced by alleviating motor deficits and mitochondria damage, protecting DA neurons, and reducing the Fe^2+^ levels, reducing lipid peroxidation. Moreover, network pharmacology, RNA-seq analysis and in vivo and in vitro experiments showed that CDG protected DA neurons from ferroptosis via Nrf2/HMOX1 pathway activation (Fig. 12). The results of our experiment will provide new experimental evidences for the TCM treatment of PD targeting ferroptosis, and, also provide reference for the development of related new drugs and the establishment of treatment methods. In the future, in-depth research will be conducted on the bioactive components, mechanisms, and clinical trials of CDG to gain a more scientific understanding of its treatment for PD.

## Supplementary Information

Supplementary Material 1.

Supplementary Material 2.

Supplementary Material 3.

Supplementary Material 4.

Supplementary Material 5.

Supplementary Material 6.

Supplementary Material 7.

Supplementary Material 8.

Supplementray Material 9.

Supplementary Material 10.

## Data Availability

The datasets supporting this study's conclusions are accessible through the corresponding author upon a reasonable request.

## Abbreviations

6-OHDA: 6-Hydroxydopamine hydrobromide
ANOVA: A one-way analysis of variance
APO: Apomorphine
CDG: Compound Dihuang granule
DA: Dopaminergic
ECL: Enhanced chemiluminescence
GO: Gene ontology
HMOX1: Heme oxygenase-1
IF: Immunofluorescence
LSD: Least significant difference
LC–MS: Liquid chromatography–mass spectrometry
Nrf2: Nuclear transcription-related factor 2
PBS: Phosphate buffer solution
PD: Parkinson’s disease
IHC: Immunohistochemistry
PVDF: Polyvinylidene fluoride
KEGG: Kyoto Encyclopedia of Genes and Genomes
PPI: Protein–protein interaction
RNA-SEQ: RNA sequencing
ROS: Reactive oxygen species
SNpc: Substantia nigra pars compacta
TBST: Tween^®^20 detergent
TCM: Traditional Chinese Medicine
TEM: Transmission electron microscopy
TH: Tyrosine hydroxylase
