# Supplementary material for: Integrated network pharmacology and transcriptomics to explore the mechanism of compound Dihuang granule (CDG) protects dopaminergic neurons by regulating the Nrf2/HMOX1 pathway in the 6-OHDA/MPP+-induced model of Parkinson’s disease
Source: Chin Med. 2024 Dec 18;19:170. doi: 10.1186/s13020-024-01040-7 (PMC11654441; doi:10.1186/s13020-024-01040-7)
Supplement: Supplementary file 1 — Supplementary Material 1. [file 13020_2024_1040_MOESM1_ESM.docx]

| **Model** | **Name** | **Molecular Weight** | **RT [min]** | **Area (Max.)** |
| --- | --- | --- | --- | --- |
| NEG | (-)-Catechin gallate | 442.08928 | 5.995 | 9137.653512 |
| POS | (+)-Nootkatone | 218.16707 | 11.609 | 128525.5345 |
| POS | 2-Pyrrolidinecarboxylic acid | 115.06297 | 0.921 | 347020.9396 |
| NEG | 3,4-Dihydroxyphenylethanol | 154.06326 | 3.885 | 4877.249897 |
| NEG | 4',7-Di-O-methylnaringenin | 300.0995 | 8.561 | 10049.92214 |
| NEG | 4-Methoxyphenylacetic acid | 166.06318 | 5.697 | 19746.29001 |
| NEG | 4-Methoxysalicylic acid | 168.04251 | 3.255 | 13109.98536 |
| NEG | 4-Methylumbelliferone | 176.04756 | 6.335 | 15719.30619 |
| POS | 5-Hydroxymethylfurfural | 126.0314 | 2.934 | 428296.4199 |
| NEG | 6-Gingerol | 294.18287 | 10.134 | 3245.523378 |
| POS | 7-Hydroxycoumarin | 162.03153 | 4.876 | 408762.7324 |
| POS | 7-Methoxy-4-methylcoumarin | 190.0629 | 8.895 | 9260.173915 |
| POS | 7-Methoxycoumarin | 176.04741 | 6.367 | 59106.57104 |
| NEG | Abscisic acid | 264.13594 | 9.04 | 4684.722159 |
| POS | Acetophenone | 120.05716 | 5.525 | 39561.61453 |
| POS | Adenine | 135.05426 | 1.419 | 50126.24417 |
| POS | Adenosine | 267.09676 | 1.422 | 178938.6759 |
| POS | Agarotetrol | 318.11012 | 8.11 | 19796.24413 |
| POS | Albiflorin | 480.16204 | 6.708 | 398133.6512 |
| NEG | Androsin | 328.1154 | 5.696 | 24981.7913 |
| NEG | Apigenin-7-O-β-D-glucoside | 432.10518 | 7.638 | 2073.556459 |
| POS | Arglabin | 246.12551 | 8.051 | 19975.1925 |
| POS | Artemisinic acid | 234.16193 | 10.185 | 443568.6305 |
| POS | Artemisinin | 282.14653 | 6.312 | 41048.04962 |
| POS | Atractylenolide II | 232.14622 | 9.72 | 119257.4927 |
| NEG | Azelaic acid | 188.10504 | 6.833 | 42843.78677 |
| NEG | Bengenin | 328.07915 | 5.105 | 41916.20156 |
| NEG | Benzoylpaeoniflorin | 584.18817 | 8.002 | 500966.1123 |
| POS | Betaine | 117.0786 | 0.876 | 1012881.492 |
| NEG | Caffeic acid | 180.04248 | 5.268 | 128327.3812 |
| POS | Calycosin | 284.06832 | 6.377 | 20219.11502 |
| POS | Cantharidin | 196.07321 | 7.702 | 201292.1197 |
| NEG | Carnosol | 330.18263 | 10.011 | 10586.58393 |
| NEG | Catechin hydrate | 290.07905 | 4.438 | 1595.365062 |
| POS | Catechin hydrate | 290.07912 | 4.447 | 10949.18318 |
| NEG | Chlorogenic acid | 354.0948 | 4.897 | 625001.2866 |
| POS | Chlorogenic acid | 354.09472 | 4.869 | 144328.4072 |
| POS | Cinnamaldehyde | 132.05721 | 6.726 | 125070.5864 |
| NEG | Cinnamic acid | 148.05278 | 5.705 | 1216.151018 |
| NEG | Citric acid | 192.02719 | 0.942 | 278098.4224 |
| POS | Corynoxeine | 382.18813 | 6.681 | 7335383.677 |
| NEG | Crocetin | 328.16694 | 10.035 | 2828.8692 |
| POS | Cryptotanshinone | 296.14059 | 12.437 | 1002059.195 |
| POS | Curcumol | 236.17758 | 8.255 | 13533.20896 |
| NEG | Danshensu | 198.0529 | 3.352 | 317065.131 |
| POS | Demethoxyyangonin | 228.07868 | 9.412 | 26148.47368 |
| POS | Dihydrotanshinone I | 278.09409 | 11.357 | 350658.8621 |
| NEG | Ethyl ferulate | 222.08911 | 9.168 | 3121.778791 |
| NEG | Eurycomalactone | 348.15672 | 8.281 | 5649.580135 |
| NEG | Ferulic acid | 194.0578 | 4.987 | 51794.33645 |
| POS | Ferulic acid | 194.05789 | 7.511 | 92500.71455 |
| POS | Flavone | 222.06772 | 6.94 | 366992.1368 |
| NEG | Forsythoside E | 462.17296 | 4.271 | 38373.64602 |
| NEG | Fumaric acid | 116.01135 | 1.501 | 46450.11795 |
| NEG | Gallic acid | 170.02177 | 2.022 | 945856.0589 |
| NEG | Gardenoside | 404.13115 | 3.664 | 20237.58215 |
| NEG | Geniposidic acid | 374.12059 | 2.752 | 12020.83067 |
| POS | Glabrolide | 468.32359 | 7.19 | 15743.45597 |
| POS | Guanine | 151.04918 | 1.545 | 52329.11057 |
| POS | Guanosine | 283.09175 | 1.545 | 23298.45477 |
| POS | Hirsuteine | 366.19374 | 7.762 | 823496.049 |
| POS | Hirsutine | 368.17256 | 5.542 | 327709.6982 |
| NEG | Hyperoside | 464.09492 | 6.038 | 102781.3482 |
| POS | Hyperoside | 464.09511 | 6.041 | 41427.27217 |
| NEG | Iridin | 522.13673 | 6.102 | 33216.73351 |
| NEG | Isochlorogenic acid B | 516.12611 | 6.577 | 65400.73885 |
| NEG | Isoguanosine | 283.09144 | 1.53 | 6078.373012 |
| NEG | Isoimperatorin | 270.08903 | 8.39 | 4461.323122 |
| POS | Lithospermic acid | 538.10985 | 6.94 | 477527.3367 |
| NEG | Lithospermic acid | 538.11012 | 6.025 | 219034.3713 |
| POS | L-Leucine | 131.09462 | 1.558 | 59576.69191 |
| NEG | Loganic acid | 376.13637 | 4.467 | 214470.2992 |
| POS | L-Phenylalanine | 165.07889 | 2.587 | 62690.74187 |
| NEG | Manninotriose | 504.1682 | 2.593 | 5617.127572 |
| POS | Methyl 4-hydroxycinnamate | 178.06268 | 6.73 | 102959.6436 |
| NEG | Methyl gallate | 184.03726 | 4.351 | 7213.141068 |
| POS | Nardosinone | 250.15694 | 9.026 | 44386.27033 |
| NEG | Nardosinone | 250.15695 | 8.647 | 2828.223528 |
| POS | Naringenin | 272.06801 | 5.955 | 46539.85354 |
| POS | Nicotinamide | 122.04775 | 1.345 | 24256.25288 |
| POS | Norboldine | 313.13133 | 7.407 | 65309.05889 |
| NEG | Oxypaeoniflorin | 496.15739 | 4.788 | 114873.6861 |
| POS | Paeoniflorin | 480.162 | 5.534 | 1495540.501 |
| NEG | Paeoniflorin | 480.16112 | 5.535 | 1418809.961 |
| POS | Parthenolide | 248.14121 | 9.362 | 21421.68601 |
| NEG | p-Coumaric acid | 164.04768 | 6.021 | 6247.11302 |
| NEG | p-Hydroxybenzaldehyde | 122.03573 | 16.642 | 4932.338808 |
| POS | Pogostone | 224.10483 | 5.825 | 119744.8342 |
| POS | Protocatechualdehyde | 138.03159 | 4.736 | 64278.38121 |
| NEG | Protocatechuic acid | 154.02691 | 3.84 | 52166.3399 |
| NEG | Purpureaside C | 786.25624 | 5.238 | 12223.45447 |
| NEG | Pyrogallol | 126.03206 | 2.021 | 281739.495 |
| POS | Quercetin | 302.04231 | 6.043 | 168324.9107 |
| NEG | Quinic acid | 192.0635 | 0.883 | 431130.8857 |
| NEG | Rehmannioside A | 524.17309 | 2.545 | 20100.24255 |
| POS | Rhynchophylline | 384.20366 | 6.905 | 6070835.179 |
| NEG | Rosmarinic acid | 360.08397 | 6.739 | 339808.4657 |
| NEG | Rutin | 610.15211 | 5.883 | 76325.25259 |
| POS | Rutin | 610.15306 | 5.88 | 22653.17851 |
| NEG | Salvianolic acid A | 494.12041 | 6.736 | 742852.8484 |
| NEG | Salvianolic acid C | 492.10445 | 6.747 | 47562.72714 |
| NEG | Scopoletin | 192.04245 | 7.441 | 2552.604959 |
| POS | Scopoletin | 192.04218 | 4.952 | 16367.38717 |
| NEG | Shikimic acid | 174.05389 | 4.936 | 7016.784196 |
| NEG | Sibiricose A5 | 518.16273 | 5.293 | 1109.003243 |
| POS | Stachydrine | 143.09439 | 0.914 | 170653.8954 |
| NEG | Stachyose | 666.21962 | 0.824 | 181525.586 |
| NEG | Sucrose | 342.11576 | 0.856 | 122675.4812 |
| POS | Tanshinone IIA | 294.12528 | 13.467 | 235729.2971 |
| NEG | Vanillin | 152.04743 | 3.127 | 201419.778 |
| NEG | Verbascoside | 624.20393 | 6.009 | 61656.63422 |
| POS | Vincamine | 354.19383 | 7.425 | 105535.7214 |
| POS | α-Linolenic acid | 278.2245 | 12.233 | 48706.57972 |
| POS | β-Asarone | 208.11006 | 10.462 | 42619.04581 |
