# Supplementary material for: Integrated network pharmacology and transcriptomics to explore the mechanism of compound Dihuang granule (CDG) protects dopaminergic neurons by regulating the Nrf2/HMOX1 pathway in the 6-OHDA/MPP+-induced model of Parkinson’s disease
Source: Chin Med. 2024 Dec 18;19:170. doi: 10.1186/s13020-024-01040-7 (PMC11654441; doi:10.1186/s13020-024-01040-7)
Supplement: Supplementary file 8 — Supplementary Material 8. [file 13020_2024_1040_MOESM8_ESM.docx]

| **List of differentially expressed genes analysed by Sham VS Model RNA-seq** | | | | | |
| --- | --- | --- | --- | --- | --- |
| **GeneID** | **Gene_Name** | **Fold Change** | **log2(fc)** | **pval** | **qval** |
| ENSRNOG00000004692 | A1bg | 0.000779697 | 10.32479822 | 6.79222E-05 | 0.00660841 |
| ENSRNOG00000007247 | A2ml1 | 833.5866667 | -9.703188391 | 1.37779E-05 | 0.002349815 |
| ENSRNOG00000058890 | AABR07003274.1 | 0.101675464 | 3.297956522 | 0.000455665 | 0.022166694 |
| ENSRNOG00000054097 | AABR07004232.2 | 5.075756008 | -2.34362272 | 0.001573584 | 0.047543088 |
| ENSRNOG00000047124 | AABR07005775.1 | 0.151297177 | 2.724543023 | 0.00041704 | 0.020837311 |
| ENSRNOG00000027374 | AABR07005985.1 | 0.343258077 | 1.542634425 | 0.00158847 | 0.047746662 |
| ENSRNOG00000013993 | AABR07007000.1 | 4.12313211 | -2.043740688 | 1.63519E-06 | 0.000459157 |
| ENSRNOG00000061142 | AABR07007715.1 | 5.52727E-05 | 14.14307252 | 0.00111411 | 0.0395429 |
| ENSRNOG00000060406 | AABR07017159.1 | 30634.69 | -14.90287863 | 3.0079E-08 | 1.7559E-05 |
| ENSRNOG00000054194 | AABR07018116.1 | 0.13715879 | 2.866081008 | 0.000799203 | 0.031943622 |
| ENSRNOG00000024294 | AABR07019083.1 | 0.374324066 | 1.41764029 | 2.43515E-05 | 0.003440697 |
| ENSRNOG00000003952 | AABR07020879.1 | 0.180802211 | 2.467515777 | 0.000642798 | 0.027687746 |
| ENSRNOG00000054809 | AABR07026032.1 | 0.0799558 | 3.644653506 | 5.69656E-05 | 0.006048492 |
| ENSRNOG00000059081 | AABR07026032.3 | 0.078704385 | 3.667412168 | 1.09068E-05 | 0.001980789 |
| ENSRNOG00000014264 | AABR07027306.1 | 0.192846457 | 2.374475451 | 0.000177313 | 0.01225336 |
| ENSRNOG00000056802 | AABR07028749.2 | 2940.133333 | -11.52166587 | 0.001412907 | 0.04503234 |
| ENSRNOG00000053479 | AABR07029417.1 | 2.099275922 | -1.069891802 | 0.001470649 | 0.045957479 |
| ENSRNOG00000046001 | AABR07030823.1 | 0.449290508 | 1.154279511 | 0.000540245 | 0.024709794 |
| ENSRNOG00000055889 | AABR07030901.1 | 0.165270917 | 2.597095221 | 0.001553246 | 0.047206646 |
| ENSRNOG00000057826 | AABR07040412.1 | 0.379809212 | 1.396653198 | 0.001262959 | 0.042622239 |
| ENSRNOG00000051666 | AABR07042609.1 | 16710.20667 | -14.02844196 | 5.08397E-07 | 0.000184881 |
| ENSRNOG00000036484 | AABR07047044.1 | 0.011119461 | 6.490769317 | 0.000156886 | 0.011281749 |
| ENSRNOG00000030938 | AABR07049499.1 | 0.030123159 | 5.052983137 | 0.000754351 | 0.030943797 |
| ENSRNOG00000052899 | AABR07049886.2 | 0.203194925 | 2.299063722 | 0.000992919 | 0.0368828 |
| ENSRNOG00000052537 | AABR07050265.1 | 2.571950087 | -1.362862645 | 0.001497533 | 0.046396324 |
| ENSRNOG00000053592 | AABR07050646.1 | 0.082664771 | 3.59658355 | 6.70058E-05 | 0.00657695 |
| ENSRNOG00000042321 | AABR07052588.1 | 0.096231666 | 3.377344484 | 3.76266E-05 | 0.004662961 |
| ENSRNOG00000024494 | AABR07056633.1 | 0.202998096 | 2.300461901 | 0.00097235 | 0.036572038 |
| ENSRNOG00000051264 | AABR07059679.1 | 0.056928579 | 4.134703095 | 4.78697E-06 | 0.001083565 |
| ENSRNOG00000006857 | AABR07060833.1 | 3.273774267 | -1.710954848 | 0.00125935 | 0.042622239 |
| ENSRNOG00000036912 | AABR07062599.1 | 211.9529769 | -7.727600419 | 0.000365452 | 0.019210468 |
| ENSRNOG00000060463 | AABR07062799.2 | 0.293404185 | 1.769038644 | 0.00031868 | 0.017673211 |
| ENSRNOG00000058248 | AABR07064111.1 | 0.336630782 | 1.57076099 | 0.00044714 | 0.021799818 |
| ENSRNOG00000049123 | AABR07066510.1 | 0.306740132 | 1.704911161 | 0.000422323 | 0.020911593 |
| ENSRNOG00000053753 | AABR07070307.1 | 0.329247485 | 1.602755675 | 0.000980876 | 0.03681688 |
| ENSRNOG00000051221 | AABR07070310.1 | 0.042730509 | 4.548589704 | 4.6012E-05 | 0.005261263 |
| ENSRNOG00000062252 | AABR07072853.5 | 2.941076177 | -1.556344152 | 0.000561697 | 0.025377017 |
| ENSRNOG00000012892 | Abca4 | 0.092427641 | 3.435531834 | 0.00039481 | 0.020320336 |
| ENSRNOG00000052761 | AC095390.2 | 0.000306359 | 11.67248869 | 0.000407547 | 0.02059364 |
| ENSRNOG00000056454 | AC096600.1 | 0.144865011 | 2.787218909 | 0.000308715 | 0.017332371 |
| ENSRNOG00000062261 | AC111804.2 | 0.101462105 | 3.300987093 | 0.000206664 | 0.013362746 |
| ENSRNOG00000061650 | AC128059.5 | 0.144660934 | 2.789252728 | 0.001059506 | 0.038154249 |
| ENSRNOG00000042235 | AC130035.1 | 0.130957706 | 2.932827141 | 0.001584859 | 0.047746662 |
| ENSRNOG00000007816 | AC131483.1 | 0.079129919 | 3.659632911 | 0.001610778 | 0.048127044 |
| ENSRNOG00000046261 | Acp5 | 0.147519803 | 2.761019457 | 3.23363E-13 | 7.17317E-10 |
| ENSRNOG00000017786 | Acta1 | 0.095095475 | 3.394479495 | 2.07228E-41 | 4.59693E-37 |
| ENSRNOG00000056756 | Actn1 | 0.246000238 | 2.023268385 | 0.000267867 | 0.01597337 |
| ENSRNOG00000017833 | Actn2 | 0.086237526 | 3.535540393 | 1.0496E-05 | 0.001940273 |
| ENSRNOG00000019745 | Actn3 | 0.369542597 | 1.436187423 | 0.000998995 | 0.0368828 |
| ENSRNOG00000004828 | Acvr1c | 0.115500797 | 3.114025289 | 5.15504E-05 | 0.005605605 |
| ENSRNOG00000027463 | Adamts3 | 0.129542703 | 2.948500338 | 9.55353E-06 | 0.00187545 |
| ENSRNOG00000059479 | Adcy1 | 0.268951527 | 1.894581914 | 0.000948877 | 0.036042691 |
| ENSRNOG00000001821 | Adipoq | 0.000130012 | 12.90907239 | 4.92966E-09 | 3.64516E-06 |
| ENSRNOG00000060087 | Adra1b | 3.83065898 | -1.937592597 | 0.000926744 | 0.035567396 |
| ENSRNOG00000009299 | Adra2c | 0.122495777 | 3.02919608 | 9.7265E-05 | 0.008172839 |
| ENSRNOG00000002232 | Aff1 | 0.491163556 | 1.025724576 | 0.000271953 | 0.016087293 |
| ENSRNOG00000025584 | Agap2 | 0.173675855 | 2.525530896 | 0.000275005 | 0.016163377 |
| ENSRNOG00000008467 | Agbl2 | 0.123258705 | 3.020238553 | 0.000544171 | 0.024787179 |
| ENSRNOG00000012315 | Agmat | 0.172545109 | 2.534954512 | 0.000690143 | 0.029105421 |
| ENSRNOG00000017821 | Agxt2 | 1624.143333 | -10.66546324 | 1.93983E-05 | 0.00298828 |
| ENSRNOG00000037688 | Ak9 | 0.153816402 | 2.700718746 | 0.000668133 | 0.02855723 |
| ENSRNOG00000006410 | Akap5 | 0.064457311 | 3.955512175 | 1.92608E-06 | 0.000527485 |
| ENSRNOG00000002911 | Alb | 0.230961344 | 2.114276685 | 0.000344733 | 0.018651737 |
| ENSRNOG00000002331 | Aldh3a1 | 0.035913141 | 4.799344345 | 0.00032703 | 0.017924423 |
| ENSRNOG00000030869 | Aldoart2 | 0.155463406 | 2.685353068 | 6.51101E-07 | 0.000225678 |
| ENSRNOG00000022636 | Alpk1 | 0.460602875 | 1.118404678 | 0.000160326 | 0.011417647 |
| ENSRNOG00000015554 | Ankdd1a | 0.209476416 | 2.255140272 | 0.001494302 | 0.046360974 |
| ENSRNOG00000025037 | Ankk1 | 0.095543509 | 3.387698319 | 0.000124858 | 0.009650593 |
| ENSRNOG00000010888 | Ankrd33b | 0.084095758 | 3.571823153 | 0.000273943 | 0.016161906 |
| ENSRNOG00000042446 | Ankrd63 | 0.026193052 | 5.254672027 | 0.000276782 | 0.016200129 |
| ENSRNOG00000004731 | Ano3 | 0.071295135 | 3.810052553 | 0.000712085 | 0.029804096 |
| ENSRNOG00000011648 | Aqp1 | 0.012664187 | 6.303101776 | 0.000106318 | 0.00867078 |
| ENSRNOG00000043465 | Arc | 0.096130849 | 3.378856715 | 2.08321E-05 | 0.003165202 |
| ENSRNOG00000009347 | Arhgap25 | 0.465178249 | 1.104144455 | 5.71195E-09 | 4.08736E-06 |
| ENSRNOG00000024677 | Arhgap33 | 0.224654432 | 2.154220573 | 0.000645534 | 0.027751708 |
| ENSRNOG00000006946 | Arhgap9 | 0.368828925 | 1.438976291 | 4.17781E-07 | 0.00016259 |
| ENSRNOG00000011105 | Arl15 | 0.182794611 | 2.451704553 | 7.15831E-05 | 0.006815146 |
| ENSRNOG00000020770 | Arl4d | 0.169187385 | 2.563306094 | 5.22318E-05 | 0.005651987 |
| ENSRNOG00000036880 | Arl5c | 0.112234265 | 3.155414895 | 2.87286E-05 | 0.003816081 |
| ENSRNOG00000008919 | Arpp21 | 0.121884677 | 3.03641133 | 9.61344E-05 | 0.008170688 |
| ENSRNOG00000002256 | Art3 | 0.305258291 | 1.711897614 | 0.00072747 | 0.030069672 |
| ENSRNOG00000019557 | Asb18 | 33.67081297 | -5.07342665 | 0.000520698 | 0.024114094 |
| ENSRNOG00000051619 | Asb2 | 0.268385585 | 1.897620909 | 1.27775E-05 | 0.0022144 |
| ENSRNOG00000019985 | Asic4 | 0.306002579 | 1.708384283 | 0.000379067 | 0.019692859 |
| ENSRNOG00000002312 | Atp10d | 0.47311356 | 1.079741586 | 0.001131594 | 0.039781538 |
| ENSRNOG00000017912 | Atp2a3 | 5.456159056 | -2.447885702 | 1.50523E-05 | 0.00247718 |
| ENSRNOG00000004026 | Atp2b1 | 0.19976778 | 2.323604184 | 0.001006663 | 0.0368828 |
| ENSRNOG00000004049 | Baiap2 | 0.089418306 | 3.48328597 | 4.06119E-06 | 0.000958397 |
| ENSRNOG00000012215 | Baiap2l2 | 0.417801446 | 1.259110609 | 0.000726414 | 0.030069672 |
| ENSRNOG00000005776 | Bcl11b | 0.067727399 | 3.884116597 | 1.6193E-06 | 0.000459157 |
| ENSRNOG00000001304 | Bcr | 0.250263065 | 1.998482708 | 0.000320784 | 0.017745511 |
| ENSRNOG00000013717 | Bmp6 | 0.448777768 | 1.155926887 | 2.75716E-05 | 0.003688571 |
| ENSRNOG00000053384 | Bmp7 | 0.183305256 | 2.447679938 | 0.000555725 | 0.025209897 |
| ENSRNOG00000042163 | Btbd19 | 0.323137742 | 1.629778828 | 7.52927E-05 | 0.00701553 |
| ENSRNOG00000018570 | C1qtnf3 | 0.09232025 | 3.437209058 | 0.000572381 | 0.025758992 |
| ENSRNOG00000017766 | Ca12 | 0.108598526 | 3.202923573 | 3.00372E-05 | 0.003919499 |
| ENSRNOG00000060528 | Cacna1g | 3.049379733 | -1.608515817 | 4.10037E-05 | 0.004864094 |
| ENSRNOG00000003245 | Cacng1 | 0.000204948 | 12.2524565 | 3.74199E-05 | 0.004662961 |
| ENSRNOG00000011130 | Calca | 25.30310188 | -4.661242349 | 4.31707E-11 | 7.36658E-08 |
| ENSRNOG00000020325 | Calhm2 | 0.469359361 | 1.091235161 | 2.70033E-05 | 0.003652531 |
| ENSRNOG00000000886 | Caln1 | 0.261004611 | 1.937852802 | 0.001287748 | 0.042765694 |
| ENSRNOG00000017882 | Camk1d | 0.273962007 | 1.867952261 | 0.000444525 | 0.021768003 |
| ENSRNOG00000020478 | Camk4 | 0.192293959 | 2.378614657 | 0.000405414 | 0.020532626 |
| ENSRNOG00000001309 | Camkk2 | 0.280906741 | 1.831836849 | 0.001613208 | 0.048127044 |
| ENSRNOG00000047367 | Card14 | 0.102321522 | 3.288818471 | 4.11362E-15 | 1.30361E-11 |
| ENSRNOG00000017712 | Cartpt | 0.203696922 | 2.295503914 | 0.000351597 | 0.018807686 |
| ENSRNOG00000004372 | Cbln4 | 2.62205632 | -1.390698674 | 0.001443891 | 0.045822377 |
| ENSRNOG00000001701 | Cbr3 | 0.121262965 | 3.043789086 | 7.37009E-05 | 0.006939582 |
| ENSRNOG00000021109 | Ccdc114 | 0.292878968 | 1.771623497 | 0.001458324 | 0.045957479 |
| ENSRNOG00000010412 | Ccdc180 | 0.126658952 | 2.980979044 | 0.000310757 | 0.017364016 |
| ENSRNOG00000053015 | Ccdc189 | 0.449981935 | 1.15206101 | 0.000649936 | 0.027886926 |
| ENSRNOG00000004075 | Ccdc42 | 0.072386646 | 3.788132612 | 0.000601827 | 0.026510106 |
| ENSRNOG00000019321 | Cck | 5.073167107 | -2.342886683 | 0.000140221 | 0.010508553 |
| ENSRNOG00000015036 | Ccn2 | 0.353165883 | 1.501582116 | 3.30695E-15 | 1.22263E-11 |
| ENSRNOG00000057710 | Ccnd2 | 0.239188899 | 2.063777662 | 9.70467E-05 | 0.008172839 |
| ENSRNOG00000007483 | Ccnf | 0.257260941 | 1.958695663 | 0.000146762 | 0.010780203 |
| ENSRNOG00000024000 | Cd22 | 0.181534677 | 2.461682932 | 0.00165334 | 0.048706572 |
| ENSRNOG00000036674 | Cd7 | 5.068200754 | -2.341473672 | 6.7721E-05 | 0.00660841 |
| ENSRNOG00000001527 | Cd80 | 0.342431735 | 1.546111684 | 0.000808638 | 0.032262622 |
| ENSRNOG00000024799 | Cd93 | 0.441339877 | 1.180037989 | 1.13086E-06 | 0.000347324 |
| ENSRNOG00000032136 | Cdc42ep3 | 0.473220902 | 1.079414298 | 0.000317584 | 0.01765654 |
| ENSRNOG00000004148 | Cdk17 | 0.288801575 | 1.791849486 | 0.001360258 | 0.043932137 |
| ENSRNOG00000009258 | Cdk6 | 0.206456589 | 2.276089632 | 0.00032903 | 0.017977512 |
| ENSRNOG00000057347 | Cebpb | 0.372155134 | 1.426023957 | 0.001179669 | 0.040952433 |
| ENSRNOG00000017959 | Cfap100 | 0.259170075 | 1.948028945 | 0.000559122 | 0.025312255 |
| ENSRNOG00000056996 | Cfap65 | 0.107747914 | 3.214268155 | 0.000222077 | 0.013916207 |
| ENSRNOG00000058434 | Cfap99 | 0.223503523 | 2.161630525 | 0.000615069 | 0.026858417 |
| ENSRNOG00000009269 | Cga | 13194.54 | -13.68765343 | 0.000707762 | 0.029679185 |
| ENSRNOG00000025012 | Chat | 0.024443407 | 5.354410776 | 0.001210401 | 0.041822933 |
| ENSRNOG00000018385 | Chrm1 | 0.134713124 | 2.892037687 | 0.000956487 | 0.036269643 |
| ENSRNOG00000017556 | Chrm4 | 0.088482466 | 3.498464595 | 1.49003E-05 | 0.00247718 |
| ENSRNOG00000016267 | Chst15 | 0.132576252 | 2.915105725 | 4.20481E-05 | 0.004935204 |
| ENSRNOG00000020377 | Cideb | 0.000569149 | 10.77890669 | 0.001009853 | 0.0368828 |
| ENSRNOG00000016837 | Ckm | 0.020151397 | 5.632976332 | 3.61337E-17 | 2.00388E-13 |
| ENSRNOG00000018752 | Clcf1 | 0.054817227 | 4.189226839 | 1.13275E-05 | 0.001994262 |
| ENSRNOG00000003654 | Cldn9 | 0.341397699 | 1.55047476 | 0.000290358 | 0.016817245 |
| ENSRNOG00000026870 | Clic6 | 0.020713986 | 5.593251004 | 0.000437355 | 0.021464252 |
| ENSRNOG00000011332 | Clspn | 0.05764933 | 4.116552337 | 1.59716E-06 | 0.000459157 |
| ENSRNOG00000023803 | Cmya5 | 0.141603292 | 2.820073289 | 2.37306E-05 | 0.003418281 |
| ENSRNOG00000004778 | Cnga1 | 30.74480458 | -4.942270731 | 0.001442416 | 0.045822377 |
| ENSRNOG00000007014 | Cnksr2 | 0.175276418 | 2.512296186 | 0.000841815 | 0.033051307 |
| ENSRNOG00000012821 | Cnmd | 0.000146447 | 12.73733397 | 0.000603507 | 0.026510106 |
| ENSRNOG00000008223 | Cnr1 | 0.233238904 | 2.100119646 | 0.000961647 | 0.036403091 |
| ENSRNOG00000032517 | Cntn6 | 2.203961596 | -1.140099085 | 0.000689153 | 0.029105421 |
| ENSRNOG00000005286 | Coch | 0.179111905 | 2.481066863 | 0.000836141 | 0.033003766 |
| ENSRNOG00000001229 | Col18a1 | 0.46204926 | 1.113881427 | 0.000529997 | 0.024341449 |
| ENSRNOG00000007657 | Col27a1 | 0.493075162 | 1.020120515 | 0.000790768 | 0.031816417 |
| ENSRNOG00000058560 | Col2a1 | 3.412170999 | -1.770689948 | 0.001116426 | 0.039561793 |
| ENSRNOG00000010841 | Col8a2 | 0.082613317 | 3.597481834 | 0.000241699 | 0.01471685 |
| ENSRNOG00000004146 | Coro7 | 0.403718561 | 1.308578178 | 0.001449772 | 0.045943289 |
| ENSRNOG00000011913 | Cp | 0.499593634 | 1.001173001 | 0.000417066 | 0.020837311 |
| ENSRNOG00000000522 | Cpne5 | 0.221747568 | 2.173009811 | 0.00146898 | 0.045957479 |
| ENSRNOG00000015397 | Cpne7 | 3.876234622 | -1.954655897 | 6.74588E-11 | 1.06888E-07 |
| ENSRNOG00000023077 | Cpne9 | 3.32376677 | -1.732819151 | 0.001131346 | 0.039781538 |
| ENSRNOG00000013704 | Cps1 | 2.897034503 | -1.534576867 | 9.72261E-06 | 0.001890857 |
| ENSRNOG00000023633 | Crabp1 | 0.171127219 | 2.546858842 | 4.97091E-06 | 0.001113835 |
| ENSRNOG00000025498 | Crb2 | 0.08485696 | 3.558823194 | 0.001264158 | 0.042622239 |
| ENSRNOG00000012703 | Crh | 12.24436468 | -3.614046014 | 9.82376E-06 | 0.001890857 |
| ENSRNOG00000022421 | Crtc1 | 0.370868167 | 1.431021654 | 0.00127004 | 0.042622239 |
| ENSRNOG00000010524 | Cryab | 2.023266312 | -1.016686227 | 0.000918878 | 0.035348059 |
| ENSRNOG00000061215 | Crym | 0.048788793 | 4.357306412 | 5.0252E-08 | 2.85831E-05 |
| ENSRNOG00000049782 | Csf2ra | 0.462497281 | 1.11248321 | 5.95537E-05 | 0.006207946 |
| ENSRNOG00000022957 | Ctxn3 | 2.666111067 | -1.414736883 | 6.63839E-05 | 0.006574082 |
| ENSRNOG00000026647 | Cxcl16 | 0.274659993 | 1.86428131 | 0.001308986 | 0.042954489 |
| ENSRNOG00000003622 | Cybb | 2.160788999 | -1.1115582 | 7.94183E-05 | 0.007279906 |
| ENSRNOG00000009620 | Cybrd1 | 0.444560081 | 1.169549685 | 0.000727918 | 0.030069672 |
| ENSRNOG00000012458 | Cyp2e1 | 2.183628642 | -1.126727525 | 7.55854E-05 | 0.00701553 |
| ENSRNOG00000004772 | Cytip | 0.054905274 | 4.186911453 | 1.47667E-10 | 1.94399E-07 |
| ENSRNOG00000008834 | Dach1 | 0.132664932 | 2.914141027 | 0.000215718 | 0.013594499 |
| ENSRNOG00000022921 | Dact2 | 0.255673396 | 1.967626043 | 8.21305E-05 | 0.007466809 |
| ENSRNOG00000033026 | Dclk3 | 0.210602069 | 2.247408485 | 0.000768596 | 0.031317889 |
| ENSRNOG00000059605 | Ddn | 0.056574876 | 4.143694685 | 0.000303471 | 0.017217146 |
| ENSRNOG00000018716 | Dennd2c | 0.423986485 | 1.237909817 | 5.68521E-06 | 0.001229002 |
| ENSRNOG00000023465 | Depp1 | 0.185729292 | 2.428726727 | 2.649E-12 | 5.34207E-09 |
| ENSRNOG00000030771 | Dgkb | 0.117547266 | 3.088687107 | 2.20246E-05 | 0.003295346 |
| ENSRNOG00000010065 | Dgkh | 0.1421468 | 2.814546476 | 0.001015717 | 0.0368828 |
| ENSRNOG00000012573 | Dlgap2 | 0.11963213 | 3.063323184 | 0.000126711 | 0.009759815 |
| ENSRNOG00000004278 | Dlx3 | 1174.446667 | -10.19776548 | 0.000118069 | 0.009349802 |
| ENSRNOG00000010905 | Dlx5 | 0.056653703 | 4.141685945 | 1.49368E-05 | 0.00247718 |
| ENSRNOG00000010822 | Dlx6 | 0.067202514 | 3.895340987 | 0.000185592 | 0.01252471 |
| ENSRNOG00000055934 | Dmkn | 0.081396885 | 3.618882614 | 0.000119035 | 0.009349802 |
| ENSRNOG00000016284 | Dmrt3 | 0.000334755 | 11.54460548 | 2.05774E-10 | 2.40247E-07 |
| ENSRNOG00000026914 | Dnah1 | 0.18904904 | 2.403167569 | 0.000493193 | 0.023424989 |
| ENSRNOG00000005451 | Dnah11 | 0.244698683 | 2.030921758 | 2.51595E-06 | 0.000664422 |
| ENSRNOG00000052688 | Dnah2 | 0.148716388 | 2.749364463 | 6.55631E-05 | 0.006551291 |
| ENSRNOG00000015581 | Dnah6 | 0.123570798 | 3.016590241 | 0.001352916 | 0.043829253 |
| ENSRNOG00000021573 | Dpy19l3 | 0.352727596 | 1.503373644 | 0.001267924 | 0.042622239 |
| ENSRNOG00000025860 | Drc7 | 0.110755864 | 3.174545007 | 0.00050307 | 0.023743849 |
| ENSRNOG00000006931 | Eepd1 | 0.495288773 | 1.013658175 | 0.000528047 | 0.024341449 |
| ENSRNOG00000012618 | Efhb | 0.099060999 | 3.335539013 | 0.000519463 | 0.024107215 |
| ENSRNOG00000042729 | Efhc1 | 0.307231739 | 1.702600834 | 7.61745E-05 | 0.007040742 |
| ENSRNOG00000014648 | Efnb2 | 0.199989003 | 2.322007426 | 0.00010332 | 0.008520241 |
| ENSRNOG00000000640 | Egr2 | 0.048572841 | 4.363706316 | 5.6771E-10 | 5.72432E-07 |
| ENSRNOG00000017828 | Egr3 | 0.097024028 | 3.365514111 | 4.3345E-05 | 0.005015684 |
| ENSRNOG00000015719 | Egr4 | 0.028159721 | 5.150223174 | 2.50487E-08 | 1.50177E-05 |
| ENSRNOG00000019124 | Elovl3 | 1455.223333 | -10.50702487 | 0.000356373 | 0.018992095 |
| ENSRNOG00000033575 | Emid1 | 0.341544502 | 1.549854525 | 9.60034E-05 | 0.008170688 |
| ENSRNOG00000018631 | Enkur | 0.260999998 | 1.9378783 | 0.000854629 | 0.033377186 |
| ENSRNOG00000030622 | Enox2 | 0.415721263 | 1.266311555 | 0.001100579 | 0.039188041 |
| ENSRNOG00000023389 | Ephx4 | 0.230252461 | 2.118711519 | 0.001278132 | 0.042699999 |
| ENSRNOG00000012619 | Epor | 0.170616169 | 2.551173718 | 0.001263497 | 0.042622239 |
| ENSRNOG00000020426 | Erf | 0.48918771 | 1.031539934 | 0.000715207 | 0.029822265 |
| ENSRNOG00000036918 | Etfbkmt | 0.450784715 | 1.149489497 | 3.57629E-05 | 0.00450755 |
| ENSRNOG00000045743 | Etnppl | 2.512437739 | -1.329087845 | 2.79767E-06 | 0.000713342 |
| ENSRNOG00000046734 | Evx1 | 0.000363333 | 11.42642027 | 8.20811E-10 | 7.91655E-07 |
| ENSRNOG00000018524 | Ezr | 0.290032068 | 1.785715669 | 0.000234996 | 0.014480294 |
| ENSRNOG00000015139 | F12 | 0.147410631 | 2.762087523 | 4.71092E-05 | 0.005322764 |
| ENSRNOG00000016325 | F2 | 3.453415217 | -1.788023805 | 2.36769E-11 | 4.37688E-08 |
| ENSRNOG00000057855 | F5 | 0.00324388 | 8.268063978 | 0.000624078 | 0.027038922 |
| ENSRNOG00000033261 | Fam107a | 0.167559622 | 2.577253561 | 0.000104611 | 0.008594774 |
| ENSRNOG00000052758 | Fam49a | 0.25847522 | 1.951902116 | 0.000110332 | 0.008924074 |
| ENSRNOG00000051941 | Fam78b | 0.440978623 | 1.181219375 | 0.00029374 | 0.016911841 |
| ENSRNOG00000011774 | Fblim1 | 0.400155452 | 1.321367528 | 0.001085731 | 0.038721483 |
| ENSRNOG00000043035 | Fbxl13 | 0.276652833 | 1.853851396 | 0.000371095 | 0.019452021 |
| ENSRNOG00000022248 | Fbxl16 | 0.148160456 | 2.754767654 | 0.000150451 | 0.011014726 |
| ENSRNOG00000009206 | Fezf2 | 0.079937548 | 3.644982873 | 0.001004159 | 0.0368828 |
| ENSRNOG00000028064 | Fhad1 | 0.41129628 | 1.281750071 | 0.001375855 | 0.044232744 |
| ENSRNOG00000011521 | Filip1 | 0.31238169 | 1.678618201 | 0.000674026 | 0.028701679 |
| ENSRNOG00000024089 | Fndc3b | 0.388717526 | 1.363205941 | 0.00152871 | 0.046838922 |
| ENSRNOG00000019902 | Folr1 | 0.027096129 | 5.205769424 | 0.000187007 | 0.012570838 |
| ENSRNOG00000046667 | Fosb | 0.184620168 | 2.43736793 | 5.55342E-10 | 5.72432E-07 |
| ENSRNOG00000047891 | Foxg1 | 0.028489026 | 5.133449872 | 2.21344E-05 | 0.003295346 |
| ENSRNOG00000013397 | Foxo1 | 0.300245614 | 1.735784925 | 0.000688937 | 0.029105421 |
| ENSRNOG00000009184 | Foxp1 | 0.162892291 | 2.618009763 | 7.38287E-05 | 0.006939582 |
| ENSRNOG00000022309 | Frem1 | 0.113927545 | 3.133811498 | 0.000140933 | 0.010526311 |
| ENSRNOG00000021670 | Frem2 | 0.041617008 | 4.586682928 | 1.96749E-08 | 1.21235E-05 |
| ENSRNOG00000007329 | Frmd6 | 0.327065737 | 1.612347463 | 6.38212E-05 | 0.00640609 |
| ENSRNOG00000004898 | Fshb | 2040.533333 | -10.99473056 | 0.000176131 | 0.012248041 |
| ENSRNOG00000022619 | Fth1 | 0.451843303 | 1.146105555 | 9.38705E-05 | 0.008121496 |
| ENSRNOG00000008475 | Fut9 | 0.443007604 | 1.174596633 | 0.001382033 | 0.044367049 |
| ENSRNOG00000061031 | Fzd8 | 0.384323994 | 1.379605047 | 0.00161174 | 0.048127044 |
| ENSRNOG00000008431 | Gabbr2 | 2.576324319 | -1.365314218 | 0.000136665 | 0.010346879 |
| ENSRNOG00000002349 | Gabra2 | 0.303948327 | 1.718102018 | 0.001268698 | 0.042622239 |
| ENSRNOG00000061182 | Gabre | 3.437427619 | -1.781329336 | 0.001512703 | 0.046502455 |
| ENSRNOG00000040301 | Galntl5 | 0.000371475 | 11.39444662 | 9.44686E-05 | 0.008121496 |
| ENSRNOG00000049361 | Gas7 | 0.242310285 | 2.045072455 | 0.001073094 | 0.038518504 |
| ENSRNOG00000013369 | Gbx1 | 0.000370476 | 11.39833042 | 0.000337512 | 0.018305682 |
| ENSRNOG00000019495 | Gbx2 | 17.18380883 | -4.102977944 | 1.90463E-08 | 1.21235E-05 |
| ENSRNOG00000018282 | Gda | 0.087781339 | 3.509941918 | 2.2451E-05 | 0.003310472 |
| ENSRNOG00000051993 | Gdf10 | 0.211509355 | 2.241206621 | 0.000285337 | 0.016569687 |
| ENSRNOG00000011207 | Gh1 | 98.90781043 | -6.628012545 | 0.001685814 | 0.049531685 |
| ENSRNOG00000011599 | Gldc | 0.177780701 | 2.491829377 | 6.84264E-05 | 0.006628398 |
| ENSRNOG00000025120 | Gli1 | 2.945568344 | -1.558546027 | 1.49593E-06 | 0.000440581 |
| ENSRNOG00000036826 | Glycam1 | 9.25637E-05 | 13.39919424 | 4.33797E-10 | 4.81146E-07 |
| ENSRNOG00000019857 | Gng7 | 0.068174794 | 3.874617762 | 0.000508374 | 0.023841984 |
| ENSRNOG00000027658 | Gpr101 | 0.292456087 | 1.773708078 | 0.0002444 | 0.014772565 |
| ENSRNOG00000055673 | Gpr52 | 0.091313124 | 3.453033965 | 1.24641E-05 | 0.002177092 |
| ENSRNOG00000049580 | Gpr6 | 0.037328116 | 4.743593494 | 0.000511451 | 0.023885298 |
| ENSRNOG00000026953 | Gpr88 | 0.039778774 | 4.651857363 | 0.00020421 | 0.013245613 |
| ENSRNOG00000023657 | Gprin3 | 0.195226547 | 2.356778848 | 0.000371801 | 0.019452021 |
| ENSRNOG00000013604 | Gpx4 | 0.407823108 | 1.293984571 | 0.001453051 | 0.045957479 |
| ENSRNOG00000007346 | Grasp | 0.248169979 | 2.010599493 | 0.000119264 | 0.009349802 |
| ENSRNOG00000001575 | Grik1 | 2.806419955 | -1.488730911 | 3.44959E-06 | 0.000850247 |
| ENSRNOG00000009726 | Grip2 | 2.950916164 | -1.561162934 | 0.000376938 | 0.019628224 |
| ENSRNOG00000013171 | Grm2 | 3.85245076 | -1.945776517 | 7.37935E-09 | 5.1155E-06 |
| ENSRNOG00000016429 | Grm5 | 0.189539342 | 2.399430761 | 0.000402312 | 0.020469014 |
| ENSRNOG00000000233 | Grm6 | 7.585024423 | -2.923153826 | 9.18112E-05 | 0.008018297 |
| ENSRNOG00000016999 | Grp | 9.090332129 | -3.184333006 | 1.50644E-16 | 6.68349E-13 |
| ENSRNOG00000012302 | Gucy1a1 | 0.19351722 | 2.369466146 | 0.000185756 | 0.01252471 |
| ENSRNOG00000004601 | Hao1 | 51.69968491 | -5.692083583 | 6.44157E-08 | 3.40222E-05 |
| ENSRNOG00000014117 | Hmox1 | 0.17187563 | 2.540563096 | 0.000255951 | 0.015386872 |
| ENSRNOG00000006979 | Hpca | 0.121141857 | 3.045230665 | 0.000118651 | 0.009349802 |
| ENSRNOG00000001338 | Hpd | 3.277484116 | -1.712588788 | 3.57015E-07 | 0.000143994 |
| ENSRNOG00000017625 | Htr2b | 0.000638185 | 10.61373729 | 0.000142532 | 0.010574549 |
| ENSRNOG00000049761 | Htr6 | 0.108233239 | 3.207784465 | 3.44381E-05 | 0.004365375 |
| ENSRNOG00000017178 | Hydin | 0.239030469 | 2.064733563 | 0.000689834 | 0.029105421 |
| ENSRNOG00000020694 | Icam5 | 0.083085635 | 3.589257128 | 3.69877E-06 | 0.000882256 |
| ENSRNOG00000016690 | Idi1 | 2.19770934 | -1.136000594 | 0.000838814 | 0.033050454 |
| ENSRNOG00000004273 | Ifitm1 | 0.061394204 | 4.025753721 | 0.001587199 | 0.047746662 |
| ENSRNOG00000016308 | Il10ra | 0.371117288 | 1.430052887 | 3.77446E-09 | 2.8872E-06 |
| ENSRNOG00000028650 | Inf2 | 0.2685559 | 1.896705677 | 0.000142011 | 0.010571223 |
| ENSRNOG00000014320 | Inhba | 0.098564646 | 3.342785926 | 1.80839E-05 | 0.002837028 |
| ENSRNOG00000025406 | Iqgap2 | 0.205067694 | 2.285827864 | 0.001231493 | 0.0423538 |
| ENSRNOG00000027894 | Iqgap3 | 0.131996833 | 2.921424778 | 2.58937E-05 | 0.003557846 |
| ENSRNOG00000061070 | Irf4 | 0.485512549 | 1.042419509 | 0.000579704 | 0.025978946 |
| ENSRNOG00000012556 | Isl1 | 0.106329882 | 3.233380994 | 0.000527398 | 0.024341449 |
| ENSRNOG00000006723 | Itga11 | 0.269026807 | 1.89417816 | 0.000213853 | 0.013515406 |
| ENSRNOG00000022071 | Itga2b | 0.159913397 | 2.644637284 | 0.000128773 | 0.009850253 |
| ENSRNOG00000008346 | Itgb6 | 0.056916687 | 4.135004509 | 3.85172E-05 | 0.004720589 |
| ENSRNOG00000006860 | Itk | 0.074292725 | 3.750635236 | 4.42659E-05 | 0.005087823 |
| ENSRNOG00000005284 | Itpka | 0.054333857 | 4.20200473 | 0.000461068 | 0.022331619 |
| ENSRNOG00000019719 | Kcna5 | 0.121887695 | 3.036375604 | 1.7425E-05 | 0.002780851 |
| ENSRNOG00000056697 | Kcnab1 | 0.176232972 | 2.504444225 | 0.00023496 | 0.014480294 |
| ENSRNOG00000014686 | Kcnd3 | 2.400986945 | -1.263627559 | 0.001012218 | 0.0368828 |
| ENSRNOG00000024310 | Kcnf1 | 0.125142138 | 2.998360437 | 0.000108649 | 0.008828422 |
| ENSRNOG00000054314 | Kcng1 | 0.168834856 | 2.566315319 | 0.000616873 | 0.026881777 |
| ENSRNOG00000003841 | Kcnh1 | 0.206919093 | 2.272861322 | 0.00011928 | 0.009349802 |
| ENSRNOG00000057315 | Kcnh3 | 0.045626341 | 4.453989212 | 0.000160587 | 0.011417647 |
| ENSRNOG00000018790 | Kcnh4 | 0.116943338 | 3.096118415 | 0.000242815 | 0.01471685 |
| ENSRNOG00000018018 | Kcnip2 | 0.118471683 | 3.077385824 | 7.99312E-05 | 0.007296763 |
| ENSRNOG00000016057 | Kcnj13 | 0.013776149 | 6.181683507 | 0.000210962 | 0.013447616 |
| ENSRNOG00000013869 | Kcnj4 | 0.029134641 | 5.101120639 | 1.8184E-05 | 0.002837028 |
| ENSRNOG00000002653 | Kcnk2 | 0.206134404 | 2.278342784 | 0.000200219 | 0.013063102 |
| ENSRNOG00000019440 | Kcnn4 | 0.208670653 | 2.26070038 | 0.00097088 | 0.036572038 |
| ENSRNOG00000013781 | Kcnq5 | 0.141507916 | 2.821045335 | 6.05519E-05 | 0.006218622 |
| ENSRNOG00000011369 | Kcns2 | 0.085756493 | 3.543610285 | 2.76023E-05 | 0.003688571 |
| ENSRNOG00000004117 | Kcnv1 | 0.116364776 | 3.103273675 | 4.05222E-05 | 0.004848225 |
| ENSRNOG00000016467 | Kctd1 | 0.318378578 | 1.651184825 | 0.001462494 | 0.045957479 |
| ENSRNOG00000001092 | Kl | 0.019833047 | 5.655949828 | 0.001272335 | 0.042634757 |
| ENSRNOG00000033694 | Klf16 | 0.246211715 | 2.022028688 | 0.000279133 | 0.016251984 |
| ENSRNOG00000008785 | Klf5 | 0.098977451 | 3.336756306 | 6.23301E-07 | 0.000219471 |
| ENSRNOG00000014215 | Klf9 | 0.394815323 | 1.340750112 | 0.00128781 | 0.042765694 |
| ENSRNOG00000029441 | Klhl2 | 0.299644004 | 1.73867859 | 0.001320586 | 0.043271138 |
| ENSRNOG00000024479 | Klhl34 | 0.170847897 | 2.549215607 | 0.000218605 | 0.013737435 |
| ENSRNOG00000051487 | Kremen1 | 0.133417311 | 2.905982225 | 1.09831E-05 | 0.001980789 |
| ENSRNOG00000049495 | Krt71 | 0.146664606 | 2.769407341 | 0.000299352 | 0.01711477 |
| ENSRNOG00000005457 | Lamp5 | 0.147381223 | 2.762375368 | 5.94262E-05 | 0.006207946 |
| ENSRNOG00000009946 | Ldlr | 2.51648971 | -1.331412699 | 0.000411301 | 0.020689117 |
| ENSRNOG00000016879 | Ldlrad4 | 0.473402615 | 1.07886042 | 5.4398E-05 | 0.005857817 |
| ENSRNOG00000032569 | Lingo3 | 0.111486576 | 3.165058093 | 9.37062E-06 | 0.001855969 |
| ENSRNOG00000060775 | Lmo7 | 0.120625718 | 3.051390568 | 5.13081E-05 | 0.005605605 |
| ENSRNOG00000045785 | LOC100359687 | 2.18151068 | -1.125327536 | 1.99679E-05 | 0.003054808 |
| ENSRNOG00000048623 | LOC100911564 | 2.147232465 | -1.102478389 | 9.01289E-05 | 0.007933846 |
| ENSRNOG00000050450 | LOC100911951 | 0.144246644 | 2.79339034 | 0.000325908 | 0.017924423 |
| ENSRNOG00000045683 | LOC102553715 | 0.299888929 | 1.737499833 | 0.001297881 | 0.042843587 |
| ENSRNOG00000047471 | LOC103689994 | 0.086946385 | 3.523730143 | 0.000588705 | 0.02611849 |
| ENSRNOG00000020133 | LOC108348044 | 0.032909824 | 4.925337874 | 1.3714E-07 | 6.47271E-05 |
| ENSRNOG00000046751 | LOC688649 | 8050.926667 | -12.97493913 | 1.37398E-05 | 0.002349815 |
| ENSRNOG00000008680 | Loxl1 | 0.276036921 | 1.857066849 | 5.97728E-05 | 0.006207946 |
| ENSRNOG00000012181 | Lpl | 0.213187068 | 2.229808168 | 0.000584331 | 0.026008799 |
| ENSRNOG00000029308 | Lrmp | 0.471680727 | 1.08411744 | 1.10146E-06 | 0.000347324 |
| ENSRNOG00000030180 | Lrrc10b | 0.047359614 | 4.400198879 | 0.000401592 | 0.020469014 |
| ENSRNOG00000047890 | Lrrc23 | 0.279416163 | 1.839512618 | 0.000733873 | 0.030203173 |
| ENSRNOG00000014937 | Lrrc71 | 0.266531474 | 1.90762219 | 0.001026644 | 0.037151801 |
| ENSRNOG00000011826 | Lzts1 | 0.180257628 | 2.471867783 | 0.000258521 | 0.015499394 |
| ENSRNOG00000021231 | Lzts3 | 0.221833975 | 2.172447755 | 0.000823747 | 0.032689038 |
| ENSRNOG00000016037 | Mafb | 0.413619594 | 1.273623563 | 5.14495E-05 | 0.005605605 |
| ENSRNOG00000000800 | Man1a1 | 0.296569405 | 1.753558322 | 4.72697E-05 | 0.005322764 |
| ENSRNOG00000015439 | Man2a1 | 0.486314127 | 1.040039594 | 0.001224218 | 0.042223102 |
| ENSRNOG00000017905 | Map1lc3b | 0.394961889 | 1.340214646 | 0.000316625 | 0.017647488 |
| ENSRNOG00000015401 | Mapk4 | 0.21026334 | 2.249730758 | 5.84349E-05 | 0.006172672 |
| ENSRNOG00000003703 | Mcm6 | 0.404301587 | 1.30649623 | 0.000198002 | 0.01299488 |
| ENSRNOG00000013282 | Mctp1 | 0.088018647 | 3.506046992 | 1.72901E-06 | 0.000479434 |
| ENSRNOG00000053787 | Mdfic | 0.264502172 | 1.918648528 | 8.22233E-07 | 0.000276357 |
| ENSRNOG00000004730 | Meis2 | 0.0934983 | 3.41891605 | 5.7065E-06 | 0.001229002 |
| ENSRNOG00000013598 | Melk | 0.168519779 | 2.569010166 | 0.000346835 | 0.01871982 |
| ENSRNOG00000006588 | Meox2 | 0.080520613 | 3.634498032 | 0.001120634 | 0.039594765 |
| ENSRNOG00000025415 | Mettl11b | 0.020542832 | 5.605221093 | 1.14298E-06 | 0.000347324 |
| ENSRNOG00000040350 | Mir675 | 4.28174E-05 | 14.51144465 | 0.001709873 | 0.049879084 |
| ENSRNOG00000005934 | Mlip | 0.216401491 | 2.208217655 | 0.000622258 | 0.027012806 |
| ENSRNOG00000009514 | Mme | 0.242681509 | 2.042863908 | 0.000269927 | 0.016053073 |
| ENSRNOG00000010947 | Mmp14 | 0.245388038 | 2.026863174 | 0.000151318 | 0.011041743 |
| ENSRNOG00000057867 | Mns1 | 0.221700787 | 2.1733142 | 0.000241304 | 0.01471685 |
| ENSRNOG00000029211 | Mogat3 | 53.56258052 | -5.743153563 | 0.001178764 | 0.040952433 |
| ENSRNOG00000003171 | Mpz | 0.023683584 | 5.399968745 | 0.000333514 | 0.018177767 |
| ENSRNOG00000001548 | Nrf2 | 0.076745837 | 3.703767696 | 7.89691E-05 | 0.00726876 |
| ENSRNOG00000043098 | Mt2A | 0.38480171 | 1.377812885 | 6.35529E-06 | 0.00134266 |
| ENSRNOG00000056817 | Muc6 | 0.13095409 | 2.932866975 | 0.000296363 | 0.016987658 |
| ENSRNOG00000056493 | Mybpc1 | 3.775475752 | -1.916658452 | 4.17106E-05 | 0.004921632 |
| ENSRNOG00000019627 | Mybpc2 | 0.129264984 | 2.95159657 | 2.31466E-09 | 1.97485E-06 |
| ENSRNOG00000049695 | Myh4 | 0.0653177 | 3.936382203 | 9.73262E-38 | 1.07949E-33 |
| ENSRNOG00000016983 | Myh7 | 0.292409838 | 1.773936243 | 0.000583067 | 0.026008799 |
| ENSRNOG00000013262 | Myl1 | 0.003859909 | 8.017217496 | 0.001166876 | 0.040699399 |
| ENSRNOG00000008356 | Myo5c | 0.043786188 | 4.513380347 | 0.000185703 | 0.01252471 |
| ENSRNOG00000013641 | Myo7a | 0.271139961 | 1.882890338 | 1.33596E-09 | 1.23481E-06 |
| ENSRNOG00000008415 | Nab2 | 0.283611869 | 1.818010186 | 0.000466951 | 0.022518205 |
| ENSRNOG00000015768 | Nat8f5 | 3.422470016 | -1.775037902 | 0.001693906 | 0.049637939 |
| ENSRNOG00000004968 | Ncapg2 | 0.422565097 | 1.242754486 | 0.000197875 | 0.01299488 |
| ENSRNOG00000021553 | Nckap5 | 0.431566403 | 1.21234554 | 0.001374295 | 0.044232744 |
| ENSRNOG00000019768 | Ncoa4 | 0.343844197 | 1.540173096 | 1.92932E-08 | 1.21235E-05 |
| ENSRNOG00000015675 | Nell1 | 2.610210362 | -1.384166081 | 0.001703016 | 0.049839063 |
| ENSRNOG00000014006 | Neto1 | 0.127179114 | 2.975066327 | 0.000101117 | 0.008401018 |
| ENSRNOG00000027606 | Neurl1b | 0.119353037 | 3.066692821 | 1.01005E-05 | 0.001915036 |
| ENSRNOG00000003872 | NEWGENE_620180 | 0.332442428 | 1.588823575 | 1.88065E-07 | 8.34368E-05 |
| ENSRNOG00000012512 | Nexn | 0.196360463 | 2.34842362 | 0.00159682 | 0.047932681 |
| ENSRNOG00000016653 | Ngef | 0.086701653 | 3.527796687 | 0.001002764 | 0.0368828 |
| ENSRNOG00000005540 | Nin | 0.382566444 | 1.386217758 | 0.00094662 | 0.036018651 |
| ENSRNOG00000008644 | Nkx2-1 | 0.115643624 | 3.112242372 | 4.15505E-06 | 0.000970227 |
| ENSRNOG00000012728 | Nkx2-2 | 2.544168592 | -1.347194275 | 4.34121E-05 | 0.005015684 |
| ENSRNOG00000002164 | Nmu | 0.228523855 | 2.129583323 | 0.001306257 | 0.042954489 |
| ENSRNOG00000015863 | Npsr1 | 4.538883331 | -2.182337405 | 0.000208548 | 0.013370561 |
| ENSRNOG00000005964 | Nr4a3 | 0.22439448 | 2.155890908 | 4.26499E-07 | 0.000163121 |
| ENSRNOG00000012682 | Nr5a1 | 0.000489453 | 10.99654175 | 8.99544E-06 | 0.001797709 |
| ENSRNOG00000013290 | Nrip3 | 2.486692765 | -1.314228271 | 0.001646685 | 0.048639687 |
| ENSRNOG00000004179 | Nts | 0.402186412 | 1.314063753 | 0.000830578 | 0.032842636 |
| ENSRNOG00000009243 | Oaf | 0.396496594 | 1.334619621 | 1.42948E-13 | 3.52334E-10 |
| ENSRNOG00000058068 | Obscn | 0.440273053 | 1.18352955 | 0.000171079 | 0.011934137 |
| ENSRNOG00000014948 | Osgin1 | 0.401407906 | 1.316859063 | 0.001063193 | 0.03822499 |
| ENSRNOG00000004302 | Pah | 2.769192771 | -1.469465487 | 8.74483E-05 | 0.007790625 |
| ENSRNOG00000011310 | Pde10a | 0.064797677 | 3.947914096 | 0.000349664 | 0.018798537 |
| ENSRNOG00000036828 | Pde1b | 0.152225267 | 2.71572025 | 0.000162408 | 0.011510183 |
| ENSRNOG00000019560 | Pde2a | 0.23798195 | 2.071075939 | 0.001639783 | 0.048565171 |
| ENSRNOG00000013436 | Pde7b | 0.135939057 | 2.878968079 | 0.000138594 | 0.010457227 |
| ENSRNOG00000010280 | Pde8b | 0.278319836 | 1.845184359 | 0.001404064 | 0.044879465 |
| ENSRNOG00000049937 | Pdxk | 0.457520763 | 1.128090877 | 0.001145332 | 0.040137298 |
| ENSRNOG00000026036 | Pdyn | 0.123235562 | 3.020509467 | 0.000145673 | 0.010735745 |
| ENSRNOG00000013140 | Pdzd2 | 0.223517876 | 2.161537879 | 0.001134719 | 0.039828285 |
| ENSRNOG00000008943 | Penk | 0.053014081 | 4.237480588 | 9.80531E-05 | 0.008207966 |
| ENSRNOG00000011994 | Perp | 3.917195579 | -1.969821161 | 4.69729E-05 | 0.005322764 |
| ENSRNOG00000000525 | Pi16 | 0.38975184 | 1.35937226 | 2.66237E-07 | 0.000113576 |
| ENSRNOG00000016846 | Pik3cd | 0.367533763 | 1.444051306 | 7.29905E-05 | 0.006919438 |
| ENSRNOG00000011263 | Plac9 | 0.000386546 | 11.33707177 | 0.00101555 | 0.0368828 |
| ENSRNOG00000025587 | Plagl1 | 3.513348825 | -1.812846822 | 6.61582E-07 | 0.000225783 |
| ENSRNOG00000004810 | Plcb1 | 0.260809803 | 1.938929997 | 0.001468264 | 0.045957479 |
| ENSRNOG00000033119 | Plcb4 | 2.542807255 | -1.34642211 | 4.83926E-05 | 0.005421687 |
| ENSRNOG00000024346 | Plet1 | 0.000343063 | 11.50923892 | 7.50868E-06 | 0.001542269 |
| ENSRNOG00000017223 | Plg | 3.713404509 | -1.89274248 | 2.40069E-06 | 0.000641621 |
| ENSRNOG00000018815 | Plk1 | 0.371109389 | 1.430083595 | 0.001037813 | 0.037494805 |
| ENSRNOG00000011951 | Plk2 | 0.183017762 | 2.449944425 | 0.000210406 | 0.013447616 |
| ENSRNOG00000007575 | Plppr1 | 0.198208853 | 2.334906692 | 8.57674E-05 | 0.007702749 |
| ENSRNOG00000007324 | Plxna2 | 0.228737722 | 2.128233791 | 6.98854E-05 | 0.006740297 |
| ENSRNOG00000025209 | Plxnd1 | 0.349215883 | 1.517808917 | 0.001007827 | 0.0368828 |
| ENSRNOG00000013322 | Pola1 | 0.400825399 | 1.318954163 | 0.000275425 | 0.016163377 |
| ENSRNOG00000012686 | Pomc | 11.62001717 | -3.538540296 | 2.26837E-05 | 0.003310472 |
| ENSRNOG00000047686 | Pou3f1 | 0.205625718 | 2.28190738 | 0.000294227 | 0.016911841 |
| ENSRNOG00000028404 | Ppp1r1b | 0.078583523 | 3.669629339 | 3.30989E-06 | 0.00082498 |
| ENSRNOG00000008869 | Ppp1r9a | 0.280548617 | 1.833677296 | 0.000983195 | 0.036835251 |
| ENSRNOG00000009882 | Ppp3ca | 0.2162944 | 2.20893178 | 0.000612537 | 0.026800628 |
| ENSRNOG00000009218 | Prdm12 | 40.92216433 | -5.354810545 | 0.001470938 | 0.045957479 |
| ENSRNOG00000045913 | Prdm16 | 0.197304133 | 2.341506919 | 0.000207823 | 0.013362746 |
| ENSRNOG00000003120 | Prelp | 0.254430352 | 1.97465731 | 4.6029E-07 | 0.000173061 |
| ENSRNOG00000004873 | Prkch | 0.424324778 | 1.236759171 | 0.000131986 | 0.010061337 |
| ENSRNOG00000002361 | Prkg2 | 2.282572125 | -1.190660447 | 1.82886E-05 | 0.002837028 |
| ENSRNOG00000021447 | Prr7 | 0.256137614 | 1.965008965 | 0.001550976 | 0.047206646 |
| ENSRNOG00000019435 | Psd | 0.28052746 | 1.833786097 | 0.001276547 | 0.042699999 |
| ENSRNOG00000057616 | Ptch2 | 2.231018175 | -1.157702267 | 3.4288E-05 | 0.004365375 |
| ENSRNOG00000002525 | Ptgs2 | 0.293833552 | 1.766928952 | 7.893E-08 | 4.07187E-05 |
| ENSRNOG00000027839 | Ptk2b | 0.176841102 | 2.499474466 | 0.000572796 | 0.025758992 |
| ENSRNOG00000013415 | Ptpn18 | 0.459227811 | 1.122718079 | 0.001225789 | 0.042223102 |
| ENSRNOG00000011425 | Ptpn3 | 7.22311392 | -2.852620924 | 0.000674101 | 0.028701679 |
| ENSRNOG00000013981 | Ptpn5 | 0.240599779 | 2.055292776 | 0.001084877 | 0.038721483 |
| ENSRNOG00000005807 | Ptpn7 | 0.299790022 | 1.737975729 | 0.00059317 | 0.026264041 |
| ENSRNOG00000005277 | Ptprv | 0.055545003 | 4.170199068 | 9.19695E-07 | 0.000300024 |
| ENSRNOG00000007364 | Rab15 | 0.327190653 | 1.611796561 | 0.001633518 | 0.048444294 |
| ENSRNOG00000036661 | Rab40b | 0.088391477 | 3.499948915 | 2.1448E-05 | 0.003236604 |
| ENSRNOG00000049070 | Rack1 | 0.174358257 | 2.519873412 | 2.61683E-14 | 7.25615E-11 |
| ENSRNOG00000014420 | Rap2b | 0.434029312 | 1.204135618 | 0.000401086 | 0.020469014 |
| ENSRNOG00000024061 | Rarb | 0.146978508 | 2.766322882 | 3.84394E-05 | 0.004720589 |
| ENSRNOG00000024705 | Rarres2 | 0.412457623 | 1.277682193 | 0.000792821 | 0.031816417 |
| ENSRNOG00000014761 | Rasd2 | 0.084257422 | 3.569052419 | 0.001006298 | 0.0368828 |
| ENSRNOG00000031671 | Rasgef1a | 0.247631605 | 2.013732636 | 0.00134653 | 0.043797753 |
| ENSRNOG00000021098 | Rasgrp2 | 0.23647881 | 2.08021718 | 0.000856768 | 0.033401894 |
| ENSRNOG00000013794 | Rbp1 | 0.389173341 | 1.361515208 | 0.000399161 | 0.020449418 |
| ENSRNOG00000013452 | Rcn1 | 0.364497624 | 1.456018683 | 0.000780212 | 0.031525402 |
| ENSRNOG00000011646 | Rem2 | 0.090271033 | 3.469593069 | 0.000111593 | 0.008936701 |
| ENSRNOG00000027592 | Rerg | 0.272766566 | 1.874261275 | 0.000943039 | 0.03597753 |
| ENSRNOG00000022723 | RGD1562029 | 0.149709182 | 2.739765391 | 3.92244E-05 | 0.004754732 |
| ENSRNOG00000014424 | RGD1563354 | 0.337183982 | 1.568392091 | 3.23331E-05 | 0.004170026 |
| ENSRNOG00000055564 | RGD1564664 | 0.232146094 | 2.106895089 | 1.13153E-05 | 0.001994262 |
| ENSRNOG00000007949 | Rgn | 4.153494016 | -2.054325476 | 9.41949E-09 | 6.3319E-06 |
| ENSRNOG00000015616 | Rgs14 | 0.082823829 | 3.593810282 | 5.02918E-05 | 0.00557811 |
| ENSRNOG00000003800 | Rgs9 | 0.040956877 | 4.60975049 | 0.00015204 | 0.011058008 |
| ENSRNOG00000050223 | Rin1 | 0.0355088 | 4.815679601 | 7.15417E-05 | 0.006815146 |
| ENSRNOG00000055721 | Rnf128 | 2.130680993 | -1.091314608 | 2.20601E-06 | 0.00059678 |
| ENSRNOG00000003479 | Rnf150 | 0.223627179 | 2.160832556 | 0.001339039 | 0.043618068 |
| ENSRNOG00000042781 | Ropn1l | 0.194290363 | 2.363713751 | 0.001619234 | 0.048149417 |
| ENSRNOG00000005109 | Rprm | 3.665587371 | -1.874044393 | 0.000773223 | 0.031357223 |
| ENSRNOG00000028436 | Rprml | 0.139698071 | 2.839616 | 0.000227903 | 0.014161267 |
| ENSRNOG00000004362 | Rps6ka5 | 0.340561867 | 1.554011192 | 0.00036164 | 0.019135069 |
| ENSRNOG00000015701 | Rreb1 | 0.425056608 | 1.234273105 | 0.001616312 | 0.048127044 |
| ENSRNOG00000007539 | Rsad2 | 0.384122246 | 1.380362577 | 0.000603291 | 0.026510106 |
| ENSRNOG00000057862 | Rsph1 | 0.187730896 | 2.413261989 | 3.50543E-07 | 0.000143994 |
| ENSRNOG00000049696 | Rsph4a | 0.098954901 | 3.33708503 | 0.000357017 | 0.018992095 |
| ENSRNOG00000061526 | Rsph6a | 2.463098116 | -1.300474098 | 0.000153792 | 0.011132153 |
| ENSRNOG00000033215 | RT1-Db1 | 0.070137939 | 3.83366116 | 0.001292014 | 0.042777235 |
| ENSRNOG00000004537 | Rxrg | 0.14903042 | 2.746321253 | 0.000242613 | 0.01471685 |
| ENSRNOG00000020901 | S1pr5 | 2.090433314 | -1.063802021 | 0.001616293 | 0.048127044 |
| ENSRNOG00000003809 | Sat1 | 0.321349662 | 1.63778414 | 2.25779E-05 | 0.003310472 |
| ENSRNOG00000016177 | Scara3 | 0.337221041 | 1.568233538 | 1.65132E-05 | 0.002686307 |
| ENSRNOG00000032554 | Scd4 | 0.000237406 | 12.04035793 | 2.97128E-05 | 0.003900118 |
| ENSRNOG00000015055 | Scg2 | 2.378114756 | -1.249818334 | 1.65904E-05 | 0.002686307 |
| ENSRNOG00000026679 | Scn4b | 0.145218286 | 2.783704961 | 2.43488E-05 | 0.003440697 |
| ENSRNOG00000023337 | Sema3a | 0.094086247 | 3.409872337 | 3.96942E-07 | 0.000157239 |
| ENSRNOG00000001865 | Serpind1 | 5.895680017 | -2.559658226 | 2.57903E-06 | 0.000673065 |
| ENSRNOG00000016208 | Setbp1 | 0.350995668 | 1.51047487 | 0.001244693 | 0.042609593 |
| ENSRNOG00000017783 | Sfrp1 | 0.140558181 | 2.830760665 | 1.0908E-05 | 0.001980789 |
| ENSRNOG00000006263 | Sh2d1a | 2.272795745 | -1.184468036 | 0.000253025 | 0.01525229 |
| ENSRNOG00000018780 | Sh3rf2 | 0.024436229 | 5.354834543 | 8.52419E-08 | 4.29755E-05 |
| ENSRNOG00000028685 | Shf | 0.486331395 | 1.039988366 | 0.000528959 | 0.024341449 |
| ENSRNOG00000012521 | Shisa2 | 0.303573397 | 1.719882727 | 0.000769429 | 0.031317889 |
| ENSRNOG00000016877 | Shisa7 | 0.218370831 | 2.195147931 | 0.001426323 | 0.045394724 |
| ENSRNOG00000049407 | Shisa8 | 0.271213499 | 1.882499108 | 0.00073121 | 0.030149485 |
| ENSRNOG00000012478 | Shox2 | 19.97153609 | -4.319873396 | 1.14031E-06 | 0.000347324 |
| ENSRNOG00000007646 | Sipa1l1 | 0.280400176 | 1.834440838 | 0.000492 | 0.023420661 |
| ENSRNOG00000055340 | Ski | 0.45760968 | 1.127810524 | 0.000309409 | 0.017332371 |
| ENSRNOG00000009899 | Skil | 0.460503662 | 1.118715466 | 0.001333072 | 0.043530642 |
| ENSRNOG00000026091 | Slc10a4 | 2.653195765 | -1.407731128 | 0.000515531 | 0.024025265 |
| ENSRNOG00000011184 | Slc13a4 | 0.144577745 | 2.790082603 | 0.001542444 | 0.047129528 |
| ENSRNOG00000011598 | Slc15a1 | 0.000761058 | 10.35970564 | 0.000278626 | 0.016251984 |
| ENSRNOG00000012090 | Slc16a8 | 0.07250611 | 3.785753622 | 0.000191781 | 0.012775624 |
| ENSRNOG00000007581 | Slc17a8 | 0.053575664 | 4.222278363 | 8.36272E-06 | 0.001686456 |
| ENSRNOG00000062141 | Slc18a3 | 0.051250084 | 4.286301825 | 1.67468E-07 | 7.58151E-05 |
| ENSRNOG00000005479 | Slc1a2 | 0.349294942 | 1.517482343 | 0.00023358 | 0.014473495 |
| ENSRNOG00000060687 | Slc24a3 | 2.143046448 | -1.099663119 | 3.69214E-06 | 0.000882256 |
| ENSRNOG00000006729 | Slc24a4 | 0.153575068 | 2.702984071 | 0.000239235 | 0.014660113 |
| ENSRNOG00000009832 | Slc39a14 | 0.310401609 | 1.687792057 | 0.000364371 | 0.019199149 |
| ENSRNOG00000012508 | Slc39a8 | 0.310348909 | 1.688037021 | 1.53351E-09 | 1.36072E-06 |
| ENSRNOG00000021234 | Slc4a11 | 0.082345173 | 3.602172114 | 1.68116E-05 | 0.0027024 |
| ENSRNOG00000010597 | Slc5a7 | 0.121822466 | 3.037147879 | 6.44054E-08 | 3.40222E-05 |
| ENSRNOG00000006010 | Slc6a20 | 0.405218371 | 1.303228512 | 0.000756343 | 0.030955637 |
| ENSRNOG00000010210 | Slc7a11 | 0.305411337 | 1.71117448 | 0.001204572 | 0.041686461 |
| ENSRNOG00000007377 | Slit3 | 0.189758461 | 2.397763879 | 8.86834E-05 | 0.007869053 |
| ENSRNOG00000011339 | Slk | 0.415363099 | 1.267555043 | 0.001297344 | 0.042843587 |
| ENSRNOG00000008620 | Smad3 | 0.134023417 | 2.899443004 | 9.34397E-07 | 0.000300402 |
| ENSRNOG00000019536 | Smim3 | 0.465706112 | 1.102508279 | 0.000943918 | 0.03597753 |
| ENSRNOG00000000257 | Smpd3 | 0.168312429 | 2.570786379 | 0.000123306 | 0.009597569 |
| ENSRNOG00000042326 | Smpdl3b | 0.174700058 | 2.517048011 | 0.000207265 | 0.013362746 |
| ENSRNOG00000004928 | Sntg2 | 0.318022239 | 1.652800438 | 0.000940393 | 0.035966781 |
| ENSRNOG00000005770 | Sostdc1 | 0.005912978 | 7.401899312 | 9.87743E-05 | 0.008237256 |
| ENSRNOG00000060061 | Sowaha | 0.13440903 | 2.895298032 | 9.4065E-05 | 0.008121496 |
| ENSRNOG00000022800 | Sp140 | 0.427376044 | 1.226422053 | 0.001490263 | 0.046300424 |
| ENSRNOG00000025787 | Spag6l | 0.137628472 | 2.861149131 | 0.001558078 | 0.047217001 |
| ENSRNOG00000032539 | Spag8 | 0.35380465 | 1.498975087 | 0.00126621 | 0.042622239 |
| ENSRNOG00000013707 | Spata13 | 0.419856781 | 1.252030807 | 4.2504E-05 | 0.004962449 |
| ENSRNOG00000016388 | Sphkap | 2.334202242 | -1.222929566 | 1.57742E-10 | 1.94399E-07 |
| ENSRNOG00000024028 | Sprr1a | 0.000166986 | 12.54798138 | 3.10119E-05 | 0.004023024 |
| ENSRNOG00000058842 | Sptbn2 | 0.270567249 | 1.885940875 | 0.00140016 | 0.044879465 |
| ENSRNOG00000025848 | Sspo | 0.304809489 | 1.714020277 | 0.000294278 | 0.016911841 |
| ENSRNOG00000015075 | Stc1 | 0.30349235 | 1.720267944 | 0.000170069 | 0.011901088 |
| ENSRNOG00000000017 | Steap1 | 0.049320106 | 4.341680291 | 0.000480856 | 0.022939427 |
| ENSRNOG00000007879 | Stk26 | 0.253741194 | 1.97857034 | 0.000271685 | 0.016087293 |
| ENSRNOG00000031397 | Stk32b | 3.036991555 | -1.602642897 | 1.50755E-05 | 0.00247718 |
| ENSRNOG00000004458 | Ston2 | 0.385297939 | 1.375953626 | 0.000212777 | 0.013485824 |
| ENSRNOG00000023712 | Stox1 | 0.104394594 | 3.259881091 | 0.00015715 | 0.011281749 |
| ENSRNOG00000008312 | Stra6 | 0.076106287 | 3.715840551 | 1.50945E-06 | 0.000440581 |
| ENSRNOG00000037960 | Stum | 0.124783905 | 3.002496237 | 9.48234E-05 | 0.008121496 |
| ENSRNOG00000009037 | Sulf1 | 0.297129714 | 1.75083521 | 1.0267E-05 | 0.001915648 |
| ENSRNOG00000019181 | Synpo | 0.196812138 | 2.345108898 | 0.000198938 | 0.013017827 |
| ENSRNOG00000008203 | Synpr | 0.168963748 | 2.565214352 | 0.000120682 | 0.009426391 |
| ENSRNOG00000014296 | Syt10 | 0.084929942 | 3.55758292 | 0.000127532 | 0.009789098 |
| ENSRNOG00000019163 | Syt6 | 0.252233449 | 1.987168491 | 0.000308647 | 0.017332371 |
| ENSRNOG00000007374 | Tac1 | 0.113790687 | 3.135545604 | 2.43331E-05 | 0.003440697 |
| ENSRNOG00000004229 | Tac3 | 0.033317695 | 4.907567605 | 3.28155E-09 | 2.5998E-06 |
| ENSRNOG00000021510 | Tbc1d10c | 0.076221623 | 3.713655867 | 7.29136E-06 | 0.001511628 |
| ENSRNOG00000049758 | Tbc1d16 | 0.251509521 | 1.991315079 | 0.000984687 | 0.036835251 |
| ENSRNOG00000049232 | Tcf7l2 | 9.041256565 | -3.176523294 | 1.51168E-10 | 1.94399E-07 |
| ENSRNOG00000015488 | Tead1 | 0.44942523 | 1.153846977 | 0.000418465 | 0.020860261 |
| ENSRNOG00000002278 | Tec | 0.431941468 | 1.211092268 | 4.26563E-06 | 0.000985671 |
| ENSRNOG00000001128 | Tesc | 0.181359211 | 2.463078077 | 0.000421418 | 0.020911593 |
| ENSRNOG00000030625 | Tf | 0.302927847 | 1.72295389 | 0.001353426 | 0.043829253 |
| ENSRNOG00000006753 | Tgm3 | 0.000378273 | 11.36828455 | 5.91213E-06 | 0.001261045 |
| ENSRNOG00000004687 | Thbd | 0.480135877 | 1.058485353 | 0.000381691 | 0.019736698 |
| ENSRNOG00000016728 | Tiam2 | 0.145901633 | 2.77693206 | 1.78149E-05 | 0.002822771 |
| ENSRNOG00000005100 | Tmco5a | 0.064283445 | 3.95940894 | 0.000118735 | 0.009349802 |
| ENSRNOG00000046851 | Tmem121b | 0.166700066 | 2.584673423 | 0.00126291 | 0.042622239 |
| ENSRNOG00000004757 | Tmem158 | 0.148493704 | 2.751526335 | 0.000414048 | 0.020780173 |
| ENSRNOG00000032018 | Tmem200b | 0.058540209 | 4.094428282 | 3.38105E-05 | 0.004335371 |
| ENSRNOG00000024259 | Tmem54 | 0.100198455 | 3.319067835 | 3.88356E-05 | 0.004733458 |
| ENSRNOG00000023340 | Tmem72 | 0.020439138 | 5.612521828 | 0.001486126 | 0.046300424 |
| ENSRNOG00000010165 | Tnfaip2 | 0.193902847 | 2.366594107 | 3.08301E-06 | 0.000777163 |
| ENSRNOG00000024230 | Tnfaip8l3 | 0.252953035 | 1.983058548 | 0.000307526 | 0.017332371 |
| ENSRNOG00000020276 | Tnni2 | 0.024908049 | 5.327244163 | 5.4618E-06 | 0.001199596 |
| ENSRNOG00000018250 | Tnni3 | 0.113818899 | 3.135187967 | 0.001234859 | 0.042365013 |
| ENSRNOG00000020332 | Tnnt3 | 0.089964487 | 3.474500574 | 2.39702E-25 | 1.77243E-21 |
| ENSRNOG00000001640 | Tomm70 | 0.31728999 | 1.656126087 | 0.001552798 | 0.047206646 |
| ENSRNOG00000053047 | Top2a | 0.209290054 | 2.256424339 | 0.000334581 | 0.018191179 |
| ENSRNOG00000010777 | Tox | 2.281056736 | -1.189702331 | 7.41901E-05 | 0.00694413 |
| ENSRNOG00000008146 | Tox2 | 2.88834942 | -1.530245284 | 0.000677854 | 0.028806193 |
| ENSRNOG00000008738 | Tp53i11 | 2.280721505 | -1.189490293 | 0.001462398 | 0.045957479 |
| ENSRNOG00000024707 | Tp73 | 0.061615113 | 4.020571936 | 0.000633209 | 0.027381057 |
| ENSRNOG00000030101 | Traip | 0.10739351 | 3.219021282 | 0.000110631 | 0.008924074 |
| ENSRNOG00000021091 | Trank1 | 0.209734174 | 2.253366141 | 0.000870324 | 0.033870861 |
| ENSRNOG00000011824 | Trh | 0.150078486 | 2.73621092 | 9.71547E-08 | 4.7893E-05 |
| ENSRNOG00000002341 | Trim25 | 0.453245421 | 1.141635649 | 0.000915765 | 0.035329403 |
| ENSRNOG00000021771 | Trim29 | 29.60297747 | -4.887670385 | 8.48535E-05 | 0.00765165 |
| ENSRNOG00000048580 | Trip6 | 0.475283721 | 1.073139107 | 2.72038E-09 | 2.23504E-06 |
| ENSRNOG00000011133 | Trpc4 | 2.130935814 | -1.091487138 | 0.000900021 | 0.034904117 |
| ENSRNOG00000010549 | Tspo | 0.262674874 | 1.928649889 | 0.000226325 | 0.014142461 |
| ENSRNOG00000033901 | Ttll2 | 0.000216556 | 12.17297331 | 2.4886E-07 | 0.000108244 |
| ENSRNOG00000016275 | Ttr | 0.004489437 | 7.799249677 | 8.35513E-05 | 0.007564972 |
| ENSRNOG00000047027 | Unc93a | 0.000662525 | 10.55973716 | 0.001603873 | 0.048079348 |
| ENSRNOG00000016339 | Uox | 11.13103524 | -3.476515872 | 0.000184243 | 0.01252471 |
| ENSRNOG00000043404 | Uroc1 | 1075.636667 | -10.07097512 | 9.00342E-05 | 0.007933846 |
| ENSRNOG00000048378 | Vrtn | 0.000720404 | 10.43890618 | 0.000851945 | 0.03333102 |
| ENSRNOG00000025110 | Vwa3a | 0.11114878 | 3.169435979 | 1.02764E-05 | 0.001915648 |
| ENSRNOG00000027400 | Vwc2l | 2.727330248 | -1.447489404 | 0.000376223 | 0.019628224 |
| ENSRNOG00000048847 | Wdr17 | 0.18381902 | 2.443642043 | 0.000380662 | 0.019729514 |
| ENSRNOG00000014739 | Wfdc2 | 0.206712313 | 2.274303771 | 0.001004267 | 0.0368828 |
| ENSRNOG00000005108 | Wfs1 | 0.140992552 | 2.826309138 | 2.59826E-05 | 0.003557846 |
| ENSRNOG00000020441 | Wnk4 | 0.317010915 | 1.65739558 | 0.001360563 | 0.043932137 |
| ENSRNOG00000052510 | Wnt10a | 0.104243551 | 3.261969961 | 0.000999998 | 0.0368828 |
| ENSRNOG00000014385 | Wnt2b | 4.80042326 | -2.263161616 | 0.000190474 | 0.012726762 |
| ENSRNOG00000006972 | Zfp189 | 0.37166158 | 1.427938536 | 0.000157987 | 0.011305239 |
| ENSRNOG00000019065 | Zfp385b | 0.346495663 | 1.529090802 | 0.000794592 | 0.03181665 |
| ENSRNOG00000024693 | Zfp831 | 0.055373989 | 4.174647727 | 1.38766E-05 | 0.002349815 |
| ENSRNOG00000042101 | Zfp93 | 0.228621834 | 2.128964904 | 0.001504712 | 0.046502455 |
| ENSRNOG00000019477 | Zmynd15 | 0.383911932 | 1.381152695 | 0.000124838 | 0.009650593 |
| ENSRNOG00000004426 | Rps27a | 2.168826775 | 1.116914829 | 1.63586E-07 | 7.56006E-05 |
| ENSRNOG00000004048 | Lrrk2 | 0.148912199 | -2.747466152 | 6.04507E-05 | 0.006218622 |
| ENSRNOG00000002229 | Adcy5 | 0.154725492 | -2.69221719 | 6.29658E-05 | 0.006348958 |
| ENSRNOG00000023688 | Drd1 | 0.035350766 | -4.822114729 | 9.47397E-05 | 0.008121496 |
| ENSRNOG00000001302 | Adora2a | 0.044294841 | -4.496717519 | 0.000105043 | 0.008598401 |
| ENSRNOG00000007104 | Itpr1 | 0.171561612 | -2.543201318 | 0.000508182 | 0.023841984 |
| ENSRNOG00000010440 | Gnal | 0.27154858 | -1.880717773 | 0.000992812 | 0.0368828 |
| ENSRNOG00000038202 | Calml4 | 0.112078729 | -3.157415593 | 0.001020513 | 0.036990253 |

| **GeneID** | **Gene_Name** | **Fold Change** | **log2(fc)** | **pval** | **qval** |
| --- | --- | --- | --- | --- | --- |
| ENSRNOG00000006972 | Zfp189 | 0.37 | -1.43 | 0.00 | 0.01 |
| ENSRNOG00000014385 | Wnt2b | 4.80 | 2.26 | 0.00 | 0.01 |
| ENSRNOG00000052510 | Wnt10a | 0.10 | -3.26 | 0.00 | 0.04 |
| ENSRNOG00000020441 | Wnk4 | 0.32 | -1.66 | 0.00 | 0.04 |
| ENSRNOG00000005108 | Wfs1 | 0.14 | -2.83 | 0.00 | 0.00 |
| ENSRNOG00000048847 | Wdr17 | 0.18 | -2.44 | 0.00 | 0.02 |
| ENSRNOG00000025110 | Vwa3a | 0.11 | -3.17 | 0.00 | 0.00 |
| ENSRNOG00000047027 | Unc93a | 0.00 | -10.56 | 0.00 | 0.05 |
| ENSRNOG00000011824 | Trh | 0.15 | -2.74 | 0.00 | 0.00 |
| ENSRNOG00000021091 | Trank1 | 0.21 | -2.25 | 0.00 | 0.03 |
| ENSRNOG00000030101 | Traip | 0.11 | -3.22 | 0.00 | 0.01 |
| ENSRNOG00000008146 | Tox2 | 2.89 | 1.53 | 0.00 | 0.03 |
| ENSRNOG00000010777 | Tox | 2.28 | 1.19 | 0.00 | 0.01 |
| ENSRNOG00000020332 | Tnnt3 | 0.09 | -3.47 | 0.00 | 0.00 |
| ENSRNOG00000018250 | Tnni3 | 0.11 | -3.14 | 0.00 | 0.04 |
| ENSRNOG00000010165 | Tnfaip2 | 0.19 | -2.37 | 0.00 | 0.00 |
| ENSRNOG00000024259 | Tmem54 | 0.10 | -3.32 | 0.00 | 0.00 |
| ENSRNOG00000032018 | Tmem200b | 0.06 | -4.09 | 0.00 | 0.00 |
| ENSRNOG00000046851 | Tmem121b | 0.17 | -2.58 | 0.00 | 0.04 |
| ENSRNOG00000016728 | Tiam2 | 0.15 | -2.78 | 0.00 | 0.00 |
| ENSRNOG00000001128 | Tesc | 0.18 | -2.46 | 0.00 | 0.02 |
| ENSRNOG00000049758 | Tbc1d16 | 0.25 | -1.99 | 0.00 | 0.04 |
| ENSRNOG00000021510 | Tbc1d10c | 0.08 | -3.71 | 0.00 | 0.00 |
| ENSRNOG00000004229 | Tac3 | 0.03 | -4.91 | 0.00 | 0.00 |
| ENSRNOG00000007374 | Tac1 | 0.11 | -3.14 | 0.00 | 0.00 |
| ENSRNOG00000019163 | Syt6 | 0.25 | -1.99 | 0.00 | 0.02 |
| ENSRNOG00000014296 | Syt10 | 0.08 | -3.56 | 0.00 | 0.01 |
| ENSRNOG00000008203 | Synpr | 0.17 | -2.57 | 0.00 | 0.01 |
| ENSRNOG00000019181 | Synpo | 0.20 | -2.35 | 0.00 | 0.01 |
| ENSRNOG00000009037 | Sulf1 | 0.30 | -1.75 | 0.00 | 0.00 |
| ENSRNOG00000037960 | Stum | 0.12 | -3.00 | 0.00 | 0.01 |
| ENSRNOG00000008312 | Stra6 | 0.08 | -3.72 | 0.00 | 0.00 |
| ENSRNOG00000023712 | Stox1 | 0.10 | -3.26 | 0.00 | 0.01 |
| ENSRNOG00000007879 | Stk26 | 0.25 | -1.98 | 0.00 | 0.02 |
| ENSRNOG00000058842 | Sptbn2 | 0.27 | -1.89 | 0.00 | 0.04 |
| ENSRNOG00000024028 | Sprr1a | 0.00 | -12.55 | 0.00 | 0.00 |
| ENSRNOG00000016388 | Sphkap | 2.33 | 1.22 | 0.00 | 0.00 |
| ENSRNOG00000060061 | Sowaha | 0.13 | -2.90 | 0.00 | 0.01 |
| ENSRNOG00000005770 | Sostdc1 | 0.01 | -7.40 | 0.00 | 0.01 |
| ENSRNOG00000042326 | Smpdl3b | 0.17 | -2.52 | 0.00 | 0.01 |
| ENSRNOG00000000257 | Smpd3 | 0.17 | -2.57 | 0.00 | 0.01 |
| ENSRNOG00000008620 | Smad3 | 0.13 | -2.90 | 0.00 | 0.00 |
| ENSRNOG00000007377 | Slit3 | 0.19 | -2.40 | 0.00 | 0.01 |
| ENSRNOG00000010597 | Slc5a7 | 0.12 | -3.04 | 0.00 | 0.00 |
| ENSRNOG00000021234 | Slc4a11 | 0.08 | -3.60 | 0.00 | 0.00 |
| ENSRNOG00000006729 | Slc24a4 | 0.15 | -2.70 | 0.00 | 0.01 |
| ENSRNOG00000060687 | Slc24a3 | 2.14 | 1.10 | 0.00 | 0.00 |
| ENSRNOG00000005479 | Slc1a2 | 0.35 | -1.52 | 0.00 | 0.01 |
| ENSRNOG00000062141 | Slc18a3 | 0.05 | -4.29 | 0.00 | 0.00 |
| ENSRNOG00000007581 | Slc17a8 | 0.05 | -4.22 | 0.00 | 0.00 |
| ENSRNOG00000026091 | Slc10a4 | 2.65 | 1.41 | 0.00 | 0.02 |
| ENSRNOG00000007646 | Sipa1l1 | 0.28 | -1.83 | 0.00 | 0.02 |
| ENSRNOG00000016877 | Shisa7 | 0.22 | -2.20 | 0.00 | 0.05 |
| ENSRNOG00000018780 | Sh3rf2 | 0.02 | -5.35 | 0.00 | 0.00 |
| ENSRNOG00000006263 | Sh2d1a | 2.27 | 1.18 | 0.00 | 0.02 |
| ENSRNOG00000017783 | Sfrp1 | 0.14 | -2.83 | 0.00 | 0.00 |
| ENSRNOG00000023337 | Sema3a | 0.09 | -3.41 | 0.00 | 0.00 |
| ENSRNOG00000026679 | Scn4b | 0.15 | -2.78 | 0.00 | 0.00 |
| ENSRNOG00000015055 | Scg2 | 2.38 | 1.25 | 0.00 | 0.00 |
| ENSRNOG00000032554 | Scd4 | 0.00 | -12.04 | 0.00 | 0.00 |
| ENSRNOG00000016177 | Scara3 | 0.34 | -1.57 | 0.00 | 0.00 |
| ENSRNOG00000033215 | RT1-Db1 | 0.07 | -3.83 | 0.00 | 0.04 |
| ENSRNOG00000061526 | Rsph6a | 2.46 | 1.30 | 0.00 | 0.01 |
| ENSRNOG00000015701 | Rreb1 | 0.43 | -1.23 | 0.00 | 0.05 |
| ENSRNOG00000004362 | Rps6ka5 | 0.34 | -1.55 | 0.00 | 0.02 |
| ENSRNOG00000028436 | Rprml | 0.14 | -2.84 | 0.00 | 0.01 |
| ENSRNOG00000003479 | Rnf150 | 0.22 | -2.16 | 0.00 | 0.04 |
| ENSRNOG00000050223 | Rin1 | 0.04 | -4.82 | 0.00 | 0.01 |
| ENSRNOG00000003800 | Rgs9 | 0.04 | -4.61 | 0.00 | 0.01 |
| ENSRNOG00000015616 | Rgs14 | 0.08 | -3.59 | 0.00 | 0.01 |
| ENSRNOG00000007949 | Rgn | 4.15 | 2.05 | 0.00 | 0.00 |
| ENSRNOG00000055564 | RGD1564664 | 0.23 | -2.11 | 0.00 | 0.00 |
| ENSRNOG00000014424 | RGD1563354 | 0.34 | -1.57 | 0.00 | 0.00 |
| ENSRNOG00000011646 | Rem2 | 0.09 | -3.47 | 0.00 | 0.01 |
| ENSRNOG00000002097 | Rasl11b | 0.32 | -1.64 | 0.00 | 0.00 |
| ENSRNOG00000021098 | Rasgrp2 | 0.24 | -2.08 | 0.00 | 0.03 |
| ENSRNOG00000031671 | Rasgef1a | 0.25 | -2.01 | 0.00 | 0.04 |
| ENSRNOG00000014761 | Rasd2 | 0.08 | -3.57 | 0.00 | 0.04 |
| ENSRNOG00000024705 | Rarres2 | 0.41 | -1.28 | 0.00 | 0.03 |
| ENSRNOG00000024061 | Rarb | 0.15 | -2.77 | 0.00 | 0.00 |
| ENSRNOG00000049070 | Rack1 | 0.17 | -2.52 | 0.00 | 0.00 |
| ENSRNOG00000036661 | Rab40b | 0.09 | -3.50 | 0.00 | 0.00 |
| ENSRNOG00000007364 | Rab15 | 0.33 | -1.61 | 0.00 | 0.05 |
| ENSRNOG00000005277 | Ptprv | 0.06 | -4.17 | 0.00 | 0.00 |
| ENSRNOG00000005807 | Ptpn7 | 0.30 | -1.74 | 0.00 | 0.03 |
| ENSRNOG00000013981 | Ptpn5 | 0.24 | -2.06 | 0.00 | 0.04 |
| ENSRNOG00000002525 | Ptgs2 | 0.29 | -1.77 | 0.00 | 0.00 |
| ENSRNOG00000019435 | Psd | 0.28 | -1.83 | 0.00 | 0.04 |
| ENSRNOG00000004873 | Prkch | 0.42 | -1.24 | 0.00 | 0.01 |
| ENSRNOG00000003120 | Prelp | 0.25 | -1.97 | 0.00 | 0.00 |
| ENSRNOG00000045913 | Prdm16 | 0.20 | -2.34 | 0.00 | 0.01 |
| ENSRNOG00000009882 | Ppp3ca | 0.22 | -2.21 | 0.00 | 0.03 |
| ENSRNOG00000028404 | Ppp1r1b | 0.08 | -3.67 | 0.00 | 0.00 |
| ENSRNOG00000047686 | Pou3f1 | 0.21 | -2.28 | 0.00 | 0.02 |
| ENSRNOG00000013322 | Pola1 | 0.40 | -1.32 | 0.00 | 0.02 |
| ENSRNOG00000007324 | Plxna2 | 0.23 | -2.13 | 0.00 | 0.01 |
| ENSRNOG00000007575 | Plppr1 | 0.20 | -2.33 | 0.00 | 0.01 |
| ENSRNOG00000011951 | Plk2 | 0.18 | -2.45 | 0.00 | 0.01 |
| ENSRNOG00000033119 | Plcb4 | 2.54 | 1.35 | 0.00 | 0.01 |
| ENSRNOG00000004810 | Plcb1 | 0.26 | -1.94 | 0.00 | 0.05 |
| ENSRNOG00000011263 | Plac9 | 0.00 | -11.34 | 0.00 | 0.04 |
| ENSRNOG00000016846 | Pik3cd | 0.37 | -1.44 | 0.00 | 0.01 |
| ENSRNOG00000000525 | Pi16 | 0.39 | -1.36 | 0.00 | 0.00 |
| ENSRNOG00000008943 | Penk | 0.05 | -4.24 | 0.00 | 0.01 |
| ENSRNOG00000026036 | Pdyn | 0.12 | -3.02 | 0.00 | 0.01 |
| ENSRNOG00000010280 | Pde8b | 0.28 | -1.85 | 0.00 | 0.04 |
| ENSRNOG00000013436 | Pde7b | 0.14 | -2.88 | 0.00 | 0.01 |
| ENSRNOG00000019560 | Pde2a | 0.24 | -2.07 | 0.00 | 0.05 |
| ENSRNOG00000011310 | Pde10a | 0.06 | -3.95 | 0.00 | 0.02 |
| ENSRNOG00000009243 | Oaf | 0.40 | -1.33 | 0.00 | 0.00 |
| ENSRNOG00000015863 | Npsr1 | 4.54 | 2.18 | 0.00 | 0.01 |
| ENSRNOG00000016653 | Ngef | 0.09 | -3.53 | 0.00 | 0.04 |
| ENSRNOG00000027606 | Neurl1b | 0.12 | -3.07 | 0.00 | 0.00 |
| ENSRNOG00000014006 | Neto1 | 0.13 | -2.98 | 0.00 | 0.01 |
| ENSRNOG00000008415 | Nab2 | 0.28 | -1.82 | 0.00 | 0.02 |
| ENSRNOG00000008356 | Myo5c | 0.04 | -4.51 | 0.00 | 0.01 |
| ENSRNOG00000016983 | Myh7 | 0.29 | -1.77 | 0.00 | 0.03 |
| ENSRNOG00000049695 | Myh4 | 0.07 | -3.94 | 0.00 | 0.00 |
| ENSRNOG00000004500 | Myc | 0.45 | -1.15 | 0.00 | 0.01 |
| ENSRNOG00000019627 | Mybpc2 | 0.13 | -2.95 | 0.00 | 0.00 |
| ENSRNOG00000056817 | Muc6 | 0.13 | -2.93 | 0.00 | 0.02 |
| ENSRNOG00000003171 | Mpz | 0.02 | -5.40 | 0.00 | 0.02 |
| ENSRNOG00000012946 | Mov10 | 0.39 | -1.34 | 0.00 | 0.02 |
| ENSRNOG00000009514 | Mme | 0.24 | -2.04 | 0.00 | 0.02 |
| ENSRNOG00000005934 | Mlip | 0.22 | -2.21 | 0.00 | 0.03 |
| ENSRNOG00000006588 | Meox2 | 0.08 | -3.63 | 0.00 | 0.04 |
| ENSRNOG00000004730 | Meis2 | 0.09 | -3.42 | 0.00 | 0.00 |
| ENSRNOG00000053787 | Mdfic | 0.26 | -1.92 | 0.00 | 0.00 |
| ENSRNOG00000013282 | Mctp1 | 0.09 | -3.51 | 0.00 | 0.00 |
| ENSRNOG00000003703 | Mcm6 | 0.40 | -1.31 | 0.00 | 0.01 |
| ENSRNOG00000015401 | Mapk4 | 0.21 | -2.25 | 0.00 | 0.01 |
| ENSRNOG00000000800 | Man1a1 | 0.30 | -1.75 | 0.00 | 0.01 |
| ENSRNOG00000021231 | Lzts3 | 0.22 | -2.17 | 0.00 | 0.03 |
| ENSRNOG00000011826 | Lzts1 | 0.18 | -2.47 | 0.00 | 0.02 |
| ENSRNOG00000004048 | Lrrk2 | 0.15 | -2.75 | 0.00 | 0.01 |
| ENSRNOG00000030180 | Lrrc10b | 0.05 | -4.40 | 0.00 | 0.02 |
| ENSRNOG00000012181 | Lpl | 0.21 | -2.23 | 0.00 | 0.03 |
| ENSRNOG00000008680 | Loxl1 | 0.28 | -1.86 | 0.00 | 0.01 |
| ENSRNOG00000020133 | LOC108348044 | 0.03 | -4.93 | 0.00 | 0.00 |
| ENSRNOG00000023086 | LOC100360828 | 0.30 | -1.72 | 0.00 | 0.04 |
| ENSRNOG00000060775 | Lmo7 | 0.12 | -3.05 | 0.00 | 0.01 |
| ENSRNOG00000025448 | Limd2 | 0.50 | -1.00 | 0.00 | 0.02 |
| ENSRNOG00000016879 | Ldlrad4 | 0.47 | -1.08 | 0.00 | 0.01 |
| ENSRNOG00000005457 | Lamp5 | 0.15 | -2.76 | 0.00 | 0.01 |
| ENSRNOG00000006025 | Lamb3 | 2.16 | 1.11 | 0.00 | 0.01 |
| ENSRNOG00000049495 | Krt71 | 0.15 | -2.77 | 0.00 | 0.02 |
| ENSRNOG00000051487 | Kremen1 | 0.13 | -2.91 | 0.00 | 0.00 |
| ENSRNOG00000024479 | Klhl34 | 0.17 | -2.55 | 0.00 | 0.01 |
| ENSRNOG00000029441 | Klhl2 | 0.30 | -1.74 | 0.00 | 0.04 |
| ENSRNOG00000008785 | Klf5 | 0.10 | -3.34 | 0.00 | 0.00 |
| ENSRNOG00000033694 | Klf16 | 0.25 | -2.02 | 0.00 | 0.02 |
| ENSRNOG00000016467 | Kctd1 | 0.32 | -1.65 | 0.00 | 0.05 |
| ENSRNOG00000004117 | Kcnv1 | 0.12 | -3.10 | 0.00 | 0.00 |
| ENSRNOG00000011369 | Kcns2 | 0.09 | -3.54 | 0.00 | 0.00 |
| ENSRNOG00000013781 | Kcnq5 | 0.14 | -2.82 | 0.00 | 0.01 |
| ENSRNOG00000002653 | Kcnk2 | 0.21 | -2.28 | 0.00 | 0.01 |
| ENSRNOG00000013869 | Kcnj4 | 0.03 | -5.10 | 0.00 | 0.00 |
| ENSRNOG00000018790 | Kcnh4 | 0.12 | -3.10 | 0.00 | 0.01 |
| ENSRNOG00000057315 | Kcnh3 | 0.05 | -4.45 | 0.00 | 0.01 |
| ENSRNOG00000003841 | Kcnh1 | 0.21 | -2.27 | 0.00 | 0.01 |
| ENSRNOG00000054314 | Kcng1 | 0.17 | -2.57 | 0.00 | 0.03 |
| ENSRNOG00000024310 | Kcnf1 | 0.13 | -3.00 | 0.00 | 0.01 |
| ENSRNOG00000056697 | Kcnab1 | 0.18 | -2.50 | 0.00 | 0.01 |
| ENSRNOG00000019719 | Kcna5 | 0.12 | -3.04 | 0.00 | 0.00 |
| ENSRNOG00000042838 | Junb | 0.34 | -1.54 | 0.00 | 0.00 |
| ENSRNOG00000007104 | Itpr1 | 0.17 | -2.54 | 0.00 | 0.02 |
| ENSRNOG00000005284 | Itpka | 0.05 | -4.20 | 0.00 | 0.02 |
| ENSRNOG00000006860 | Itk | 0.07 | -3.75 | 0.00 | 0.01 |
| ENSRNOG00000022071 | Itga2b | 0.16 | -2.64 | 0.00 | 0.01 |
| ENSRNOG00000006723 | Itga11 | 0.27 | -1.89 | 0.00 | 0.01 |
| ENSRNOG00000027894 | Iqgap3 | 0.13 | -2.92 | 0.00 | 0.00 |
| ENSRNOG00000025406 | Iqgap2 | 0.21 | -2.29 | 0.00 | 0.04 |
| ENSRNOG00000014320 | Inhba | 0.10 | -3.34 | 0.00 | 0.00 |
| ENSRNOG00000028650 | Inf2 | 0.27 | -1.90 | 0.00 | 0.01 |
| ENSRNOG00000016308 | Il10ra | 0.37 | -1.43 | 0.00 | 0.00 |
| ENSRNOG00000004273 | Ifitm1 | 0.06 | -4.03 | 0.00 | 0.05 |
| ENSRNOG00000020694 | Icam5 | 0.08 | -3.59 | 0.00 | 0.00 |
| ENSRNOG00000001270 | Hvcn1 | 0.31 | -1.69 | 0.00 | 0.00 |
| ENSRNOG00000049761 | Htr6 | 0.11 | -3.21 | 0.00 | 0.00 |
| ENSRNOG00000047014 | Homer1 | 0.17 | -2.54 | 0.00 | 0.02 |
| ENSRNOG00000012302 | Gucy1a1 | 0.19 | -2.37 | 0.00 | 0.01 |
| ENSRNOG00000016999 | Grp | 9.09 | 3.18 | 0.00 | 0.00 |
| ENSRNOG00000016429 | Grm5 | 0.19 | -2.40 | 0.00 | 0.02 |
| ENSRNOG00000013171 | Grm2 | 3.85 | 1.95 | 0.00 | 0.00 |
| ENSRNOG00000012562 | Grin3b | 0.31 | -1.69 | 0.00 | 0.02 |
| ENSRNOG00000001575 | Grik1 | 2.81 | 1.49 | 0.00 | 0.00 |
| ENSRNOG00000007346 | Grasp | 0.25 | -2.01 | 0.00 | 0.01 |
| ENSRNOG00000023657 | Gprin3 | 0.20 | -2.36 | 0.00 | 0.02 |
| ENSRNOG00000026953 | Gpr88 | 0.04 | -4.65 | 0.00 | 0.01 |
| ENSRNOG00000055673 | Gpr52 | 0.09 | -3.45 | 0.00 | 0.00 |
| ENSRNOG00000027658 | Gpr101 | 0.29 | -1.77 | 0.00 | 0.01 |
| ENSRNOG00000019857 | Gng7 | 0.07 | -3.87 | 0.00 | 0.02 |
| ENSRNOG00000011599 | Gldc | 0.18 | -2.49 | 0.00 | 0.01 |
| ENSRNOG00000018282 | Gda | 0.09 | -3.51 | 0.00 | 0.00 |
| ENSRNOG00000019495 | Gbx2 | 17.18 | 4.10 | 0.00 | 0.00 |
| ENSRNOG00000049361 | Gas7 | 0.24 | -2.05 | 0.00 | 0.04 |
| ENSRNOG00000061182 | Gabre | 3.44 | 1.78 | 0.00 | 0.05 |
| ENSRNOG00000002349 | Gabra2 | 0.30 | -1.72 | 0.00 | 0.04 |
| ENSRNOG00000008431 | Gabbr2 | 2.58 | 1.37 | 0.00 | 0.01 |
| ENSRNOG00000004898 | Fshb | 2040.53 | 10.99 | 0.00 | 0.01 |
| ENSRNOG00000007329 | Frmd6 | 0.33 | -1.61 | 0.00 | 0.01 |
| ENSRNOG00000021670 | Frem2 | 0.04 | -4.59 | 0.00 | 0.00 |
| ENSRNOG00000009184 | Foxp1 | 0.16 | -2.62 | 0.00 | 0.01 |
| ENSRNOG00000013397 | Foxo1 | 0.30 | -1.74 | 0.00 | 0.03 |
| ENSRNOG00000047891 | Foxg1 | 0.03 | -5.13 | 0.00 | 0.00 |
| ENSRNOG00000046667 | Fosb | 0.18 | -2.44 | 0.00 | 0.00 |
| ENSRNOG00000019902 | Folr1 | 0.03 | -5.21 | 0.00 | 0.01 |
| ENSRNOG00000011521 | Filip1 | 0.31 | -1.68 | 0.00 | 0.03 |
| ENSRNOG00000009206 | Fezf2 | 0.08 | -3.64 | 0.00 | 0.04 |
| ENSRNOG00000011774 | Fblim1 | 0.40 | -1.32 | 0.00 | 0.04 |
| ENSRNOG00000052758 | Fam49a | 0.26 | -1.95 | 0.00 | 0.01 |
| ENSRNOG00000033261 | Fam107a | 0.17 | -2.58 | 0.00 | 0.01 |
| ENSRNOG00000023389 | Ephx4 | 0.23 | -2.12 | 0.00 | 0.04 |
| ENSRNOG00000015719 | Egr4 | 0.03 | -5.15 | 0.00 | 0.00 |
| ENSRNOG00000000640 | Egr2 | 0.05 | -4.36 | 0.00 | 0.00 |
| ENSRNOG00000014648 | Efnb2 | 0.20 | -2.32 | 0.00 | 0.01 |
| ENSRNOG00000023688 | Drd1 | 0.04 | -4.82 | 0.00 | 0.01 |
| ENSRNOG00000025860 | Drc7 | 0.11 | -3.17 | 0.00 | 0.02 |
| ENSRNOG00000021573 | Dpy19l3 | 0.35 | -1.50 | 0.00 | 0.04 |
| ENSRNOG00000005451 | Dnah11 | 0.24 | -2.03 | 0.00 | 0.00 |
| ENSRNOG00000055934 | Dmkn | 0.08 | -3.62 | 0.00 | 0.01 |
| ENSRNOG00000010822 | Dlx6 | 0.07 | -3.90 | 0.00 | 0.01 |
| ENSRNOG00000012573 | Dlgap2 | 0.12 | -3.06 | 0.00 | 0.01 |
| ENSRNOG00000010065 | Dgkh | 0.14 | -2.81 | 0.00 | 0.04 |
| ENSRNOG00000023465 | Depp1 | 0.19 | -2.43 | 0.00 | 0.00 |
| ENSRNOG00000059605 | Ddn | 0.06 | -4.14 | 0.00 | 0.02 |
| ENSRNOG00000033026 | Dclk3 | 0.21 | -2.25 | 0.00 | 0.03 |
| ENSRNOG00000008834 | Dach1 | 0.13 | -2.91 | 0.00 | 0.01 |
| ENSRNOG00000004772 | Cytip | 0.05 | -4.19 | 0.00 | 0.00 |
| ENSRNOG00000005359 | Csrnp3 | 0.31 | -1.71 | 0.00 | 0.04 |
| ENSRNOG00000061215 | Crym | 0.05 | -4.36 | 0.00 | 0.00 |
| ENSRNOG00000023633 | Crabp1 | 0.17 | -2.55 | 0.00 | 0.00 |
| ENSRNOG00000015397 | Cpne7 | 3.88 | 1.95 | 0.00 | 0.00 |
| ENSRNOG00000000522 | Cpne5 | 0.22 | -2.17 | 0.00 | 0.05 |
| ENSRNOG00000001229 | Col18a1 | 0.46 | -1.11 | 0.00 | 0.02 |
| ENSRNOG00000005286 | Coch | 0.18 | -2.48 | 0.00 | 0.03 |
| ENSRNOG00000008223 | Cnr1 | 0.23 | -2.10 | 0.00 | 0.04 |
| ENSRNOG00000007014 | Cnksr2 | 0.18 | -2.51 | 0.00 | 0.03 |
| ENSRNOG00000023803 | Cmya5 | 0.14 | -2.82 | 0.00 | 0.00 |
| ENSRNOG00000011332 | Clspn | 0.06 | -4.12 | 0.00 | 0.00 |
| ENSRNOG00000003654 | Cldn9 | 0.34 | -1.55 | 0.00 | 0.02 |
| ENSRNOG00000018752 | Clcf1 | 0.05 | -4.19 | 0.00 | 0.00 |
| ENSRNOG00000016267 | Chst15 | 0.13 | -2.92 | 0.00 | 0.00 |
| ENSRNOG00000018385 | Chrm1 | 0.13 | -2.89 | 0.00 | 0.04 |
| ENSRNOG00000057347 | Cebpb | 0.37 | -1.43 | 0.00 | 0.04 |
| ENSRNOG00000004148 | Cdk17 | 0.29 | -1.79 | 0.00 | 0.04 |
| ENSRNOG00000007483 | Ccnf | 0.26 | -1.96 | 0.00 | 0.01 |
| ENSRNOG00000015036 | Ccn2 | 0.35 | -1.50 | 0.00 | 0.00 |
| ENSRNOG00000019321 | Cck | 5.07 | 2.34 | 0.00 | 0.01 |
| ENSRNOG00000010412 | Ccdc180 | 0.13 | -2.98 | 0.00 | 0.02 |
| ENSRNOG00000001701 | Cbr3 | 0.12 | -3.04 | 0.00 | 0.01 |
| ENSRNOG00000047367 | Card14 | 0.10 | -3.29 | 0.00 | 0.00 |
| ENSRNOG00000017882 | Camk1d | 0.27 | -1.87 | 0.00 | 0.02 |
| ENSRNOG00000003245 | Cacng1 | 0.00 | -12.25 | 0.00 | 0.00 |
| ENSRNOG00000017766 | Ca12 | 0.11 | -3.20 | 0.00 | 0.00 |
| ENSRNOG00000042163 | Btbd19 | 0.32 | -1.63 | 0.00 | 0.01 |
| ENSRNOG00000013717 | Bmp6 | 0.45 | -1.16 | 0.00 | 0.00 |
| ENSRNOG00000001304 | Bcr | 0.25 | -2.00 | 0.00 | 0.02 |
| ENSRNOG00000005776 | Bcl11b | 0.07 | -3.88 | 0.00 | 0.00 |
| ENSRNOG00000004049 | Baiap2 | 0.09 | -3.48 | 0.00 | 0.00 |
| ENSRNOG00000009267 | B3gnt2 | 0.41 | -1.29 | 0.00 | 0.05 |
| ENSRNOG00000004026 | Atp2b1 | 0.20 | -2.32 | 0.00 | 0.04 |
| ENSRNOG00000019985 | Asic4 | 0.31 | -1.71 | 0.00 | 0.02 |
| ENSRNOG00000051619 | Asb2 | 0.27 | -1.90 | 0.00 | 0.00 |
| ENSRNOG00000002256 | Art3 | 0.31 | -1.71 | 0.00 | 0.03 |
| ENSRNOG00000036880 | Arl5c | 0.11 | -3.16 | 0.00 | 0.00 |
| ENSRNOG00000020770 | Arl4d | 0.17 | -2.56 | 0.00 | 0.01 |
| ENSRNOG00000011105 | Arl15 | 0.18 | -2.45 | 0.00 | 0.01 |
| ENSRNOG00000006946 | Arhgap9 | 0.37 | -1.44 | 0.00 | 0.00 |
| ENSRNOG00000024677 | Arhgap33 | 0.22 | -2.15 | 0.00 | 0.03 |
| ENSRNOG00000043465 | Arc | 0.10 | -3.38 | 0.00 | 0.00 |
| ENSRNOG00000004731 | Ano3 | 0.07 | -3.81 | 0.00 | 0.03 |
| ENSRNOG00000042446 | Ankrd63 | 0.03 | -5.25 | 0.00 | 0.02 |
| ENSRNOG00000010888 | Ankrd33b | 0.08 | -3.57 | 0.00 | 0.02 |
| ENSRNOG00000025037 | Ankk1 | 0.10 | -3.39 | 0.00 | 0.01 |
| ENSRNOG00000030869 | Aldoart2 | 0.16 | -2.69 | 0.00 | 0.00 |
| ENSRNOG00000006410 | Akap5 | 0.06 | -3.96 | 0.00 | 0.00 |
| ENSRNOG00000025584 | Agap2 | 0.17 | -2.53 | 0.00 | 0.02 |
| ENSRNOG00000002232 | Aff1 | 0.49 | -1.03 | 0.00 | 0.02 |
| ENSRNOG00000009299 | Adra2c | 0.12 | -3.03 | 0.00 | 0.01 |
| ENSRNOG00000001302 | Adora2a | 0.04 | -4.50 | 0.00 | 0.01 |
| ENSRNOG00000002229 | Adcy5 | 0.15 | -2.69 | 0.00 | 0.01 |
| ENSRNOG00000059479 | Adcy1 | 0.27 | -1.89 | 0.00 | 0.04 |
| ENSRNOG00000027463 | Adamts3 | 0.13 | -2.95 | 0.00 | 0.00 |
| ENSRNOG00000017833 | Actn2 | 0.09 | -3.54 | 0.00 | 0.00 |
| ENSRNOG00000056756 | Actn1 | 0.25 | -2.02 | 0.00 | 0.02 |
| ENSRNOG00000017786 | Acta1 | 0.10 | -3.39 | 0.00 | 0.00 |
| ENSRNOG00000046261 | Acp5 | 0.15 | -2.76 | 0.00 | 0.00 |
| ENSRNOG00000007816 | AC131483.1 | 0.08 | -3.66 | 0.00 | 0.05 |
| ENSRNOG00000062261 | AC111804.2 | 0.10 | -3.30 | 0.00 | 0.01 |
| ENSRNOG00000056454 | AC096600.1 | 0.14 | -2.79 | 0.00 | 0.02 |
| ENSRNOG00000062252 | AABR07072853.5 | 2.94 | 1.56 | 0.00 | 0.03 |
| ENSRNOG00000051221 | AABR07070310.1 | 0.04 | -4.55 | 0.00 | 0.01 |
| ENSRNOG00000053753 | AABR07070307.1 | 0.33 | -1.60 | 0.00 | 0.04 |
| ENSRNOG00000042321 | AABR07052588.1 | 0.10 | -3.38 | 0.00 | 0.00 |
| ENSRNOG00000053592 | AABR07050646.1 | 0.08 | -3.60 | 0.00 | 0.01 |
| ENSRNOG00000052899 | AABR07049886.2 | 0.20 | -2.30 | 0.00 | 0.04 |
| ENSRNOG00000052122 | AABR07044366.1 | 0.33 | -1.59 | 0.00 | 0.00 |
| ENSRNOG00000014264 | AABR07027306.1 | 0.19 | -2.37 | 0.00 | 0.01 |
| ENSRNOG00000024294 | AABR07019083.1 | 0.37 | -1.42 | 0.00 | 0.00 |
| ENSRNOG00000060406 | AABR07017159.1 | 30634.69 | 14.90 | 0.00 | 0.00 |
| ENSRNOG00000004692 | A1bg | 0.00 | -10.32 | 0.00 | 0.01 |
