# Supplementary material for: Integrated network pharmacology and transcriptomics to explore the mechanism of compound Dihuang granule (CDG) protects dopaminergic neurons by regulating the Nrf2/HMOX1 pathway in the 6-OHDA/MPP+-induced model of Parkinson’s disease
Source: Chin Med. 2024 Dec 18;19:170. doi: 10.1186/s13020-024-01040-7 (PMC11654441; doi:10.1186/s13020-024-01040-7)
Supplement: Supplementary file 9 — Supplementray Material 9. [file 13020_2024_1040_MOESM9_ESM.docx]

| **List of differentially expressed genes analysed by Model VS CDG RNA-seq** | | | | | |
| --- | --- | --- | --- | --- | --- |
| **GeneID** | **Gene_Name** | **Fold Change** | **log2(fc)** | **pval** | **qval** |
| ENSRNOG00000049939 | AABR07066677.1 | 273598.2133 | 18.06169928 | 8.20952E-19 | 6.04275E-15 |
| ENSRNOG00000060406 | AABR07017159.1 | 30634.69 | 14.90287863 | 3.79704E-08 | 3.22486E-05 |
| ENSRNOG00000052514 | AABR07055191.1 | 6773.09 | 12.72559845 | 2.84796E-07 | 0.000157222 |
| ENSRNOG00000005166 | Nhlh1 | 6717.083333 | 12.71361921 | 8.86852E-08 | 6.31725E-05 |
| ENSRNOG00000059262 | AABR07024786.1 | 4865.793333 | 12.24845933 | 1.00634E-07 | 6.87588E-05 |
| ENSRNOG00000056729 | AABR07001780.1 | 4323.29 | 12.0779139 | 9.39775E-05 | 0.011402257 |
| ENSRNOG00000053400 | Cfi | 2416.526667 | 11.2387192 | 3.40352E-06 | 0.001342079 |
| ENSRNOG00000004898 | Fshb | 2040.533333 | 10.99473056 | 0.000188974 | 0.017243505 |
| ENSRNOG00000011092 | Iqca1l | 1551.620976 | 10.59956047 | 2.50847E-05 | 0.005430592 |
| ENSRNOG00000012057 | Olig3 | 1122.61 | 10.1326411 | 0.001172822 | 0.048045393 |
| ENSRNOG00000010348 | Cacna1f | 754.6133333 | 9.559593781 | 5.20937E-06 | 0.001949719 |
| ENSRNOG00000019495 | Gbx2 | 13.40008866 | 3.744170642 | 2.75469E-08 | 2.76496E-05 |
| ENSRNOG00000016999 | Grp | 7.690200881 | 2.943021284 | 0.000217943 | 0.018580108 |
| ENSRNOG00000003845 | Wnt3 | 5.317806226 | 2.410831208 | 6.43149E-05 | 0.009222087 |
| ENSRNOG00000014385 | Wnt2b | 5.097245613 | 2.349717872 | 0.000416747 | 0.026692731 |
| ENSRNOG00000061182 | Gabre | 4.784559425 | 2.258386083 | 1.88708E-05 | 0.004433038 |
| ENSRNOG00000006956 | AABR07049085.1 | 4.576403139 | 2.194214146 | 3.39716E-11 | 7.50161E-08 |
| ENSRNOG00000019321 | Cck | 4.472149826 | 2.160968522 | 9.84386E-05 | 0.0118137 |
| ENSRNOG00000033970 | Moap1 | 4.346592515 | 2.119884852 | 1.32021E-07 | 8.09804E-05 |
| ENSRNOG00000006397 | Chrm5 | 4.342432704 | 2.118503491 | 0.001052759 | 0.04505739 |
| ENSRNOG00000031136 | Ntng1 | 4.142856504 | 2.050625851 | 0.000547465 | 0.031077436 |
| ENSRNOG00000015397 | Cpne7 | 3.639350202 | 1.863680883 | 0.000236578 | 0.019492986 |
| ENSRNOG00000016804 | Il25 | 3.408322117 | 1.769061689 | 0.000774738 | 0.0376823 |
| ENSRNOG00000013171 | Grm2 | 3.243913186 | 1.69773521 | 7.16571E-06 | 0.002421225 |
| ENSRNOG00000014505 | Pmfbp1 | 3.210724144 | 1.682898718 | 0.000906673 | 0.04135735 |
| ENSRNOG00000062252 | AABR07072853.5 | 3.029718459 | 1.599183735 | 0.00106038 | 0.045236068 |
| ENSRNOG00000061526 | Rsph6a | 2.779267076 | 1.474704478 | 0.000279127 | 0.021401709 |
| ENSRNOG00000026091 | Slc10a4 | 2.766976823 | 1.46831056 | 0.000955048 | 0.042777637 |
| ENSRNOG00000006263 | Sh2d1a | 2.692906073 | 1.429163911 | 0.000540743 | 0.031077436 |
| ENSRNOG00000001575 | Grik1 | 2.681238716 | 1.422899671 | 0.00010384 | 0.012133239 |
| ENSRNOG00000009450 | Hcn4 | 2.654685275 | 1.408540833 | 0.000534903 | 0.030920769 |
| ENSRNOG00000008011 | Agbl4 | 2.638237191 | 1.399574276 | 3.80654E-05 | 0.007065383 |
| ENSRNOG00000015055 | Scg2 | 2.616494832 | 1.387635409 | 0.000230924 | 0.019098348 |
| ENSRNOG00000033119 | Plcb4 | 2.559525794 | 1.355876546 | 0.000239093 | 0.019520147 |
| ENSRNOG00000026974 | Dbndd1 | 2.49406108 | 1.318496797 | 0.000834266 | 0.039532759 |
| ENSRNOG00000018257 | Hpx | 2.44153332 | 1.287787467 | 0.000154253 | 0.015138739 |
| ENSRNOG00000007092 | Fgb | 2.440358901 | 1.287093339 | 1.71129E-05 | 0.00406329 |
| ENSRNOG00000003832 | Vash2 | 2.36544447 | 1.242111294 | 0.000658982 | 0.034128631 |
| ENSRNOG00000016388 | Sphkap | 2.337267046 | 1.22482258 | 5.6297E-11 | 1.03596E-07 |
| ENSRNOG00000003622 | Cybb | 2.328411871 | 1.219346278 | 5.20103E-05 | 0.008151371 |
| ENSRNOG00000032350 | Kcnip4 | 2.281918334 | 1.190247161 | 0.000168922 | 0.015872908 |
| ENSRNOG00000059539 | LOC103693564 | 2.27442459 | 1.185501602 | 0.000710655 | 0.035665207 |
| ENSRNOG00000016737 | Tcerg1l | 2.257871386 | 1.174963309 | 0.000819443 | 0.039315607 |
| ENSRNOG00000008431 | Gabbr2 | 2.25679837 | 1.174277529 | 0.000862097 | 0.04018666 |
| ENSRNOG00000025676 | Gask1a | 2.237272393 | 1.161740918 | 0.000932366 | 0.042073698 |
| ENSRNOG00000007151 | Cdk14 | 2.212285966 | 1.145537884 | 0.001017573 | 0.044058931 |
| ENSRNOG00000060687 | Slc24a3 | 2.206934339 | 1.142043707 | 5.24033E-13 | 1.92862E-09 |
| ENSRNOG00000059720 | Syp | 2.080703427 | 1.057071345 | 0.000144729 | 0.014526874 |
| ENSRNOG00000010777 | Tox | 2.029264251 | 1.020956745 | 0.000321153 | 0.023132404 |
| ENSRNOG00000017209 | Tubb3 | 2.016186167 | 1.011628858 | 0.000751998 | 0.036983565 |
| ENSRNOG00000007949 | Rgn | 2.007376458 | 1.005311201 | 0.000637927 | 0.033752925 |
| ENSRNOG00000009360 | Sh3bp1 | 0.497629772 | -1.006855293 | 0.000963667 | 0.042902619 |
| ENSRNOG00000008168 | Wnt5b | 0.496207164 | -1.010985532 | 0.000917177 | 0.041673046 |
| ENSRNOG00000057347 | Cebpb | 0.494194128 | -1.016850227 | 5.00382E-05 | 0.008137645 |
| ENSRNOG00000013717 | Bmp6 | 0.48804459 | -1.034915131 | 7.95686E-05 | 0.01033549 |
| ENSRNOG00000007839 | Slc16a7 | 0.486199483 | -1.040379736 | 0.000361577 | 0.024719305 |
| ENSRNOG00000011913 | Cp | 0.482976113 | -1.049976258 | 0.000111432 | 0.012379624 |
| ENSRNOG00000017905 | Map1lc3b | 0.481584747 | -1.054138397 | 0.000827577 | 0.039415648 |
| ENSRNOG00000062127 | AC134224.1 | 0.480804346 | -1.056478158 | 1.87354E-10 | 3.18242E-07 |
| ENSRNOG00000031495 | Tmem170b | 0.480502783 | -1.057383307 | 0.00020692 | 0.018187846 |
| ENSRNOG00000002232 | Aff1 | 0.479006135 | -1.061883961 | 7.90864E-06 | 0.002568213 |
| ENSRNOG00000049255 | LOC100911837 | 0.477581279 | -1.066181811 | 0.000962009 | 0.042902619 |
| ENSRNOG00000009037 | Sulf1 | 0.476733082 | -1.068746351 | 2.98104E-08 | 2.84395E-05 |
| ENSRNOG00000016879 | Ldlrad4 | 0.47657269 | -1.069231814 | 6.87747E-05 | 0.009551471 |
| ENSRNOG00000003703 | Mcm6 | 0.473026066 | -1.080008409 | 0.000273182 | 0.021284137 |
| ENSRNOG00000008652 | Phip | 0.472084237 | -1.082883782 | 0.000563303 | 0.031280926 |
| ENSRNOG00000046600 | AABR07015066.1 | 0.470357212 | -1.08817127 | 4.93797E-07 | 0.000247819 |
| ENSRNOG00000015863 | Npsr1 | 5.054890599 | 2.337679869 | 9.83993E-06 | 0.002859017 |
| ENSRNOG00000009339 | Cenpe | 0.470263651 | -1.088458273 | 0.000659946 | 0.034128631 |
| ENSRNOG00000056747 | AABR07015055.1 | 0.467982254 | -1.09547427 | 1.53465E-06 | 0.000691597 |
| ENSRNOG00000020705 | Rnls | 0.464945969 | -1.104865025 | 0.000494446 | 0.029370173 |
| ENSRNOG00000015036 | Ccn2 | 0.458122464 | -1.126194788 | 1.14336E-06 | 0.000537187 |
| ENSRNOG00000055956 | AABR07015078.1 | 0.453628642 | -1.140416359 | 6.66596E-08 | 4.90659E-05 |
| ENSRNOG00000010210 | Slc7a11 | 0.4519109 | -1.14588974 | 6.00089E-08 | 4.56937E-05 |
| ENSRNOG00000022619 | Fth1 | 0.446602869 | -1.162935575 | 0.000517358 | 0.030303181 |
| ENSRNOG00000003872 | NEWGENE_620180 | 0.445706327 | -1.165834654 | 0.00032122 | 0.023132404 |
| ENSRNOG00000023299 | Hfm1 | 0.442843601 | -1.175130821 | 0.000742873 | 0.03661632 |
| ENSRNOG00000000525 | Pi16 | 0.439773624 | -1.185167015 | 1.1927E-07 | 7.74626E-05 |
| ENSRNOG00000053787 | Mdfic | 0.439682139 | -1.185467168 | 1.70105E-07 | 9.88491E-05 |
| ENSRNOG00000050156 | AABR07063425.2 | 0.43857475 | -1.189105342 | 0.000245521 | 0.019932346 |
| ENSRNOG00000004873 | Prkch | 0.437688231 | -1.192024504 | 0.000986836 | 0.043399701 |
| ENSRNOG00000013322 | Pola1 | 0.436112537 | -1.19722763 | 0.000409027 | 0.026621166 |
| ENSRNOG00000001229 | Col18a1 | 0.433810093 | -1.204864476 | 7.63934E-06 | 0.00251779 |
| ENSRNOG00000051507 | Azin2 | 0.43080996 | -1.214876491 | 0.001011732 | 0.043978492 |
| ENSRNOG00000060896 | AABR07063424.1 | 0.429120951 | -1.220543757 | 2.16918E-09 | 2.81764E-06 |
| ENSRNOG00000017277 | Igsf6 | 0.413520509 | -1.273969211 | 0.000772871 | 0.037674475 |
| ENSRNOG00000054945 | AABR07015081.2 | 0.411763716 | -1.280111389 | 7.45547E-12 | 2.0579E-08 |
| ENSRNOG00000024294 | AABR07019083.1 | 0.411520446 | -1.280963985 | 6.94768E-05 | 0.009588663 |
| ENSRNOG00000026917 | AABR07021573.2 | 0.409271006 | -1.28887163 | 0.000153022 | 0.015085006 |
| ENSRNOG00000006946 | Arhgap9 | 0.406767912 | -1.297722219 | 3.56582E-05 | 0.006782987 |
| ENSRNOG00000001548 | Nrf2 | 0.405585453 | -1.301922186 | 0.001141211 | 0.047191428 |
| ENSRNOG00000055154 | AABR07063462.1 | 0.403702423 | -1.308635849 | 1.12772E-09 | 1.55639E-06 |
| ENSRNOG00000012480 | Pxylp1 | 0.402044451 | -1.314573078 | 0.000709239 | 0.035665207 |
| ENSRNOG00000024705 | Rarres2 | 0.401928521 | -1.314989138 | 0.00044964 | 0.027968863 |
| ENSRNOG00000046707 | AABR07063425.1 | 0.401194375 | -1.317626715 | 6.46865E-09 | 7.51793E-06 |
| ENSRNOG00000007331 | Rragd | 0.399702522 | -1.323001421 | 0.001084586 | 0.045793168 |
| ENSRNOG00000009482 | Emx2 | 0.397970765 | -1.329265641 | 0.000542407 | 0.031077436 |
| ENSRNOG00000015701 | Rreb1 | 0.396639227 | -1.334100732 | 7.54629E-05 | 0.009918882 |
| ENSRNOG00000025604 | Atad2 | 0.388832715 | -1.362778489 | 0.000856532 | 0.040156968 |
| ENSRNOG00000009243 | Oaf | 0.386414255 | -1.371779778 | 0.00015676 | 0.015316719 |
| ENSRNOG00000002496 | Stxbp5l | 0.381809102 | -1.389076597 | 0.001145509 | 0.047192389 |
| ENSRNOG00000019768 | Ncoa4 | 0.37803466 | -1.40340958 | 0.000403067 | 0.026489631 |
| ENSRNOG00000042163 | Btbd19 | 0.374137022 | -1.418361363 | 0.000110501 | 0.012379624 |
| ENSRNOG00000006972 | Zfp189 | 0.372419086 | -1.425001082 | 0.000373363 | 0.025247994 |
| ENSRNOG00000016177 | Scara3 | 0.371681271 | -1.4278621 | 7.36652E-07 | 0.000361483 |
| ENSRNOG00000016258 | Zfp516 | 0.371246294 | -1.429551472 | 0.000398867 | 0.026489631 |
| ENSRNOG00000060518 | AABR07015057.1 | 0.3710252 | -1.430410916 | 2.89876E-13 | 1.28021E-09 |
| ENSRNOG00000011775 | Mfap3l | 0.367528317 | -1.444072687 | 0.000935381 | 0.042073698 |
| ENSRNOG00000048268 | AABR07015067.1 | 0.367117461 | -1.445686361 | 4.11156E-07 | 0.000211143 |
| ENSRNOG00000062155 | AC134224.2 | 0.365898709 | -1.450483771 | 5.283E-11 | 1.03596E-07 |
| ENSRNOG00000023465 | Depp1 | 0.362897396 | -1.462366388 | 8.39212E-05 | 0.010742835 |
| ENSRNOG00000013604 | Gpx4 | 0.359994978 | -1.473951313 | 0.000583928 | 0.031859249 |
| ENSRNOG00000008680 | Loxl1 | 0.358202658 | -1.481152053 | 1.13156E-06 | 0.000537187 |
| ENSRNOG00000007329 | Frmd6 | 0.357667652 | -1.48330845 | 0.000220451 | 0.018580108 |
| ENSRNOG00000003537 | Spta1 | 0.357095489 | -1.485618186 | 2.70593E-06 | 0.001116549 |
| ENSRNOG00000047351 | AABR07015081.1 | 0.356829007 | -1.486695195 | 5.20681E-14 | 2.87442E-10 |
| ENSRNOG00000021573 | Dpy19l3 | 0.356356625 | -1.48860635 | 0.000324746 | 0.023132404 |
| ENSRNOG00000054657 | AABR07015067.2 | 0.355262007 | -1.493044684 | 3.21814E-11 | 7.50161E-08 |
| ENSRNOG00000050545 | AABR07015056.1 | 0.353487818 | -1.500267596 | 2.41662E-10 | 3.8117E-07 |
| ENSRNOG00000002256 | Art3 | 0.349370821 | -1.517168973 | 0.000563799 | 0.031280926 |
| ENSRNOG00000016846 | Pik3cd | 0.349218424 | -1.517798419 | 0.000525277 | 0.030596646 |
| ENSRNOG00000020579 | Col7a1 | 0.346723116 | -1.528144071 | 0.000649247 | 0.034053873 |
| ENSRNOG00000059504 | AABR07015078.2 | 0.346219182 | -1.530242438 | 5.91832E-20 | 6.53442E-16 |
| ENSRNOG00000020441 | Wnk4 | 0.343230286 | -1.542751238 | 0.000409456 | 0.026621166 |
| ENSRNOG00000045913 | Prdm16 | 0.342659849 | -1.545150939 | 0.000538592 | 0.031052724 |
| ENSRNOG00000027938 | Jcad | 0.339707135 | -1.557636573 | 0.000666072 | 0.034364983 |
| ENSRNOG00000027658 | Gpr101 | 0.339301955 | -1.559358351 | 0.000487784 | 0.029370173 |
| ENSRNOG00000047746 | AABR07000398.1 | 0.33844017 | -1.563027281 | 2.36969E-06 | 0.001026029 |
| ENSRNOG00000046980 | LOC108348151 | 0.337265076 | -1.568045159 | 0.00121497 | 0.049227448 |
| ENSRNOG00000016308 | Il10ra | 0.335519871 | -1.575529881 | 2.12625E-08 | 2.23581E-05 |
| ENSRNOG00000014424 | RGD1563354 | 0.332423772 | -1.588904541 | 0.000402059 | 0.026489631 |
| ENSRNOG00000012508 | Slc39a8 | 0.330843064 | -1.595781059 | 1.4175E-06 | 0.000652109 |
| ENSRNOG00000004362 | Rps6ka5 | 0.328504261 | -1.606016012 | 0.000589119 | 0.03196298 |
| ENSRNOG00000002525 | Ptgs2 | 0.328422405 | -1.606375544 | 8.63373E-05 | 0.010894286 |
| ENSRNOG00000053753 | AABR07070307.1 | 0.328372543 | -1.606594593 | 1.23258E-05 | 0.003390923 |
| ENSRNOG00000002349 | Gabra2 | 0.327909338 | -1.60863111 | 0.000388566 | 0.026080015 |
| ENSRNOG00000029260 | Pitpnm2 | 0.324846547 | -1.622169726 | 0.001240043 | 0.049877285 |
| ENSRNOG00000009832 | Slc39a14 | 0.32247173 | -1.632755405 | 0.000974462 | 0.043036124 |
| ENSRNOG00000003120 | Prelp | 0.318344184 | -1.651340688 | 3.73526E-08 | 3.22486E-05 |
| ENSRNOG00000016636 | Lpin3 | 0.315934158 | -1.66230417 | 0.001094233 | 0.046112301 |
| ENSRNOG00000018305 | St8sia3 | 0.314570672 | -1.668543924 | 0.001021984 | 0.044163311 |
| ENSRNOG00000003654 | Cldn9 | 0.312673687 | -1.677270282 | 0.000277748 | 0.02137012 |
| ENSRNOG00000005451 | Dnah11 | 0.312556484 | -1.677811165 | 0.000886759 | 0.040655699 |
| ENSRNOG00000014486 | Rfx3 | 0.310814029 | -1.685876472 | 0.000418677 | 0.026692731 |
| ENSRNOG00000016467 | Kctd1 | 0.30804378 | -1.698792692 | 0.000971862 | 0.043036124 |
| ENSRNOG00000005479 | Slc1a2 | 0.307825855 | -1.699813681 | 6.39366E-05 | 0.009222087 |
| ENSRNOG00000005807 | Ptpn7 | 0.307343369 | -1.702076737 | 0.000490037 | 0.029370173 |
| ENSRNOG00000000800 | Man1a1 | 0.303923298 | -1.718220823 | 0.000310269 | 0.023101737 |
| ENSRNOG00000003809 | Sat1 | 0.303001438 | -1.722603455 | 1.92994E-05 | 0.004439257 |
| ENSRNOG00000011774 | Fblim1 | 0.301970958 | -1.727518291 | 9.12084E-06 | 0.002685418 |
| ENSRNOG00000030625 | Tf | 0.301293145 | -1.730760244 | 0.000300632 | 0.022713667 |
| ENSRNOG00000029465 | Slc26a10 | 0.300425289 | -1.734921833 | 0.000268264 | 0.021156434 |
| ENSRNOG00000007646 | Sipa1l1 | 0.298701403 | -1.743224083 | 0.000463841 | 0.028610423 |
| ENSRNOG00000007364 | Rab15 | 0.298115933 | -1.746054611 | 0.001121763 | 0.0467373 |
| ENSRNOG00000016983 | Myh7 | 0.298047129 | -1.74638762 | 9.03976E-05 | 0.011159437 |
| ENSRNOG00000007483 | Ccnf | 0.297713044 | -1.748005662 | 0.000189962 | 0.017262329 |
| ENSRNOG00000004148 | Cdk17 | 0.29638572 | -1.754452153 | 0.001001139 | 0.043690009 |
| ENSRNOG00000029441 | Klhl2 | 0.293966837 | -1.766274684 | 0.000726227 | 0.036026243 |
| ENSRNOG00000010111 | Exoc3l4 | 0.293702833 | -1.767570913 | 0.00059222 | 0.031974093 |
| ENSRNOG00000012181 | Lpl | 0.29180334 | -1.776931698 | 0.00118554 | 0.048334236 |
| ENSRNOG00000011521 | Filip1 | 0.291716176 | -1.777362708 | 0.000324365 | 0.023132404 |
| ENSRNOG00000013397 | Foxo1 | 0.290262809 | -1.784568362 | 0.000862625 | 0.04018666 |
| ENSRNOG00000008415 | Nab2 | 0.286745097 | -1.802159274 | 0.000770029 | 0.037619 |
| ENSRNOG00000023661 | Celf2 | 0.282507926 | -1.823636749 | 0.001067415 | 0.045415533 |
| ENSRNOG00000057729 | Strip2 | 0.279146511 | -1.840905569 | 0.001145056 | 0.047192389 |
| ENSRNOG00000017882 | Camk1d | 0.278852347 | -1.842426683 | 0.000573566 | 0.031506168 |
| ENSRNOG00000010280 | Pde8b | 0.278784694 | -1.84277674 | 0.000703063 | 0.035526406 |
| ENSRNOG00000055564 | RGD1564664 | 0.277966886 | -1.847015068 | 0.000122925 | 0.013241163 |
| ENSRNOG00000012038 | Htr1d | 0.277215106 | -1.850922219 | 0.000645094 | 0.033997528 |
| ENSRNOG00000005359 | Csrnp3 | 0.276072658 | -1.856880082 | 0.00050795 | 0.029831259 |
| ENSRNOG00000015401 | Mapk4 | 0.275338785 | -1.860720251 | 0.000228326 | 0.018954478 |
| ENSRNOG00000059586 | AABR07015080.2 | 0.275015142 | -1.862417041 | 6.95828E-13 | 2.19504E-09 |
| ENSRNOG00000051384 | AABR07002337.1 | 0.272737038 | -1.874417463 | 0.000261163 | 0.020744605 |
| ENSRNOG00000060414 | AABR07052730.2 | 0.269581061 | -1.891208947 | 0.000478614 | 0.029195458 |
| ENSRNOG00000001304 | Bcr | 0.268315555 | -1.897997403 | 0.00044506 | 0.027919922 |
| ENSRNOG00000019985 | Asic4 | 0.267673635 | -1.901453048 | 9.51763E-05 | 0.011484609 |
| ENSRNOG00000022704 | Esyt3 | 0.266789574 | -1.906225809 | 0.001151477 | 0.047349936 |
| ENSRNOG00000051619 | Asb2 | 0.2648502 | -1.916751498 | 0.000185419 | 0.017060055 |
| ENSRNOG00000010555 | LOC108348161 | 0.264419958 | -1.919097024 | 0.001061149 | 0.045236068 |
| ENSRNOG00000008766 | Grin2b | 0.261459577 | -1.935340178 | 0.000690761 | 0.035146055 |
| ENSRNOG00000007324 | Plxna2 | 0.260765577 | -1.93917466 | 0.000137495 | 0.014198673 |
| ENSRNOG00000025406 | Iqgap2 | 0.260270208 | -1.94191791 | 0.000719626 | 0.035790051 |
| ENSRNOG00000003479 | Rnf150 | 0.259585181 | -1.945720068 | 0.000320307 | 0.023132404 |
| ENSRNOG00000046667 | Fosb | 0.259242643 | -1.947625049 | 3.38952E-05 | 0.006565559 |
| ENSRNOG00000052758 | Fam49a | 0.258384509 | -1.952408517 | 0.00035715 | 0.024645589 |
| ENSRNOG00000019435 | Psd | 0.256075463 | -1.965359076 | 0.000496109 | 0.029370173 |
| ENSRNOG00000028650 | Inf2 | 0.253491369 | -1.979991469 | 0.000263612 | 0.020864073 |
| ENSRNOG00000003841 | Kcnh1 | 0.253229871 | -1.981480499 | 0.000501938 | 0.02963579 |
| ENSRNOG00000004810 | Plcb1 | 0.252599603 | -1.985075722 | 0.000383177 | 0.025796661 |
| ENSRNOG00000014723 | Cbfa2t3 | 0.252124732 | -1.987790452 | 0.000275666 | 0.021284137 |
| ENSRNOG00000019163 | Syt6 | 0.25194947 | -1.988793676 | 0.000424737 | 0.026937464 |
| ENSRNOG00000009514 | Mme | 0.24704669 | -2.017144369 | 0.00039294 | 0.026293637 |
| ENSRNOG00000007879 | Stk26 | 0.24604943 | -2.022979923 | 7.29994E-05 | 0.009752468 |
| ENSRNOG00000061774 | AABR07002910.1 | 0.245343429 | -2.027125464 | 0.001186358 | 0.048334236 |
| ENSRNOG00000006723 | Itga11 | 0.244825126 | -2.030176469 | 3.37441E-05 | 0.006565559 |
| ENSRNOG00000020332 | Tnnt3 | 0.244762151 | -2.030547613 | 2.4154E-07 | 0.000136761 |
| ENSRNOG00000058842 | Sptbn2 | 0.243205467 | -2.039752436 | 0.000601661 | 0.032247297 |
| ENSRNOG00000017786 | Acta1 | 0.241331532 | -2.050911666 | 4.82966E-08 | 3.94995E-05 |
| ENSRNOG00000033837 | Cdh9 | 0.239886584 | -2.05957562 | 0.001127773 | 0.04689922 |
| ENSRNOG00000007346 | Grasp | 0.238473157 | -2.068101214 | 8.50701E-05 | 0.010796085 |
| ENSRNOG00000056756 | Actn1 | 0.238252767 | -2.069435126 | 0.000462142 | 0.028585468 |
| ENSRNOG00000033694 | Klf16 | 0.237416154 | -2.074509994 | 0.000400531 | 0.026489631 |
| ENSRNOG00000049361 | Gas7 | 0.23350806 | -2.098455744 | 0.000817269 | 0.039315607 |
| ENSRNOG00000059479 | Adcy1 | 0.233425731 | -2.098964493 | 0.000184425 | 0.017039594 |
| ENSRNOG00000016877 | Shisa7 | 0.233067179 | -2.101182237 | 0.000360653 | 0.024719305 |
| ENSRNOG00000052899 | AABR07049886.2 | 0.231279636 | -2.112289853 | 0.000908356 | 0.04135735 |
| ENSRNOG00000013981 | Ptpn5 | 0.229198006 | -2.125333602 | 0.000493425 | 0.029370173 |
| ENSRNOG00000031671 | Rasgef1a | 0.228505329 | -2.129700282 | 0.000218389 | 0.018580108 |
| ENSRNOG00000048847 | Wdr17 | 0.225199985 | -2.150721362 | 0.000552254 | 0.031170047 |
| ENSRNOG00000008223 | Cnr1 | 0.224821991 | -2.153144938 | 0.000302764 | 0.022740217 |
| ENSRNOG00000000522 | Cpne5 | 0.223332755 | -2.162733234 | 0.000766635 | 0.0375362 |
| ENSRNOG00000021091 | Trank1 | 0.222488761 | -2.168195633 | 0.000566856 | 0.031371727 |
| ENSRNOG00000021098 | Rasgrp2 | 0.221446522 | -2.174969757 | 0.00022287 | 0.018641721 |
| ENSRNOG00000009882 | Ppp3ca | 0.219376284 | -2.188520527 | 0.000425739 | 0.026937464 |
| ENSRNOG00000049758 | Tbc1d16 | 0.218499196 | -2.194300123 | 0.000321968 | 0.023132404 |
| ENSRNOG00000019560 | Pde2a | 0.217627434 | -2.200067659 | 0.00057341 | 0.031506168 |
| ENSRNOG00000024677 | Arhgap33 | 0.214812949 | -2.218847133 | 0.000353719 | 0.024485343 |
| ENSRNOG00000034102 | Plk5 | 0.210267166 | -2.249704511 | 0.000433506 | 0.027272587 |
| ENSRNOG00000015691 | Inka2 | 0.209317015 | -2.256238507 | 0.000252472 | 0.020222992 |
| ENSRNOG00000021231 | Lzts3 | 0.2090668 | -2.257964114 | 0.000402622 | 0.026489631 |
| ENSRNOG00000014117 | Hmox1 | 0.209063989 | -2.257983512 | 0.000412092 | 0.026685705 |
| ENSRNOG00000023633 | Crabp1 | 0.208962105 | -2.25868676 | 2.49806E-05 | 0.005430592 |
| ENSRNOG00000047686 | Pou3f1 | 0.207772595 | -2.266922716 | 0.000922918 | 0.041847786 |
| ENSRNOG00000049695 | Myh4 | 0.205583174 | -2.2822059 | 5.31152E-08 | 4.18889E-05 |
| ENSRNOG00000019627 | Mybpc2 | 0.204075444 | -2.2928255 | 0.000795609 | 0.038612378 |
| ENSRNOG00000014648 | Efnb2 | 0.203746092 | -2.295155708 | 0.000219074 | 0.018580108 |
| ENSRNOG00000007575 | Plppr1 | 0.20150264 | -2.311129355 | 0.000482853 | 0.029292212 |
| ENSRNOG00000030869 | Aldoart2 | 0.200813065 | -2.316074962 | 0.000272278 | 0.021284137 |
| ENSRNOG00000007377 | Slit3 | 0.200305977 | -2.319722624 | 4.24844E-05 | 0.007505117 |
| ENSRNOG00000004026 | Atp2b1 | 0.199124233 | -2.328259287 | 0.000882197 | 0.040655699 |
| ENSRNOG00000058938 | Camkv | 0.198015864 | -2.336312076 | 0.000609751 | 0.032522986 |
| ENSRNOG00000019181 | Synpo | 0.197911056 | -2.337075889 | 0.000158842 | 0.015384005 |
| ENSRNOG00000002653 | Kcnk2 | 0.192994733 | -2.373366619 | 0.000124815 | 0.013379478 |
| ENSRNOG00000007014 | Cnksr2 | 0.192416542 | -2.377695265 | 0.000335962 | 0.023626481 |
| ENSRNOG00000014264 | AABR07027306.1 | 0.191300088 | -2.386090554 | 0.000252765 | 0.020222992 |
| ENSRNOG00000033026 | Dclk3 | 0.19106263 | -2.387882467 | 0.000561328 | 0.031280926 |
| ENSRNOG00000016167 | Spata2L | 0.190516934 | -2.392008859 | 0.000937428 | 0.042073738 |
| ENSRNOG00000005934 | Mlip | 0.189978991 | -2.396088206 | 0.000141377 | 0.014270701 |
| ENSRNOG00000006729 | Slc24a4 | 0.18885112 | -2.404678753 | 0.000220366 | 0.018580108 |
| ENSRNOG00000023389 | Ephx4 | 0.188722659 | -2.405660445 | 6.61397E-05 | 0.009422561 |
| ENSRNOG00000011105 | Arl15 | 0.187876239 | -2.412145477 | 0.000527911 | 0.030596646 |
| ENSRNOG00000019902 | Folr1 | 0.187491358 | -2.415103996 | 0.000692592 | 0.035158181 |
| ENSRNOG00000011951 | Plk2 | 0.186362577 | -2.423815913 | 0.00021092 | 0.018409233 |
| ENSRNOG00000056697 | Kcnab1 | 0.180664829 | -2.46861242 | 0.000322334 | 0.023132404 |
| ENSRNOG00000008312 | Stra6 | 0.178550593 | -2.485595169 | 3.08716E-05 | 0.006197327 |
| ENSRNOG00000005108 | Wfs1 | 0.175569538 | -2.50988554 | 0.000140315 | 0.014270701 |
| ENSRNOG00000016429 | Grm5 | 0.174937794 | -2.51508609 | 7.33135E-05 | 0.009752468 |
| ENSRNOG00000011599 | Gldc | 0.172274541 | -2.537218583 | 4.35831E-05 | 0.007638111 |
| ENSRNOG00000024479 | Klhl34 | 0.171299681 | -2.545405633 | 0.000121063 | 0.013141347 |
| ENSRNOG00000036703 | Itgax | 0.17086198 | -2.549096687 | 0.000717521 | 0.035790051 |
| ENSRNOG00000056817 | Muc6 | 0.170653376 | -2.550859136 | 0.000476896 | 0.02917124 |
| ENSRNOG00000012302 | Gucy1a1 | 0.170139238 | -2.555212201 | 7.20704E-05 | 0.009752468 |
| ENSRNOG00000007104 | Itpr1 | 0.169260158 | -2.562685673 | 0.000110817 | 0.012379624 |
| ENSRNOG00000008203 | Synpr | 0.168740535 | -2.567121515 | 9.28874E-05 | 0.011332266 |
| ENSRNOG00000011826 | Lzts1 | 0.168654758 | -2.567855079 | 0.000206426 | 0.018187846 |
| ENSRNOG00000047014 | Homer1 | 0.167856927 | -2.574696018 | 4.82625E-05 | 0.008013031 |
| ENSRNOG00000008620 | Smad3 | 0.16704171 | -2.581719705 | 8.74336E-06 | 0.002685418 |
| ENSRNOG00000001128 | Tesc | 0.166489606 | -2.586495987 | 0.000291346 | 0.022184468 |
| ENSRNOG00000046261 | Acp5 | 0.165047868 | -2.599043588 | 2.92114E-07 | 0.000157328 |
| ENSRNOG00000023657 | Gprin3 | 0.162366846 | -2.622671017 | 2.16412E-05 | 0.004926609 |
| ENSRNOG00000010165 | Tnfaip2 | 0.162211224 | -2.624054448 | 2.29744E-06 | 0.001014643 |
| ENSRNOG00000042326 | Smpdl3b | 0.158822481 | -2.654512962 | 6.01007E-05 | 0.008789024 |
| ENSRNOG00000054314 | Kcng1 | 0.158030244 | -2.661727409 | 0.000248157 | 0.020072523 |
| ENSRNOG00000023803 | Cmya5 | 0.157393617 | -2.667551062 | 0.00085122 | 0.040020393 |
| ENSRNOG00000005286 | Coch | 0.156840221 | -2.672632514 | 1.05472E-05 | 0.003024719 |
| ENSRNOG00000020770 | Arl4d | 0.154000489 | -2.698993161 | 0.000121404 | 0.013141347 |
| ENSRNOG00000005457 | Lamp5 | 0.15344167 | -2.704237763 | 5.4381E-05 | 0.00825056 |
| ENSRNOG00000016728 | Tiam2 | 0.153370772 | -2.704904521 | 4.70011E-05 | 0.008013031 |
| ENSRNOG00000000257 | Smpd3 | 0.151623768 | -2.721432169 | 5.64479E-05 | 0.008422179 |
| ENSRNOG00000004048 | Lrrk2 | 0.151463982 | -2.72295333 | 5.14569E-05 | 0.008151371 |
| ENSRNOG00000025584 | Agap2 | 0.150756213 | -2.729710639 | 8.7034E-05 | 0.010919802 |
| ENSRNOG00000025110 | Vwa3a | 0.150082703 | -2.73617038 | 4.76915E-05 | 0.008013031 |
| ENSRNOG00000049495 | Krt71 | 0.149881412 | -2.738106619 | 0.000160407 | 0.015400498 |
| ENSRNOG00000013436 | Pde7b | 0.149791191 | -2.738975313 | 0.00032437 | 0.023132404 |
| ENSRNOG00000025860 | Drc7 | 0.149299401 | -2.743719717 | 0.001231625 | 0.049660178 |
| ENSRNOG00000015321 | Moxd1 | 0.149233671 | -2.744355013 | 0.000990556 | 0.043399701 |
| ENSRNOG00000009184 | Foxp1 | 0.148863811 | -2.74793502 | 0.000137312 | 0.014198673 |
| ENSRNOG00000022071 | Itga2b | 0.147163416 | -2.76450903 | 7.44994E-05 | 0.009850879 |
| ENSRNOG00000019719 | Kcna5 | 0.145954657 | -2.776407853 | 0.000191066 | 0.017291449 |
| ENSRNOG00000013781 | Kcnq5 | 0.145259218 | -2.783298375 | 7.24679E-05 | 0.009752468 |
| ENSRNOG00000002229 | Adcy5 | 0.144133756 | -2.794519845 | 4.07555E-05 | 0.007437711 |
| ENSRNOG00000033261 | Fam107a | 0.142918566 | -2.806734756 | 5.16688E-05 | 0.008151371 |
| ENSRNOG00000010597 | Slc5a7 | 0.142269283 | -2.813303884 | 0.000275655 | 0.021284137 |
| ENSRNOG00000018385 | Chrm1 | 0.141933152 | -2.816716488 | 0.000329929 | 0.023373885 |
| ENSRNOG00000024310 | Kcnf1 | 0.14120382 | -2.82414898 | 3.80754E-05 | 0.007065383 |
| ENSRNOG00000010065 | Dgkh | 0.140728593 | -2.829012617 | 0.000961631 | 0.042902619 |
| ENSRNOG00000046851 | Tmem121b | 0.140384499 | -2.832544447 | 5.87071E-05 | 0.008700465 |
| ENSRNOG00000014320 | Inhba | 0.139517333 | -2.841483724 | 0.000129732 | 0.013772748 |
| ENSRNOG00000016267 | Chst15 | 0.136483567 | -2.873200842 | 0.000137601 | 0.014198673 |
| ENSRNOG00000026679 | Scn4b | 0.13616397 | -2.876583092 | 2.4701E-05 | 0.005430592 |
| ENSRNOG00000018471 | LOC100362339 | 0.135335242 | -2.885390517 | 0.000195866 | 0.017581739 |
| ENSRNOG00000017766 | Ca12 | 0.135333011 | -2.885414307 | 2.78911E-05 | 0.005810295 |
| ENSRNOG00000049070 | Rack1 | 0.130721743 | -2.935428969 | 3.01433E-20 | 6.53442E-16 |
| ENSRNOG00000056476 | Slc22a13 | 0.129269572 | -2.951545365 | 0.000157738 | 0.015344381 |
| ENSRNOG00000018250 | Tnni3 | 0.129151757 | -2.95286082 | 0.000638924 | 0.033752925 |
| ENSRNOG00000028436 | Rprml | 0.125757321 | -2.9912857 | 0.00020756 | 0.018187846 |
| ENSRNOG00000018790 | Kcnh4 | 0.125754326 | -2.991320069 | 0.000117504 | 0.012845153 |
| ENSRNOG00000012573 | Dlgap2 | 0.125136039 | -2.998430753 | 6.70438E-06 | 0.002349937 |
| ENSRNOG00000060775 | Lmo7 | 0.124765511 | -3.002708909 | 3.44918E-05 | 0.006623021 |
| ENSRNOG00000060061 | Sowaha | 0.124749077 | -3.002898951 | 0.000111359 | 0.012379624 |
| ENSRNOG00000026036 | Pdyn | 0.124232739 | -3.008882684 | 2.9197E-05 | 0.006025492 |
| ENSRNOG00000014006 | Neto1 | 0.122862316 | -3.024885616 | 3.0432E-05 | 0.00618687 |
| ENSRNOG00000001701 | Cbr3 | 0.122137717 | -3.03341931 | 4.76903E-05 | 0.008013031 |
| ENSRNOG00000053592 | AABR07050646.1 | 0.121429934 | -3.041803987 | 0.000182115 | 0.01689689 |
| ENSRNOG00000010412 | Ccdc180 | 0.121366657 | -3.042555973 | 0.000109808 | 0.012379624 |
| ENSRNOG00000024061 | Rarb | 0.120495302 | -3.052951203 | 1.45881E-05 | 0.003604974 |
| ENSRNOG00000027463 | Adamts3 | 0.118609734 | -3.075705681 | 5.13418E-05 | 0.008151371 |
| ENSRNOG00000030101 | Traip | 0.117637995 | -3.087573996 | 0.000759958 | 0.037291998 |
| ENSRNOG00000014296 | Syt10 | 0.117626169 | -3.087719032 | 0.000149365 | 0.014857119 |
| ENSRNOG00000008834 | Dach1 | 0.117541136 | -3.088762346 | 5.01186E-05 | 0.008137645 |
| ENSRNOG00000051487 | Kremen1 | 0.115057505 | -3.119572998 | 1.52524E-05 | 0.003660914 |
| ENSRNOG00000007374 | Tac1 | 0.113733991 | -3.136264603 | 5.9277E-06 | 0.002111218 |
| ENSRNOG00000008785 | Klf5 | 0.112615052 | -3.150528427 | 6.31121E-05 | 0.009168688 |
| ENSRNOG00000056454 | AC096600.1 | 0.112185998 | -3.156035473 | 9.04601E-05 | 0.011159437 |
| ENSRNOG00000049761 | Htr6 | 0.110752031 | -3.174594937 | 5.27872E-05 | 0.008151371 |
| ENSRNOG00000004117 | Kcnv1 | 0.110617718 | -3.176345604 | 0.00011042 | 0.012379624 |
| ENSRNOG00000037960 | Stum | 0.10897315 | -3.197955382 | 0.000116608 | 0.012810583 |
| ENSRNOG00000023712 | Stox1 | 0.106280254 | -3.234054514 | 0.000176821 | 0.016474957 |
| ENSRNOG00000007581 | Slc17a8 | 0.106145659 | -3.235882727 | 8.33121E-05 | 0.010742835 |
| ENSRNOG00000025037 | Ankk1 | 0.1052429 | -3.248205187 | 9.91328E-05 | 0.01183271 |
| ENSRNOG00000011646 | Rem2 | 0.104881726 | -3.253164758 | 7.23117E-05 | 0.009752468 |
| ENSRNOG00000027894 | Iqgap3 | 0.103116506 | -3.277652811 | 1.52499E-05 | 0.003660914 |
| ENSRNOG00000051221 | AABR07070310.1 | 0.101414578 | -3.301663046 | 0.001050025 | 0.04505739 |
| ENSRNOG00000023337 | Sema3a | 0.10045515 | -3.315376573 | 1.31504E-05 | 0.003390923 |
| ENSRNOG00000027606 | Neurl1b | 0.099956253 | -3.32255937 | 1.41418E-05 | 0.003548626 |
| ENSRNOG00000018282 | Gda | 0.099050018 | -3.335698951 | 8.58659E-06 | 0.002685418 |
| ENSRNOG00000009299 | Adra2c | 0.09649015 | -3.373474512 | 3.75426E-06 | 0.001454415 |
| ENSRNOG00000047367 | Card14 | 0.095806106 | -3.383738579 | 6.01073E-09 | 7.37383E-06 |
| ENSRNOG00000036880 | Arl5c | 0.095595579 | -3.386912284 | 0.000634092 | 0.033658687 |
| ENSRNOG00000062261 | AC111804.2 | 0.09536765 | -3.390356226 | 3.93053E-05 | 0.007232838 |
| ENSRNOG00000004730 | Meis2 | 0.095252611 | -3.392097556 | 2.73044E-06 | 0.001116549 |
| ENSRNOG00000004049 | Baiap2 | 0.091357487 | -3.452333218 | 0.000553331 | 0.031170047 |
| ENSRNOG00000011824 | Trh | 0.090084143 | -3.472583014 | 1.29537E-05 | 0.003390923 |
| ENSRNOG00000043465 | Arc | 0.089648864 | -3.479570891 | 0.000608353 | 0.032522986 |
| ENSRNOG00000013282 | Mctp1 | 0.089626682 | -3.479927896 | 0.000580632 | 0.031815173 |
| ENSRNOG00000042321 | AABR07052588.1 | 0.088469582 | -3.498674684 | 5.76943E-06 | 0.002111218 |
| ENSRNOG00000021510 | Tbc1d10c | 0.088318715 | -3.501137009 | 7.20766E-05 | 0.009752468 |
| ENSRNOG00000036661 | Rab40b | 0.088288331 | -3.501633425 | 0.000861114 | 0.04018666 |
| ENSRNOG00000015616 | Rgs14 | 0.087832854 | -3.509095514 | 8.15758E-06 | 0.002610661 |
| ENSRNOG00000004273 | Ifitm1 | 0.086877049 | -3.524881088 | 0.000202422 | 0.018023752 |
| ENSRNOG00000052510 | Wnt10a | 0.085831068 | -3.542356247 | 6.85485E-05 | 0.009551471 |
| ENSRNOG00000011369 | Kcns2 | 0.085307736 | -3.551179619 | 2.63297E-05 | 0.005588722 |
| ENSRNOG00000017833 | Actn2 | 0.084978552 | -3.55675743 | 0.000553059 | 0.031170047 |
| ENSRNOG00000016653 | Ngef | 0.083486627 | -3.582311072 | 0.000887422 | 0.040655699 |
| ENSRNOG00000055673 | Gpr52 | 0.083276363 | -3.585949123 | 0.000984674 | 0.043399701 |
| ENSRNOG00000024259 | Tmem54 | 0.082790618 | -3.594388898 | 0.000595875 | 0.03209295 |
| ENSRNOG00000020694 | Icam5 | 0.082311834 | -3.602756336 | 0.000544505 | 0.031077436 |
| ENSRNOG00000021217 | Itga10 | 0.080624677 | -3.632634707 | 0.000562555 | 0.031280926 |
| ENSRNOG00000009206 | Fezf2 | 0.074478672 | -3.747028849 | 0.000135677 | 0.014198673 |
| ENSRNOG00000033215 | RT1-Db1 | 0.074467349 | -3.747248192 | 0.000620517 | 0.03301748 |
| ENSRNOG00000028404 | Ppp1r1b | 0.073387588 | -3.768320109 | 0.000416439 | 0.026692731 |
| ENSRNOG00000021234 | Slc4a11 | 0.072237147 | -3.791115279 | 2.69786E-06 | 0.001116549 |
| ENSRNOG00000006410 | Akap5 | 0.071992833 | -3.796002892 | 0.000319948 | 0.023132404 |
| ENSRNOG00000005770 | Sostdc1 | 0.071075385 | -3.814506174 | 0.00087486 | 0.040585433 |
| ENSRNOG00000004731 | Ano3 | 0.070788442 | -3.820342364 | 0.000685501 | 0.035021327 |
| ENSRNOG00000006860 | Itk | 0.070622083 | -3.823736814 | 2.53627E-05 | 0.00543746 |
| ENSRNOG00000014761 | Rasd2 | 0.069828635 | -3.840037425 | 0.000872905 | 0.040579992 |
| ENSRNOG00000006588 | Meox2 | 0.068932755 | -3.858666517 | 0.000995601 | 0.043534383 |
| ENSRNOG00000055934 | Dmkn | 0.068037653 | -3.877522808 | 5.88388E-06 | 0.002111218 |
| ENSRNOG00000007816 | AC131483.1 | 0.067545477 | -3.887997026 | 0.000989003 | 0.043399701 |
| ENSRNOG00000010888 | Ankrd33b | 0.064739413 | -3.949211913 | 0.000103848 | 0.012133239 |
| ENSRNOG00000011631 | Fst | 0.064450604 | -3.955662302 | 0.001131154 | 0.046951404 |
| ENSRNOG00000005776 | Bcl11b | 0.063874841 | -3.968608399 | 0.000584322 | 0.031859249 |
| ENSRNOG00000008356 | Myo5c | 0.062931812 | -3.990066704 | 0.001112205 | 0.046514625 |
| ENSRNOG00000011332 | Clspn | 0.060675691 | -4.042737553 | 3.05393E-05 | 0.00618687 |
| ENSRNOG00000010822 | Dlx6 | 0.058607665 | -4.092766828 | 0.00030703 | 0.022982485 |
| ENSRNOG00000019857 | Gng7 | 0.057893171 | -4.110463015 | 0.000414211 | 0.026692731 |
| ENSRNOG00000011310 | Pde10a | 0.057181528 | -4.128307028 | 0.000482581 | 0.029292212 |
| ENSRNOG00000052753 | LOC100912611 | 0.057176691 | -4.12842907 | 0.000557479 | 0.031280926 |
| ENSRNOG00000059605 | Ddn | 0.056018748 | -4.157946452 | 0.000491802 | 0.029370173 |
| ENSRNOG00000004772 | Cytip | 0.055985313 | -4.158807782 | 1.02755E-07 | 6.87588E-05 |
| ENSRNOG00000062141 | Slc18a3 | 0.055699539 | -4.166190791 | 9.11645E-06 | 0.002685418 |
| ENSRNOG00000018752 | Clcf1 | 0.054924601 | -4.18640371 | 5.26156E-05 | 0.008151371 |
| ENSRNOG00000008943 | Penk | 0.054563368 | -4.195923496 | 0.000409399 | 0.026621166 |
| ENSRNOG00000005284 | Itpka | 0.054550331 | -4.196268249 | 0.000466391 | 0.028687597 |
| ENSRNOG00000004229 | Tac3 | 0.052690895 | -4.246302498 | 1.09982E-05 | 0.003113614 |
| ENSRNOG00000032018 | Tmem200b | 0.052225988 | -4.25908831 | 2.65744E-05 | 0.005588722 |
| ENSRNOG00000061215 | Crym | 0.050855262 | -4.297459126 | 0.000349026 | 0.024389876 |
| ENSRNOG00000005277 | Ptprv | 0.04745808 | -4.397202451 | 3.15159E-06 | 0.001265337 |
| ENSRNOG00000021670 | Frem2 | 0.04209313 | -4.5702714 | 3.09098E-08 | 2.84395E-05 |
| ENSRNOG00000057315 | Kcnh3 | 0.040249933 | -4.634869796 | 8.95736E-05 | 0.011159437 |
| ENSRNOG00000001302 | Adora2a | 0.039084851 | -4.677246663 | 0.000331175 | 0.023373885 |
| ENSRNOG00000000640 | Egr2 | 0.037054537 | -4.754205993 | 9.59061E-09 | 1.0589E-05 |
| ENSRNOG00000026953 | Gpr88 | 0.036845601 | -4.762363789 | 0.000546812 | 0.031077436 |
| ENSRNOG00000003800 | Rgs9 | 0.036736639 | -4.766636544 | 0.000214886 | 0.018580108 |
| ENSRNOG00000050223 | Rin1 | 0.036683743 | -4.768715335 | 0.000136651 | 0.014198673 |
| ENSRNOG00000030180 | Lrrc10b | 0.035506948 | -4.815754842 | 0.000217931 | 0.018580108 |
| ENSRNOG00000023688 | Drd1 | 0.034755651 | -4.84660862 | 0.000228116 | 0.018954478 |
| ENSRNOG00000020133 | LOC108348044 | 0.033353678 | -4.90601033 | 6.75293E-05 | 0.009497972 |
| ENSRNOG00000003171 | Mpz | 0.033091585 | -4.917391808 | 0.000163662 | 0.015510655 |
| ENSRNOG00000013869 | Kcnj4 | 0.02819518 | -5.148407617 | 0.000159894 | 0.015400498 |
| ENSRNOG00000047891 | Foxg1 | 0.027843823 | -5.166498874 | 5.2107E-05 | 0.008151371 |
| ENSRNOG00000015719 | Egr4 | 0.027501506 | -5.184345588 | 0.000135617 | 0.014198673 |
| ENSRNOG00000042446 | Ankrd63 | 0.02537396 | -5.300507514 | 0.000162354 | 0.015453047 |
| ENSRNOG00000018780 | Sh3rf2 | 0.024542717 | -5.348561215 | 4.8231E-05 | 0.008013031 |
| ENSRNOG00000048706 | Nox1 | 0.024277297 | -5.364248384 | 0.00031463 | 0.023132404 |
| ENSRNOG00000010312 | Bhlhe23 | 0.020750892 | -5.590682823 | 0.000293042 | 0.022236975 |
| ENSRNOG00000005996 | Lhx6 | 0.018028335 | -5.793590033 | 0.000126503 | 0.0134949 |
| ENSRNOG00000008046 | Tmem30b | 0.014690918 | -6.088931615 | 1.35264E-05 | 0.003433215 |
| ENSRNOG00000061256 | AABR07006888.1 | 0.001300576 | -9.586633406 | 0.000111564 | 0.012379624 |
| ENSRNOG00000012609 | Trdn | 0.001017147 | -9.941255611 | 1.32062E-05 | 0.003390923 |
| ENSRNOG00000008031 | Cacna2d4 | 0.000940819 | -10.05379468 | 1.31305E-05 | 0.003390923 |
| ENSRNOG00000004692 | A1bg | 0.000936642 | -10.06021467 | 0.000484816 | 0.029330704 |
| ENSRNOG00000038286 | Slamf6 | 0.000867232 | -10.17129358 | 0.000935369 | 0.042073698 |
| ENSRNOG00000052491 | AABR07060620.1 | 0.000586515 | -10.73554419 | 0.000880329 | 0.040655699 |
| ENSRNOG00000011616 | Slc26a5 | 0.000564506 | -10.79072317 | 4.16909E-05 | 0.007484712 |
| ENSRNOG00000023214 | Il20rb | 0.000516753 | -10.91823714 | 0.000828225 | 0.039415648 |
| ENSRNOG00000047027 | Unc93a | 0.000418484 | -11.22253934 | 3.59392E-05 | 0.006782987 |
| ENSRNOG00000011263 | Plac9 | 0.00035486 | -11.46046443 | 0.000218929 | 0.018580108 |
| ENSRNOG00000032554 | Scd4 | 0.000324271 | -11.5905126 | 4.2152E-05 | 0.007505117 |
| ENSRNOG00000029401 | Actg2 | 0.000315966 | -11.62794268 | 1.30126E-05 | 0.003390923 |
| ENSRNOG00000008146 | Tox2 | 2.655223048 | 1.408833058 | 0.001174917 | 0.048045393 |
| ENSRNOG00000024028 | Sprr1a | 0.00031526 | -11.63116948 | 0.000935521 | 0.042073698 |
| ENSRNOG00000003245 | Cacng1 | 0.000294562 | -11.72914061 | 5.4924E-05 | 0.00825056 |
| ENSRNOG00000026634 | Glipr1l1 | 0.000185272 | -12.39806493 | 7.0373E-06 | 0.002421225 |
| ENSRNOG00000015403 | Cd52 | 0.000177082 | -12.46329617 | 9.26689E-05 | 0.011332266 |
| ENSRNOG00000050690 | Nr2e3 | 0.000136633 | -12.83740389 | 0.001136966 | 0.047104082 |
