# Supplementary material for: Integrated network pharmacology and transcriptomics to explore the mechanism of compound Dihuang granule (CDG) protects dopaminergic neurons by regulating the Nrf2/HMOX1 pathway in the 6-OHDA/MPP+-induced model of Parkinson’s disease
Source: Chin Med. 2024 Dec 18;19:170. doi: 10.1186/s13020-024-01040-7 (PMC11654441; doi:10.1186/s13020-024-01040-7)
Supplement: Supplementary file 10 — Supplementary Material 10. [file 13020_2024_1040_MOESM10_ESM.docx]

| **List of common differentially expressed genes analysed by Sham VS Model ＆ Model VS CDG RNA-seq** | | | | | |
| --- | --- | --- | --- | --- | --- |
| **Gene ID** | **Gene Name** | **Gene ID** | **Gene Name** | **Gene ID** | **Gene Name** |
| ENSRNOG00000004692 | A1bg | ENSRNOG00000004898 | Fshb | ENSRNOG00000007575 | Plppr1 |
| ENSRNOG00000060406 | AABR07017159.1 | ENSRNOG00000022619 | Fth1 | ENSRNOG00000007324 | Plxna2 |
| ENSRNOG00000024294 | AABR07019083.1 | ENSRNOG00000008431 | Gabbr2 | ENSRNOG00000013322 | Pola1 |
| ENSRNOG00000014264 | AABR07027306.1 | ENSRNOG00000002349 | Gabra2 | ENSRNOG00000047686 | Pou3f1 |
| ENSRNOG00000052899 | AABR07049886.2 | ENSRNOG00000061182 | Gabre | ENSRNOG00000028404 | Ppp1r1b |
| ENSRNOG00000053592 | AABR07050646.1 | ENSRNOG00000049361 | Gas7 | ENSRNOG00000009882 | Ppp3ca |
| ENSRNOG00000042321 | AABR07052588.1 | ENSRNOG00000019495 | Gbx2 | ENSRNOG00000045913 | Prdm16 |
| ENSRNOG00000053753 | AABR07070307.1 | ENSRNOG00000018282 | Gda | ENSRNOG00000003120 | Prelp |
| ENSRNOG00000051221 | AABR07070310.1 | ENSRNOG00000011599 | Gldc | ENSRNOG00000004873 | Prkch |
| ENSRNOG00000062252 | AABR07072853.5 | ENSRNOG00000019857 | Gng7 | ENSRNOG00000019435 | Psd |
| ENSRNOG00000056454 | AC096600.1 | ENSRNOG00000027658 | Gpr101 | ENSRNOG00000002525 | Ptgs2 |
| ENSRNOG00000062261 | AC111804.2 | ENSRNOG00000055673 | Gpr52 | ENSRNOG00000013981 | Ptpn5 |
| ENSRNOG00000007816 | AC131483.1 | ENSRNOG00000026953 | Gpr88 | ENSRNOG00000005807 | Ptpn7 |
| ENSRNOG00000046261 | Acp5 | ENSRNOG00000023657 | Gprin3 | ENSRNOG00000005277 | Ptprv |
| ENSRNOG00000017786 | Acta1 | ENSRNOG00000013604 | Gpx4 | ENSRNOG00000007364 | Rab15 |
| ENSRNOG00000056756 | Actn1 | ENSRNOG00000007346 | Grasp | ENSRNOG00000036661 | Rab40b |
| ENSRNOG00000017833 | Actn2 | ENSRNOG00000001575 | Grik1 | ENSRNOG00000049070 | Rack1 |
| ENSRNOG00000027463 | Adamts3 | ENSRNOG00000013171 | Grm2 | ENSRNOG00000024061 | Rarb |
| ENSRNOG00000059479 | Adcy1 | ENSRNOG00000016429 | Grm5 | ENSRNOG00000024705 | Rarres2 |
| ENSRNOG00000002229 | Adcy5 | ENSRNOG00000016999 | Grp | ENSRNOG00000014761 | Rasd2 |
| ENSRNOG00000001302 | Adora2a | ENSRNOG00000012302 | Gucy1a1 | ENSRNOG00000031671 | Rasgef1a |
| ENSRNOG00000009299 | Adra2c | ENSRNOG00000014117 | Hmox1 | ENSRNOG00000021098 | Rasgrp2 |
| ENSRNOG00000002232 | Aff1 | ENSRNOG00000049761 | Htr6 | ENSRNOG00000011646 | Rem2 |
| ENSRNOG00000025584 | Agap2 | ENSRNOG00000020694 | Icam5 | ENSRNOG00000014424 | RGD1563354 |
| ENSRNOG00000006410 | Akap5 | ENSRNOG00000004273 | Ifitm1 | ENSRNOG00000055564 | RGD1564664 |
| ENSRNOG00000030869 | Aldoart2 | ENSRNOG00000016308 | Il10ra | ENSRNOG00000007949 | Rgn |
| ENSRNOG00000025037 | Ankk1 | ENSRNOG00000028650 | Inf2 | ENSRNOG00000015616 | Rgs14 |
| ENSRNOG00000010888 | Ankrd33b | ENSRNOG00000014320 | Inhba | ENSRNOG00000003800 | Rgs9 |
| ENSRNOG00000042446 | Ankrd63 | ENSRNOG00000025406 | Iqgap2 | ENSRNOG00000050223 | Rin1 |
| ENSRNOG00000004731 | Ano3 | ENSRNOG00000027894 | Iqgap3 | ENSRNOG00000003479 | Rnf150 |
| ENSRNOG00000043465 | Arc | ENSRNOG00000006723 | Itga11 | ENSRNOG00000028436 | Rprml |
| ENSRNOG00000024677 | Arhgap33 | ENSRNOG00000022071 | Itga2b | ENSRNOG00000004362 | Rps6ka5 |
| ENSRNOG00000006946 | Arhgap9 | ENSRNOG00000006860 | Itk | ENSRNOG00000015701 | Rreb1 |
| ENSRNOG00000011105 | Arl15 | ENSRNOG00000005284 | Itpka | ENSRNOG00000061526 | Rsph6a |
| ENSRNOG00000020770 | Arl4d | ENSRNOG00000007104 | Itpr1 | ENSRNOG00000033215 | RT1-Db1 |
| ENSRNOG00000036880 | Arl5c | ENSRNOG00000019719 | Kcna5 | ENSRNOG00000003809 | Sat1 |
| ENSRNOG00000002256 | Art3 | ENSRNOG00000056697 | Kcnab1 | ENSRNOG00000016177 | Scara3 |
| ENSRNOG00000051619 | Asb2 | ENSRNOG00000024310 | Kcnf1 | ENSRNOG00000032554 | Scd4 |
| ENSRNOG00000019985 | Asic4 | ENSRNOG00000054314 | Kcng1 | ENSRNOG00000015055 | Scg2 |
| ENSRNOG00000004026 | Atp2b1 | ENSRNOG00000003841 | Kcnh1 | ENSRNOG00000026679 | Scn4b |
| ENSRNOG00000004049 | Baiap2 | ENSRNOG00000057315 | Kcnh3 | ENSRNOG00000023337 | Sema3a |
| ENSRNOG00000005776 | Bcl11b | ENSRNOG00000018790 | Kcnh4 | ENSRNOG00000006263 | Sh2d1a |
| ENSRNOG00000001304 | Bcr | ENSRNOG00000013869 | Kcnj4 | ENSRNOG00000018780 | Sh3rf2 |
| ENSRNOG00000013717 | Bmp6 | ENSRNOG00000002653 | Kcnk2 | ENSRNOG00000016877 | Shisa7 |
| ENSRNOG00000042163 | Btbd19 | ENSRNOG00000013781 | Kcnq5 | ENSRNOG00000007646 | Sipa1l1 |
| ENSRNOG00000017766 | Ca12 | ENSRNOG00000011369 | Kcns2 | ENSRNOG00000026091 | Slc10a4 |
| ENSRNOG00000003245 | Cacng1 | ENSRNOG00000004117 | Kcnv1 | ENSRNOG00000007581 | Slc17a8 |
| ENSRNOG00000017882 | Camk1d | ENSRNOG00000016467 | Kctd1 | ENSRNOG00000062141 | Slc18a3 |
| ENSRNOG00000047367 | Card14 | ENSRNOG00000033694 | Klf16 | ENSRNOG00000005479 | Slc1a2 |
| ENSRNOG00000001701 | Cbr3 | ENSRNOG00000008785 | Klf5 | ENSRNOG00000060687 | Slc24a3 |
| ENSRNOG00000010412 | Ccdc180 | ENSRNOG00000029441 | Klhl2 | ENSRNOG00000006729 | Slc24a4 |
| ENSRNOG00000019321 | Cck | ENSRNOG00000024479 | Klhl34 | ENSRNOG00000009832 | Slc39a14 |
| ENSRNOG00000015036 | Ccn2 | ENSRNOG00000051487 | Kremen1 | ENSRNOG00000012508 | Slc39a8 |
| ENSRNOG00000007483 | Ccnf | ENSRNOG00000049495 | Krt71 | ENSRNOG00000021234 | Slc4a11 |
| ENSRNOG00000004148 | Cdk17 | ENSRNOG00000005457 | Lamp5 | ENSRNOG00000010597 | Slc5a7 |
| ENSRNOG00000057347 | Cebpb | ENSRNOG00000016879 | Ldlrad4 | ENSRNOG00000010210 | Slc7a11 |
| ENSRNOG00000018385 | Chrm1 | ENSRNOG00000060775 | Lmo7 | ENSRNOG00000007377 | Slit3 |
| ENSRNOG00000016267 | Chst15 | ENSRNOG00000020133 | LOC108348044 | ENSRNOG00000008620 | Smad3 |
| ENSRNOG00000018752 | Clcf1 | ENSRNOG00000008680 | Loxl1 | ENSRNOG00000000257 | Smpd3 |
| ENSRNOG00000003654 | Cldn9 | ENSRNOG00000012181 | Lpl | ENSRNOG00000042326 | Smpdl3b |
| ENSRNOG00000011332 | Clspn | ENSRNOG00000030180 | Lrrc10b | ENSRNOG00000005770 | Sostdc1 |
| ENSRNOG00000023803 | Cmya5 | ENSRNOG00000004048 | Lrrk2 | ENSRNOG00000060061 | Sowaha |
| ENSRNOG00000007014 | Cnksr2 | ENSRNOG00000011826 | Lzts1 | ENSRNOG00000016388 | Sphkap |
| ENSRNOG00000008223 | Cnr1 | ENSRNOG00000021231 | Lzts3 | ENSRNOG00000024028 | Sprr1a |
| ENSRNOG00000005286 | Coch | ENSRNOG00000000800 | Man1a1 | ENSRNOG00000058842 | Sptbn2 |
| ENSRNOG00000001229 | Col18a1 | ENSRNOG00000017905 | Map1lc3b | ENSRNOG00000007879 | Stk26 |
| ENSRNOG00000011913 | Cp | ENSRNOG00000015401 | Mapk4 | ENSRNOG00000023712 | Stox1 |
| ENSRNOG00000000522 | Cpne5 | ENSRNOG00000003703 | Mcm6 | ENSRNOG00000008312 | Stra6 |
| ENSRNOG00000015397 | Cpne7 | ENSRNOG00000013282 | Mctp1 | ENSRNOG00000037960 | Stum |
| ENSRNOG00000023633 | Crabp1 | ENSRNOG00000053787 | Mdfic | ENSRNOG00000009037 | Sulf1 |
| ENSRNOG00000061215 | Crym | ENSRNOG00000004730 | Meis2 | ENSRNOG00000019181 | Synpo |
| ENSRNOG00000003622 | Cybb | ENSRNOG00000006588 | Meox2 | ENSRNOG00000008203 | Synpr |
| ENSRNOG00000004772 | Cytip | ENSRNOG00000005934 | Mlip | ENSRNOG00000014296 | Syt10 |
| ENSRNOG00000008834 | Dach1 | ENSRNOG00000009514 | Mme | ENSRNOG00000019163 | Syt6 |
| ENSRNOG00000033026 | Dclk3 | ENSRNOG00000003171 | Mpz | ENSRNOG00000007374 | Tac1 |
| ENSRNOG00000059605 | Ddn | ENSRNOG00000056817 | Muc6 | ENSRNOG00000004229 | Tac3 |
| ENSRNOG00000023465 | Depp1 | ENSRNOG00000019627 | Mybpc2 | ENSRNOG00000021510 | Tbc1d10c |
| ENSRNOG00000010065 | Dgkh | ENSRNOG00000049695 | Myh4 | ENSRNOG00000049758 | Tbc1d16 |
| ENSRNOG00000012573 | Dlgap2 | ENSRNOG00000016983 | Myh7 | ENSRNOG00000001128 | Tesc |
| ENSRNOG00000010822 | Dlx6 | ENSRNOG00000008356 | Myo5c | ENSRNOG00000030625 | Tf |
| ENSRNOG00000055934 | Dmkn | ENSRNOG00000008415 | Nab2 | ENSRNOG00000016728 | Tiam2 |
| ENSRNOG00000005451 | Dnah11 | ENSRNOG00000019768 | Ncoa4 | ENSRNOG00000046851 | Tmem121b |
| ENSRNOG00000021573 | Dpy19l3 | ENSRNOG00000014006 | Neto1 | ENSRNOG00000032018 | Tmem200b |
| ENSRNOG00000025860 | Drc7 | ENSRNOG00000027606 | Neurl1b | ENSRNOG00000024259 | Tmem54 |
| ENSRNOG00000023688 | Drd1 | ENSRNOG00000003872 | NEWGENE_620180 | ENSRNOG00000010165 | Tnfaip2 |
| ENSRNOG00000014648 | Efnb2 | ENSRNOG00000016653 | Ngef | ENSRNOG00000018250 | Tnni3 |
| ENSRNOG00000000640 | Egr2 | ENSRNOG00000015863 | Npsr1 | ENSRNOG00000020332 | Tnnt3 |
| ENSRNOG00000015719 | Egr4 | ENSRNOG00000001548 | Nrf2 | ENSRNOG00000010777 | Tox |
| ENSRNOG00000023389 | Ephx4 | ENSRNOG00000009243 | Oaf | ENSRNOG00000008146 | Tox2 |
| ENSRNOG00000033261 | Fam107a | ENSRNOG00000011310 | Pde10a | ENSRNOG00000030101 | Traip |
| ENSRNOG00000052758 | Fam49a | ENSRNOG00000019560 | Pde2a | ENSRNOG00000021091 | Trank1 |
| ENSRNOG00000011774 | Fblim1 | ENSRNOG00000013436 | Pde7b | ENSRNOG00000011824 | Trh |
| ENSRNOG00000009206 | Fezf2 | ENSRNOG00000010280 | Pde8b | ENSRNOG00000047027 | Unc93a |
| ENSRNOG00000011521 | Filip1 | ENSRNOG00000026036 | Pdyn | ENSRNOG00000025110 | Vwa3a |
| ENSRNOG00000019902 | Folr1 | ENSRNOG00000008943 | Penk | ENSRNOG00000048847 | Wdr17 |
| ENSRNOG00000046667 | Fosb | ENSRNOG00000000525 | Pi16 | ENSRNOG00000005108 | Wfs1 |
| ENSRNOG00000047891 | Foxg1 | ENSRNOG00000016846 | Pik3cd | ENSRNOG00000020441 | Wnk4 |
| ENSRNOG00000013397 | Foxo1 | ENSRNOG00000011263 | Plac9 | ENSRNOG00000052510 | Wnt10a |
| ENSRNOG00000009184 | Foxp1 | ENSRNOG00000004810 | Plcb1 | ENSRNOG00000014385 | Wnt2b |
| ENSRNOG00000021670 | Frem2 | ENSRNOG00000033119 | Plcb4 | ENSRNOG00000006972 | Zfp189 |
| ENSRNOG00000007329 | Frmd6 | ENSRNOG00000011951 | Plk2 |  |  |
